# Plant virome reconstruction and antiviral RNAi characterization by deep sequencing of small RNAs from dried leaves

**DOI:** 10.1038/s41598-019-55547-3

**Published:** 2019-12-17

**Authors:** Victor Golyaev, Thierry Candresse, Frank Rabenstein, Mikhail M. Pooggin

**Affiliations:** 1 BGPI, INRA Centre Occitanie, CIRAD, SupAgro, Université de Montpellier, Montpellier, 34984 France; 2 UMR 1332 Biologie du Fruit et Pathologie, INRA, Univ. Bordeaux, CS20032, Villenave d’Ornon cedex, 33882 France; 3 Julius Kühn-Institut, Bundesforschungsinstitut für Kulturpflanzen, Erwin-Baur-Straße 27, Quedlinburg, 06484 Germany

**Keywords:** Molecular biology, Plant sciences

## Abstract

In plants, RNA interference (RNAi) generates small interfering (si)RNAs from entire genomes of viruses, satellites and viroids. Therefore, deep small (s)RNA sequencing is a universal approach for virome reconstruction and RNAi characterization. We tested this approach on dried barley leaves from field surveys. Illumina sequencing of sRNAs from 2 plant samples identified in both plants Hordeum vulgare endornavirus (HvEV) and barley yellow mosaic bymovirus (BaYMV) and, additionally in one plant, a novel strain of Japanese soil-borne wheat mosaic furovirus (JSBWMV). *De novo* and reference-based sRNA assembly yielded complete or near-complete genomic RNAs of these viruses. While plant sRNAs showed broad size distribution, viral sRNAs were predominantly 21 and 22 nucleotides long with 5′-terminal uridine or adenine, and were derived from both genomic strands. These *bona fide* siRNAs are presumably processed from double-stranded RNA precursors by Dicer-like (DCL) 4 and DCL2, respectively, and associated with Argonaute 1 and 2 proteins. For BaYMV (but not HvEV, or JSBWMV), 24-nucleotide sRNAs represented the third most abundant class, suggesting DCL3 contribution to anti-bymovirus defence. Thus, viral siRNAs are well preserved in dried leaf tissues and not contaminated by non-RNAi degradation products, enabling both complete virome reconstruction and inference of RNAi components mediating antiviral defense.

## Introduction

RNA interference (RNAi) is an evolutionarily conserved, small RNA (sRNA)-generating mechanism that regulates gene expression and defends against transposons, transgenes and viruses in most eukaryotes. In plants, virus-derived small interfering RNAs (siRNAs) are generated by Dicer-like (DCL) family proteins from double-stranded RNA (dsRNA) precursors and then sorted by Argonaute (AGO) family proteins, creating RNA-induced silencing complexes (RISCs). RISCs target cognate viral RNAs for cleavage and degradation and, in the case of DNA viruses, also repress viral DNA transcription. Based on evidence from model plants such as Arabidopsis, RNA viruses are targeted predominantly by DCL4 and DCL2, generating 21 and 22 nt siRNAs, which are then sorted by AGO1/5/10 and AGO2/3/7 clade proteins selecting sRNAs with 5′-terminal U (AGO1), A (AGO2), and C (AGO5) (reviewed in ref. ^1^), while DNA viruses are additionally targeted by nuclear DCL3, generating 24 nt siRNAs, which can potentially be associated with AGO4/6/9 clade proteins involved in *de novo* DNA methylation and transcriptional silencing (reviewed in ref. ^2^). Notably, viral siRNAs cover without gaps the entire genome sequences of RNA and DNA viruses in both sense and antisense polarities, allowing for *de novo* reconstruction by deep sRNA sequencing and bioinformatics of complete viral genomes and their genetic variants both in single virus/viroid- and in complex virome-infected plants^3–5^. Furthermore, analysis of sRNA size, polarity, 5′-nt identity and hotspot profiles allows one to infer which DCLs and AGOs mediate antiviral defense and how different types of viruses evade or suppress RNAi in different plant species and families^2^.

For sRNA analysis, total RNA is usually extracted from fresh plant tissues, or from frozen tissues stored at −80 °C, which preserves integrity of functional cellular RNAs by preventing their degradation through various RNA decay pathways. In two reported cases, dried plant tissues were used as starting material for sRNA sequencing in order to identify and reconstruct plant viruses. Hartung and co-workers sequenced sRNAs from dried orange peel samples of herbaria specimens collected in 1948 and 1957 to reconstruct nearly full-length sequences of bi-segmented -ssRNA genome of citrus leprosis virus N (genus *Dichorhavirus*, family *Rhabdoviridae*) and bi-segmented + ssRNA genome of citrus leprosis virus C (genus *Cilevirus*, family *Kitaviridae*)^6^. Smith and co-workers used a ca. 750 year-old barley grain for sRNA sequencing to reconstruct three near-complete + ssRNA genome segments of barley stripe mosaic virus (genus *Hordeivirus*, family *Virgaviridae*)^7^. In both studies, reference-based assembly was implemented, but size, polarity, 5′-nt identity or hotspot distribution profiles of sRNAs mapped to reconstructed viral genomes were not analyzed. Here we extended these previous studies by demonstrating that dried leaf tissues can be used for in-depth characterization of sRNA-ome, enabling virus identification, virome reconstruction and inference of plant RNAi machinery components mediating biogenesis of viral siRNAs.

## Results and Discussion

### Dried leaf virome reconstruction from sRNAs allows to identify new viral strains and genetic variants and trace evolution of viral quasispecies

Samples of barley leaves exhibiting mosaic symptoms collected during field surveys in 2013–2015 (each year from January to April), dried and kept stored over anhydrous calcium chloride at room temperature, were analyzed by high-throughput dsRNA sequencing to evaluate the diversity of bymoviruses in barley^8^ (see below). In August 2016, four of these dried leaf samples were taken for total RNA extraction and quality control using RNA blot hybridization analysis with the plant miRNA miR160-specific probe (as detailed in Methods section below). This analysis showed comparable integrity of miR160 in total RNAs extracted from the dried leaves and from fresh leaves of healthy barley seedlings grown in a phytochamber (Supplementary Fig. S1). The sample HYT-37 obtained from France in 2013 and sample HYT-38 obtained from Germany in 2015 were then selected for Illumina sequencing and bioinformatic analysis of sRNAs. *De novo* assembly of 20–25 nt sRNA reads, followed by BLASTn analysis of the resulting contigs and reference-based reconstruction (as described in Methods) allowed us to identify the following viruses: Hordeum vulgare endornavirus (HvEV, genus *Alphaendornavirus*, family *Endornaviridae*) and barley yellow mosaic virus (BaYMV, genus *Bymovirus*, family *Potyviridae*) in both HYT-37 and HYT-38 samples, and, additionally in the sample HYT-38, a novel virus related to French barley mosaic virus (genus *Furovirus*, family *Virgaviridae*) and other furoviruses (see below). The alphaendornavirus HvEV was represented by multiple short contigs (up to 430 nts), and reference-guided scaffolding of those contigs and gap-filling with sRNA reads allowed us to reconstruct its near complete RNA genome sequence (99.0% in HYT-37 lacking 152 nts of the reference sequence, and 98.1% in HYT-38 lacking 276 nts of the reference). The gaps not covered by viral sRNAs (n = 19 for HYT-37 and n = 23 for HYT-38) range from one to 42 nts and scatter along the 14243 nt genome (see Supplementary Dataset S1). The bymovirus BaYMV was represented by much larger contigs (up to 3493 nts in HYT-37 and up to 3466 nts in HYT-38), and reference-guided contig/sRNA assembly allowed us to reconstruct the complete bi-segmented RNA genome (7563 nt RNA1 and 3516 nt RNA2) from each sample. The novel furovirus in HYT-38 was represented by contigs up to 1700 nts in length and reference-guided contig/sRNA assembly allowed us to reconstruct 99.5% of its bi-segmented RNA genome, with the 7003 nt RNA1 lacking 40 nts of its reference sequence (6 gaps of 3 to 13 nts) and the 3574 nt RNA2 lacking 7 nts at the 5′-end of its reference sequence (see Supplementary Dataset S1). Note that the furovirus RNA1 reference sequence assembled from longer reads of Illumina dsRNA sequencing data (deposited in GenBank as MN123252) may not contain complete 5′- and 3′-termini, whereas the RNA2 reference sequence (KU236377) obtained by convential cloning and Sanger sequencing is likely complete.

Phylogenetic analysis of HvEV isolates revealed that the 14243 nt genome sequences reconstructed from HYT-37 (deposited in GenBank as MN107382) and HYT-38 (deposited in GenBank as MN107383) exhibit 99.7% pairwise identity (50 single nucleotide polymorphisms (SNPs)), and both are ca. 94% identical to the sequence of the only complete HvEV genome available (KT721705)^9^ that we used for reference-based reconstruction. The SNPs randomly scatter along the genome without disruption of a single large polyprotein ORF. HYT-37 and HYT-38 encoded polyproteins exhibit 99.6% pairwise identity and share with the KT721705-encoded protein 97.6% and 97.9% identity, respectively. We conclude that the HYT-37 and HYT-38 isolates of HvEV are closely related to, but distinct from the reference isolate of the virus.

A phylogenetic analysis of BaYMV isolates revealed that the 7563 nt RNA1 sequences reconstructed from HYT-37 (deposited in GenBank as MN107377) and HYT-38 (deposited in GenBank as MN107378) exhibit 98.0% pairwise identity (155 SNPs), and are respectively 97.8% and 98.1% identical to the sequence of an isolate collected in France in 2014 (KX117192)^8^ that we used for reference-based reconstruction, with SNPs not disrupting a single polyprotein ORF. Among other BaYMV isolates, the RNA1 of HYT-37 is 99.0% identical to that of the KX117208 isolate from Germany, while the RNA1 of HYT-38 is 99.0% identical to that of the KX117195 isolate from France. The 3516 nt BaYMV RNA2 sequences reconstructed from HYT-37 (deposited in GenBank as MN107381) and HYT-38 (deposited in GenBank as MN107380) exhibit 95.6% pairwise identity (154 SNPs), and are respectively 95.2% and 98.1% identical to the RNA2 reference sequence obtained by dsRNA sequencing^8^, with SNPs not disrupting the single polyprotein ORF. Furthermore, they exhibit respectively 94.7% (HYT-37) and 97.4% (HYT-38) identity to RNA2 sequences of the HAT (AJ515487) and EST (AJ515486) isolates from UK^10^. Taken together the HYT-37 and HYT-38 isolates of BaYMV from the barley samples are closely related to each other and to other barley isolates obtained from France in 2013 and 2014 and from Germany in 2015, and more distantly related to barley isolates collected in UK more than 10 years earlier, thus highlighting the ongoing evolution of BaYMV with appearance and fixation of new genetic variants over time.

A phylogenetic analysis of the novel furovirus revealed that the 7003 nt RNA1 from HYT-38 (deposited in GenBank as MN123253) is 99.5% identical to the RNA1 reference sequence (deposited in GenBank as MN123252) used for reconstruction and obtained by dsRNA sequencing of another barley plant of the same origin (with 34 SNPs not disrupting the main ORFs), and 98.8% identical to a partial 1507 nt RNA1 sequence of French barley mosaic virus (FBMV; AJ749658) from France^11^. It is also 84.3% identical to the 7226 nt RNA1 of Japanese soil-borne wheat mosaic virus (JSBWMV; NC_038850) from Japan^12^. The furovirus 3574 nt RNA2 from HYT-38 (deposited in GenBank as MN123254) is 98.7% identical to the RNA2 of soil-borne barley mosaic virus (SBBMV) isolate Bornum (KU236377) from Germany^13^, the reference sequence used for reconstruction, with 48 SNPs not disrupting the main ORFs. It is also 98.4% identical to a near-complete FBMV RNA2 from France (AJ749657)^11^, and 94.1% identical to JSBWMV RNA2 from Japan (NC_038851)^12^. The current species demarcation criteria for *Furovirus* genus are less than 75% nucleotide identity for the RNA1 sequence and less than 80% identity for the RNA2 sequence (https://talk.ictvonline.org/ictv-reports/ictv_online_report/positive-sense-rna-viruses/w/virgaviridae/667/genus-furovirus). Based on the genome sequences we obtained, the furovirus present in the HYT-38 sample and another barley sample, together with FBMV and SBBMV isolates, should all be regarded as belonging to the JSBWMV species. Given the higher level of divergence between the Japanese and the European RNA1 sequences (ca. 84.3%) and the fact that the European isolates have all been observed in barley, the latter isolates could be considered to represent a barley strain of JSBWMV. On the other hand, the much higher RNA2 identity level (ca. 94.1%) suggests that genomic reassortment may have contributed to the evolution of these viruses in barley and wheat hosts. Interestingly, one of the Japanese isolates of soil-borne wheat mosaic virus was identified in a naturally infected barley plant, and this barley isolate initially induced mild symptoms in wheat plants but evolved during successive passages in wheat plants to generate a severe genetic variant with a deletion in RNA2^14^.

### Viral sRNA profiles reveal distinct responses of the barley RNAi machinery to different virome components

**Figure 1:**
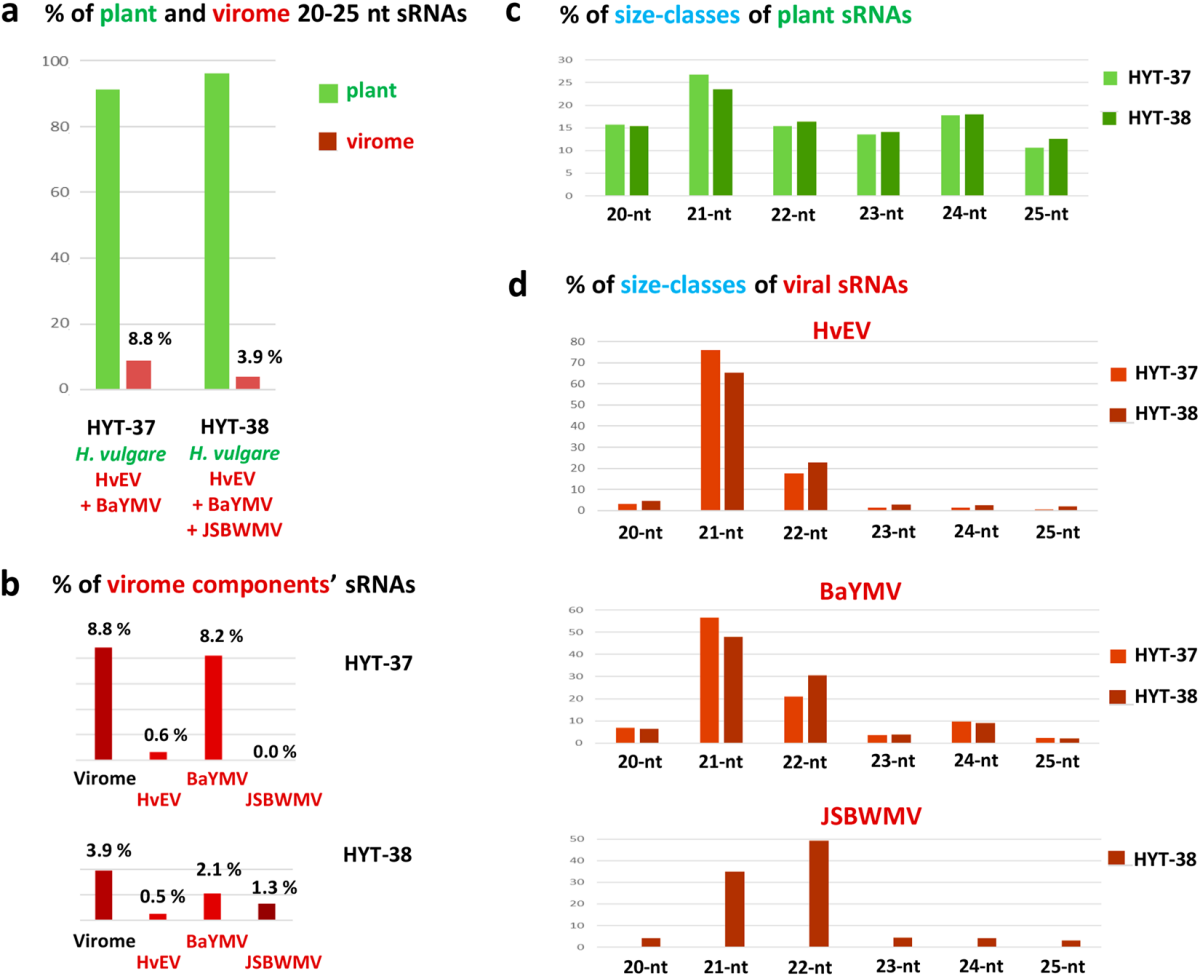
Illumina sequencing counts of endogenous and viral small RNAs (sRNAs) in the dried barley leaves. The 20- to 25-nt sRNA libraries from the dried barley leaf samples HYT-37 and HYT-38 were mapped to the combined virome or each of the individual virus (HvEV, BaYMV, or JSBWMV) reference sequences and to the plant (*Hordeum vulgare*) genome reference sequence with zero mismatches and were counted. (**a**) Percentage of the virome- and the plant-derived sRNAs in the pool of total 20- to 25-nt reads. (**b**) Percentage of each of the individual virus-derived sRNAs in the pool of total 20- to 25-nt reads. (**c**,**d**) Percentage of each size class in the 20- to 25-nt pool of plant-derived (**c**) or each virus-derived (**d**) sRNA reads.

Bioinformatics analysis of the sRNA sequencing data revealed that, in both HYT-37 and HYT-38 libraries (each of ca. 20 M reads with qualities equal to or more than Q30), 39–40% reads fall into a size range of 20 to 25 nts and, within this range, the size distribution profile does not show any strong bias, except that 21 nt class is ca. 1.5- to 2-times more abundant than other classes (Supplementary Dataset S2). Counting sRNA reads mapped to the barley genome also showed that the 21 nt class is the most abundant, whereas the 24 nt class normally dominating together with the 21 nt class (due to 24 nt heterochromatic siRNAs and 21 nt miRNAs, respectively) is only slightly more abundant than the 20, 22 and 23 nt classes (Fig. 1c; Supplementary Dataset S2). In both libraries, 25 nt plant reads are the least abundant but still represent a substantial fraction comparable to the fraction of 23 nt reads that may derive from functional 24 nt siRNAs truncated by one nucleotide. The 25 nt reads may not represent any known functional plant miRNA or siRNA species (or their degradation products) and are therefore expected to be of lower abundance. These data together with high molecular weight RNA profiles of the HYT-37 and HYT-38 samples (Supplementary Figs. S1 and S2) may indicate a substantial amount of non-RNAi degradation products in the sRNA populations. It is worth mentioning that the two dried leaf samples of barley were processed in parallel with freshly frozen leaf samples of *Dactylis glomerata* (from the same family *Poaceae*) at both total RNA extraction and sRNA library preparation steps and then multiplexed together in one lane of Illumina HiSeq (see Methods). The *Dactylis* sRNA size profile was found to be dominated by the 21 nt and 24 nt classes both accumulating at levels eight or more times higher than other classes, with the 25 nt class being the least abundant (Supplementary Dataset S2a). Thus, desiccation and/or storage conditions of the barley leaf samples rather than the downstream processing steps may have resulted in partial degradation of some plant RNA species. The presumptive degradation pathway(s) had generated relatively abundant 25-nt sRNAs possessing both 5′-terminal phosphate and 3′-terminal hydroxyl groups, same as those of DCL-generated functional miRNA and siRNA species targeted by the Illumina sRNA library preparation protocol. However, we cannot rule out the possibility that 25 nt sRNAs (and other non-RNAi products) had been generated in the barley leaves in the field before sampling. In one of the previous Illumina-seq studies of the barley sRNA-ome, fresh leaves of two distinct barley cultivars accumulated only trace amounts of 25-nt sRNAs^15^, while in another study, frozen leaves of three of four barley cultivars accumulated substantial amounts of 25 nt sRNAs^16^. Interestingly, in both of these studies, 21 nt sRNAs (and, in some cultivars, 20 nt sRNAs) were found to be the most abundant, whereas 24 nt sRNAs accumulated at substantially lower levels comparable to those of 22 nt sRNAs.

Mapping sRNA reads to the reconstructed genome sequences of HvEV, BaYMY and JSBWMV with zero mismatches revealed that the virome-derived sRNAs constitute respectively 8.8% (HYT-37) and 3.9% (HYT-38) of total (plant + virome) 20–25 nt reads, with BaYMV reads being the most abundant (8.2% in HYT-37 and 2.1% in HYT-38) and HvEV reads the least abundant (0.6% in HYT-37 and 0.5% in HYT-38). JSBWMV reads constituted 1.3% of total 20–25-nt reads in HYT-38, and were virtually absent in HYT-37 (0.0035%, or 306 reads, which may represent cross-contamination) (Fig. 1a,b; Supplementary Dataset S2a). Size profiling showed for all three viruses the predominance of 21 nt and 22 nt sRNAs, likely representing *bona fide* viral siRNAs generated by barley DCL4- and DCL2-like activities, respectively, while other size-classes were found to be of much lower abundance, with the 25 nt class being the least abundant (Fig. 1d; Supplementary Dataset S2a). Thus, unlike plant sRNAs, viral siRNAs are not contaminated substantially with non-RNAi degradation products of longer viral RNAs. We assume that viral genomic (and subgenomic) RNAs or their replication and transcription intermediates may not be targeted by the degradation pathway(s) that generated abundant 25 nt plant sRNAs in the dried barley leaf samples. Interestingly, for HvEV and BaYMV, 21 nt siRNAs are more abundant than 22 nt siRNAs in both samples, whereas the situation is opposite for JSBWMV. Moreover, the ratio of 21 and 22 nt siRNAs derived from both HvEV and BaYMV is higher in the absence (HYT-37) than in the presence of JSBWMV (HYT-38) (Fig. 1d). Thus, JSBWMV infection appeared to alter the relative DCL4 vs DCL2 antiviral activities.

**Figure 2:**
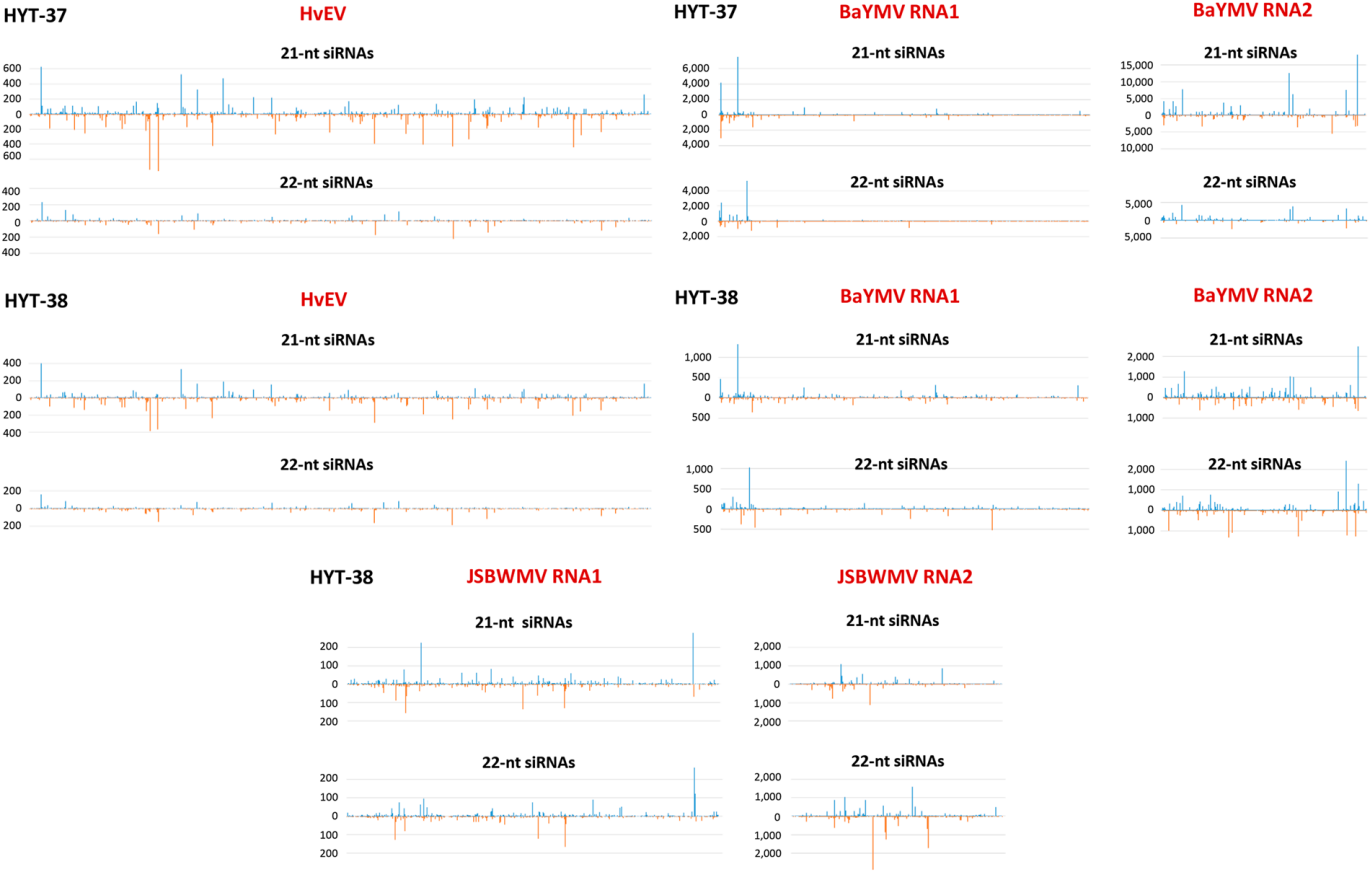
Single-nucleotide resolution maps of virus-derived small interfering RNAs (siRNAs) from the dried barley leaves. For each plant sample (HYT-37 and HYT-38), the histograms plot the numbers of 21 and 22 nt viral siRNA reads at each nucleotide position of HvEV genomic RNA, BaYMV genomic RNA1 and RNA2, and, in the case of HYT-38, JSBWMV genomic RNA1 and RNA2 (mapped with zero mismatches). The bars above the axis represent sense (forward) reads starting at each position and those below represent antisense (reverse) reads ending at the respective position.

Analysis of single nucleotide-resolution maps (Supplementary Dataset S3) revealed that both 21 and 22 nt viral siRNAs are distributed along forward and reverse strands of the entire virus genomes with local hotspots evident on both strands (Fig. 2). Generally, the hotspot regions are shared between size classes with many (but not all) peaks of 21 and 22 nt siRNA species being at the same positions on each strand. The hotspot patterns are distinct for each virus and, in the cases of HvEV and BaYMV, are reproducible between HYT-37 and HYT-38, indicating that viral siRNA biogenesis is governed by differences in viral genome sequences rather than conditions. Thus, siRNA hotspots are randomly distributed along the entire genome of HvEV in both HYT-37 and HYT-38, without any global strand-bias but with distinct, strand-dependent patterns of peaks. In contrast, BaYMV has major siRNA hotspots at the 5′-terminal region of RNA1 and all along RNA2, with more abundant siRNA species derived from the forward strand, although the forward strand bias is more pronounced in HYT-37 than in HYT-38. In JSBWMV, the 5′-terminal regions of RNA1 and RNA2 as well as the 3′-terminal region of RNA2 are devoid of major siRNA hotspots, and there is no global strand-bias (Fig. 2; Supplementary Dataset S2a). Interestingly, in the case of both JSBWMV and BaYMV, RNA2 spawns much more abundant 21 and 22 nt siRNAs than RNA1, which presumably reflects relative accumulation levels of these two genomic segments.

**Figure 3:**
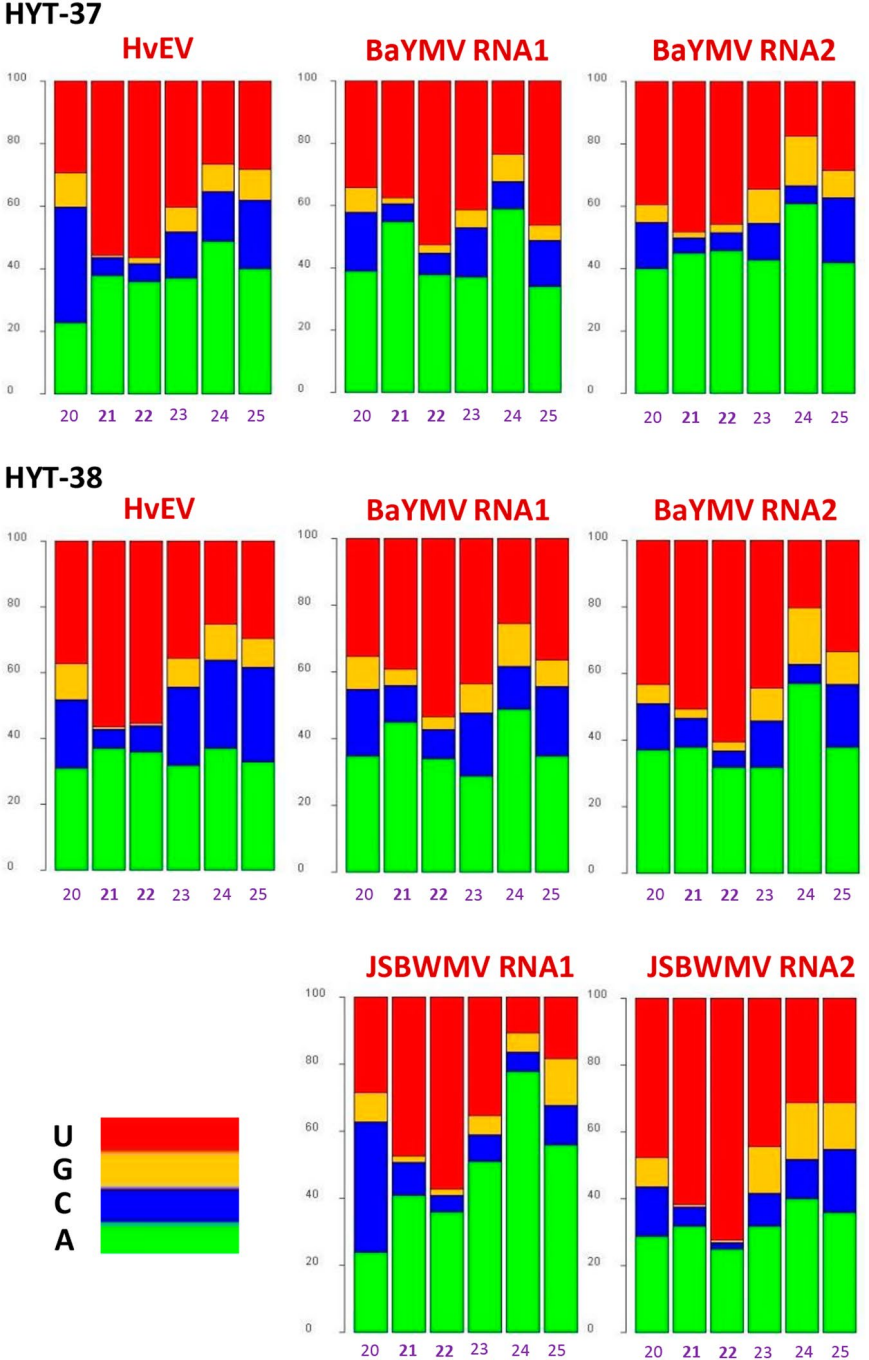
Counts of 5′-terminal nucleotide identity of virus-derived small RNAs (sRNA) in the dried barley leaves. For each plant sample (HYT-37 and HYT-38), the composite bar graphs plot the percentage of 5′U (in red color), 5′G (in orange), 5′C (in blue), and 5′A (in green) for each of the six size-classes (20, 21, 22, 23, 24, and 25 nt) of virus (HvEV, BaYMV RNA1, BaYMV RNA2, JSBWMV RNA1, and JSBWMV RNA2)-derived sRNAs.

Theoretically, differences in siRNA hotspot patterns and strand-biases can stem from sequence preferences of DCL processing, from unequal accumulation levels of dsRNA precursors derived from different regions of viral genome, and/or from AGO-mediated sorting of siRNA duplexes produced by DCLs, which results in stabilization of one of the two strands. We therefore analysed 5′-terminal nucleotide identities of viral sRNAs for each size-class and polarity (Supplementary Dataset S1c). Strikingly, for all viruses, for both major size-classes (21 and 22 nt) and for both polarities (forward and reverse), viral siRNAs possess predominantly 5′U (43–69%) and 5′A (28–49%), together constituting 87–96% reads (depending on virus or condition) (Fig. 3; Supplementary Dataset S2c). Such a strong bias to 5′U and 5′A cannot be explained by nucleotide composition of the viral genomes which are only slightly enriched in A + U (54% in HvEV, 53% in BaYMV, 56% in JSBWMV; Supplementary Fig. S2d), and may therefore reflect preferential stabilization of viral siRNAs with 5′U and 5′A by AGO1- and AGO2-like proteins, respectively.

In summary, our in-depth sRNA analysis suggests that, for all the identified components of the barley virome, the processing of predominantly 21 and 22 nt viral siRNAs from dsRNA precursors representing the entire virus genome is likely mediated by barley orthologs of DCL4 and DCL2, respectively. The viral siRNA duplexes produced by both DCLs are presumably sorted by barley orthologs of AGO1 and AGO2 that preferentially stabilize siRNA strands with 5′U and 5′A, respectively. In the case of BaYMV, a 24 nt sRNA-generating activity also appears to have a contribution to DCL-based antiviral defence. Indeed, low abundance 24 nt sRNAs derived from both strands of BaYMV (Supplementary Dataset S3) accumulate at ca. 2-times higher levels than 23 or 25 nt sRNAs and may therefore represent siRNAs produced by DCL3 rather than non-DCL degradation products.

Previously, sRNA sequencing has been employed to investigate viral siRNA biogenesis for only one *Endornaviridae*, Helianthus annuus alphaendornavirus (HaEV)^17^. The size, polarity and hotspot profiles of HaEV-derived siRNAs in sunflower leaves^17^ resemble those of HvEV-derived siRNAs in barley leaves described above. The only difference is that neither 21 nor 22 nt siRNAs derived from HaEV exhibit any strong bias in relative frequencies of 5’-terminal nucleotides^17^, which may reflect differences in AGO family proteins or their expression in sunflower (*Asteraceae*) compared to barley (*Poaceae*). Consistent with our findings for the furovirus JSBWMV, sRNA sequencing analysis of a related furovirus Chinese wheat mosaic virus (CWMV) in wheat (*Poaceae*) leaves^18^ has revealed viral siRNA size, polarity and hotspot profiles strikingly similar to those of JSBWMV as well as a strong bias to 5′A and 5′U in both 21 and 22 nt siRNAs, albeit less pronounced than reported herein. Likewise, sRNA sequencing studies of the bymovirus wheat yellow mosaic virus (WYMV) in wheat leaves have revealed viral sRNA size, polarity and 5′-nt identity profiles resembling those we observed for BaYMV in barley leaves, including the presence of low-abundance 24 nt viral sRNAs accumulating at higher levels than 23 and 25 nt sRNAs^19,20^. Nonetheless, sRNA hotspot distribution profiles along RNA1 and RNA2 are quite different between BaYMV and WYMV, which may reflect differences in viral sequences as argued above or in host plant factors.

To our knowledge, viral siRNA biogenesis in barley was not investigated so far. For other *Poaceae*, besides the above-mentioned representatives of *Furovirus* and *Bymovirus* genera in wheat^18–20^, viral siRNA biogenesis has previously been studied for representatives of several other genera and families of RNA viruses, including *Potyvirus* (*Potyviridae*)^21–24^, *Poacevirus* (*Potyviridae*)^25^, *Tritimovirus* (*Potyviridae*)^25^, *Machlomovirus* (*Tombusviridae*)^23^, *Potexvirus* (*Alphaflexiviridae*)^26^, *Polerovirus* (*Luteoviridae*)^27,28^, *Tenuivirus* (*Phenuiviridae*)^29–31^, *Fijivirus* (*Reoviridae*)^32–35^, *Phytoreovirus* (*Reoviridae*)^36^ and *Cytorhabdovirus* (*Rhabdoviridae*)^37,38^, and for representarives of DNA viruses from genera *Tungrovirus* (*Caulimoviridae*)^39^ and *Mastrevirus* (*Geminiviridae*)^40,41^. Collectively and in line with our findings in barley, viral sRNA profiles reported in these studies involving *Poaceae* are consistent with the primary roles of DCL4 and DCL2 generating abundant 21-nt and 22-nt siRNAs from all types of RNA viruses and with an additional major contribution of DCL3 generating abundant 24-nt viral siRNAs from DNA viruses; the specialized roles of these DCLs in biogenesis of the respective viral siRNA size-classes and antiviral defense have previously been demonstrated in *Arabidopsis thaliana* (*Brassicaceae*) for some representatives of +ssRNA, dsDNA-RT and ssDNA viruses (reviewed in ref. ^2^). It remains to be investigated if low-abundance 24 nt sRNAs derived from the bymoviruses in barley and wheat and from other genera of RNA viruses in *Poaceae* and other plant families (see Table S1 in ref. ^2^) are indeed generated by DCL3 and whether those 24-nt sRNAs have any contribution to plant defense against RNA viruses. Barley and wheat seem to have evolved five DCLs with DCL3 having two forms, DCL3a and DCL3b^42^: it would be interesting to investigate their involvement in biogenesis of 24-nt siRNAs and defenses against DNA and RNA viruses.

### Concluding remarks

During field surveys leaves (or other organs) of plants are usually collected and desiccated using anhydrous calcium chloride (CaCl_2_) and further stored at room temperature or at 4 °C over CaCl_2_ (Luther Bos method^43^). This inexpensive technique can also be used for routine storage of viral inoculum for a wide range of viruses. Here we show that such dried leaf material can be used not only for virus identification and virome reconstruction but also for characterization of RNAi-based antiviral defence mechanisms generating viral siRNAs of distinct size-classes, 5′-nt identities and hotspot profiles. Our *de novo* reconstruction of the barley virome components by Illumina sRNA sequencing and bioinformatics generated contigs of sufficient length for the identification of three distinct viruses, including the alphaendornavirus of family *Endornaviridae* with a non-segmented dsRNA genome of 14.2 kb (the family recently re-classified from dsRNA to +ssRNA^44^), the bymovirus of +ssRNA family *Potyviridae* with two genomic RNAs of 7.6 and 3.5 kb, and the furovirus of +ssRNA family *Virgaviridae* with two genomic RNAs of 7.0 and 3.6 kb. Reliable reconstruction of their full genome sequences from viral siRNAs, however, necessitated the availability of reference sequences, either retrieved from the NCBI Genbank, or, for the novel furovirus with more divergent sequence, assembled from longer reads that we obtained through Illumina sequencing of dsRNA (also extracted from dried leaves). Such a limitation of sRNA sequencing approach has been reported in previous studies of land plant viruses, although in some cases deep-enough sequencing of plant small RNA-ome have allowed for *de novo* assembly of complete genomes of RNA and DNA viruses as well as viroids without the need for a reference sequence^2,4^. Our finding by in-depth bioinformatic analysis of dried leaf sRNA-ome that the majority of virus-derived sRNAs represent *bona fide* siRNAs, generated by distinct plant DCLs and stabilized by distinct plant AGOs, opens up the possibility to use dried plant tissues and organs from field surveys, herbaria and other sources to investigate RNAi responses to different viruses and virus-like agents in land plants. Such analysis may also extend a scope of research in paleovirology beyond addressing the evolution of viral genome sequences over time (see, e.g., ref. ^7^) and possibly uncover potential co-evolution (diversification, specialization, or change in relative activity) of the antiviral RNAi machinery components in viral hosts.

## Methods

### Field survey of barley viruses and preservation of collected leaf material

The samples of barley leaves with mosaic symptoms used in the present study were collected during field surveys in 2013–2015 in France and Germany as described previously^8^. The sample HYT-37 of *Hordeum vulgare* cv. Etincel was collected in France at the site Chouday in 2013, while the sample HYT-38 of *Hordeum vulgare* cv. Esterel was collected in Germany at the site Bornum in 2015. Additional samples of dried barley leaves of the corresponding cultivars used in this study for dsRNA sequencing and RNA blot hybridization analyses were collected at the same sites (Chouday and Bornum) and years (2013 and 2015)^8^. The samples were dried at room temperature and kept stored over anhydrous CaCl_2_ at room temperature until used in August 2016 for the present study. High-throughput dsRNA sequencing was performed on these samples as described previously^8,45^. As a control for RNA blot hybridization analysis of the dried leaf samples (Supplementary Fig. S1), fresh leaves of healthy barley seedlings (*Hordeum vulgare* cv. Etincel) grown in a phytochamber at the BGPI (Montpellier) were taken directly for total RNA extraction.

### Total RNA extraction from dried leaves for sRNA sequencing

Total RNA from both the dried barley leaves and the fresh leaves was extracted using a CTAB-LiCl method^46^ with few modifications as follows. One gram of dried (or fresh) leaf tissue was ground in liquid nitrogen. 10 ml isolation buffer [300 mM Tris-HCl pH 8.0, 25 mM EDTA, 2 M NaCl, 2% weight/volume (w/v) CTAB, 2% w/v PVP, and 2% v/v beta-mercaptoethanol (added just before use)] was pre-warmed at 65 °C and added to the fine tissue powder. The mixture was incubated at 65 °C for 10 min with shaking every 2 min, followed by addition of 10 ml chloroform-isoamyl alcohol (Chl/Iaa, 24:1, v/v), vortexing and centrifugation at 5000 g for 10 min at 4 °C. The aqueous layer was transferred to a new tube and mixed with an equal volume of Chl/Iaa, followed by centrifugation at 10000 g for 10 min at 4 °C. The aqueous supernatant was transferred to a new tube and mixed with 1/10 volume of 3 M sodium acetate (NaOAc, pH 5.2) and 0.6 volume of isopropanol, and the mixture was incubated at −20 °C for 1 hr. Precipitated material was pelleted by centrifugation at 20000 g for 20 min at 4 °C. The pellet was dissolved in 1 ml RNase-free water and transferred to a microcentrifuge tube. The RNA was selectively precipitated by addition of 0.3 volume of 10 M LiCl, careful mixing and overnight incubation at 4 °C, followed by centrifugation at 20000 g for 30 min at 4 °C. The RNA pellet was resuspended in 0.1 ml of RNase-free water, and 0.1 volume of 3 M NaOAc (pH 5.2) and 2 volumes of cold absolute ethanol were subsequently added, and the mixture was immediately centrifuged at 20000 g for 20 min at 4 °C. The RNA pellet was washed with ice-cold 70% ethanol, allowed to dry, and dissolved in a volume of 30 to 50 µl RNAse-free water.

The integrity of high and low molecular weight RNA was evaluated by electrophoresis on respectively a 1.2% agarose-formaldehyde gel, followed by EtBr staining, and a 15% polyacrylamide-urea gel, followed by blot hybridization with plant miR160- and BaYMV-specific probes (see Supplementary Fig. S1), as described in detail by Malpica-López and co-workers^47^. Before preparation of sRNA libraries for Illumina sequencing, the total RNA samples HYT-37 and HYT-38 were subjected to additional quality control by capillary electrophoresis on LabChip GX (Perkin Elmer) (see Supplementary Fig. S2). The above analyses showed signs of partial degradation of high molecular weight RNA in the two samples, but both samples passed the quality control for sRNA library preparation.

### Illumina sequencing and bioinformatics analysis of sRNAs

The 18–30 nt fractions of the total RNA samples were sequenced at Fasteris (www.fasteris.com) using TruSeq® small RNA Library Prep Kit (Illumina) for cDNA library preparation and multiplexing the two barley libraries HYT-37 and HYT-38 with 10 non-barley cDNA libraries in one lane of HiSeq 4000. After demultiplexing and adapter trimming, sRNA reads were sorted in separated fastq files and counted. For the barley libraries, a total of 20′128′818 (HYT-37) and 20′157′823 (HYT-38) reads passed the Illumina quality filter, of which 97.99% and 97.93%, respectively, had base quality scores equal to or more than Q30. These numbers and scores were comparable to those of the non-barley libraries. Both sRNA read datasets had a broad size distribution from 16 to 32 nts, indicating substantial contribution of random non-RNAi degradation products to the functional sRNA population normally dominated by 21–22 nt and 24 nt size-classes in land plants^2^. For further analysis, a size range from 20 to 25 nts was selected with a total number of 8′782′562 (HYT-37) and 7′725′316 (HYT-38) (Supplementary Dataset S2).

For virus identification and virome reconstruction the redundant and non-redundant sRNA reads from 20 to 25 nts in length, were assembled into contigs using Velvet 1.2.10^48^ (https://www.ebi.ac.uk/~zerbino/velvet/), followed by Oases 0.2.09^49^ (https://www.ebi.ac.uk/~zerbino/oases/). The Oases contigs obtained with different k-mers values (13, 15, 17, 19, 21) were further assembled using the Seqman module of Lasergene DNASTAR 12.0.0 Core Suite (DNAStar, Madison, WI). The Seqman contigs were blasted on the NCBI nucleotide BLAST database (blastn) and the most closely related reference sequences of the viral species were taken for further analysis. Likewise, reference sequences for isolates of the corresponding viruses identified during the barley field surveys and reconstructed by dsRNA sequencing as described above were selected. Oases and Seqman contigs were mapped to the selected viral reference sequences using BWA 0.7.12^50^, and visualized by IGV^51^ (http://software.broadinstitute.org/software/igv/). The consensus sequences were downloaded from IGV and curated manually. The consensus sequences were further corrected by mapping the redundant 20–25 nt sRNA reads and analysing the resulting BAM files by MISIS-2^52^ (https://www.fasteris.com/apps/) to visualize sRNA coverage, identify SNPs and indels, and download the refined consensus sequences as described previously^52^. The redundant 20–25 nt sRNA datasets were then mapped using BWA to the reconstructed viral genome consensus sequences and the genome sequence of *Hordeum vulgare* (assembly version v2: ftp://ftp.ensemblgenomes.org/pub/plants/release-42/fasta/hordeum_vulgare/dna/) with zero mismatches and with up to 2 mismatches. The resulting mapping data were analyzed using MISIS-2 and in-house scripts to sort and count the viral and host plant sRNAs by size (20, 21, 22, 23, 24, 25 nt, total 20–25 nt), polarity (forward, reverse, total) and 5′-nucleotide identity (5′A, 5′C, 5′G, 5′U) and create count tables (Supplementary Dataset S2) and viral sRNA single-nucleotide resolution maps (Supplementary Dataset S3).

## Supplementary information

Supplementary information 1

Supplementary information 2

Supplementary information 3

Supplementary information 4

## Data Availability

Viral genome sequences obtained in this study were deposited in NCBI GenBank with the accession numbers MN107377-MN107383 (assigned on 27/06/2019) and MN123252-MN123254 (assigned on 02/07/2019) (see Supplementary Dataset S1 for the submitted sequences).
