# Supplementary material for: Plant virome reconstruction and antiviral RNAi characterization by deep sequencing of small RNAs from dried leaves
Source: Sci Rep. 2019 Dec 17;9:19268. doi: 10.1038/s41598-019-55547-3 (PMC6917709; doi:10.1038/s41598-019-55547-3)

Plant virome reconstruction and antiviral RNAi characterization by deep sequencing of small RNAs from dried leaves

Victor Golyaev, Thierry Candresse, Frank Rabenstein, Mikhail M. Pooggin

Supplementary Figure S1. RNA blot hybridization analysis of total RNA extracted from dried barley leaves collected during field surveys and from fresh leaves of barley seedlings grown in a phytochamber. The total RNA was extracted from both dried (lanes 2, 3, 5, 6) and fresh (lanes 1, 4) leaves using the same CTAB-LiCl protocol, separated on 15% polyacrylamide gel, stained with ethidium bromide (EtBr) (**a**), blotted onto a nylon N+ membrane, UV-crosslinked to the membrane and then successively hybridized with P32-labelled DNA oligonucleotide probes specific to the evolutionarily conserved plant miRNA (21-nt miR160a; the probe miR160a_as 5'-TGGCATACAGGGAGCCAGGCA) and BaYMV-derived antisense siRNAs (a mixture of oligos BaymvRNA2_889s 5'-GGTGATGGTGAGCACTACACAAATTGGAACTCT and BaymvRNA2_1405s 5'-TTTGTTTCAGAGTCATCAAATGGTGAGTACAGA). After the 1st hybridization the membrane was washed and exposed on a phosphor screen for 19 hours and scanned (**b**), while after the 2nd hybridization – exposed for 3 days and scanned (**c**). Positions of 21-nt plant miR160 and 21-, 22- and 24-nt viral siRNAs are indicated. Lanes 2 and 3, dried leaf samples HYT37 (field, France, 2013) and HYT38 (field, Germany, 2015), respectively; lanes 5 and 6, dried leaf samples from the field (France, 2013); lanes 1 and 4, samples of fresh leaves of barley seedlings grown in a phytochamber.

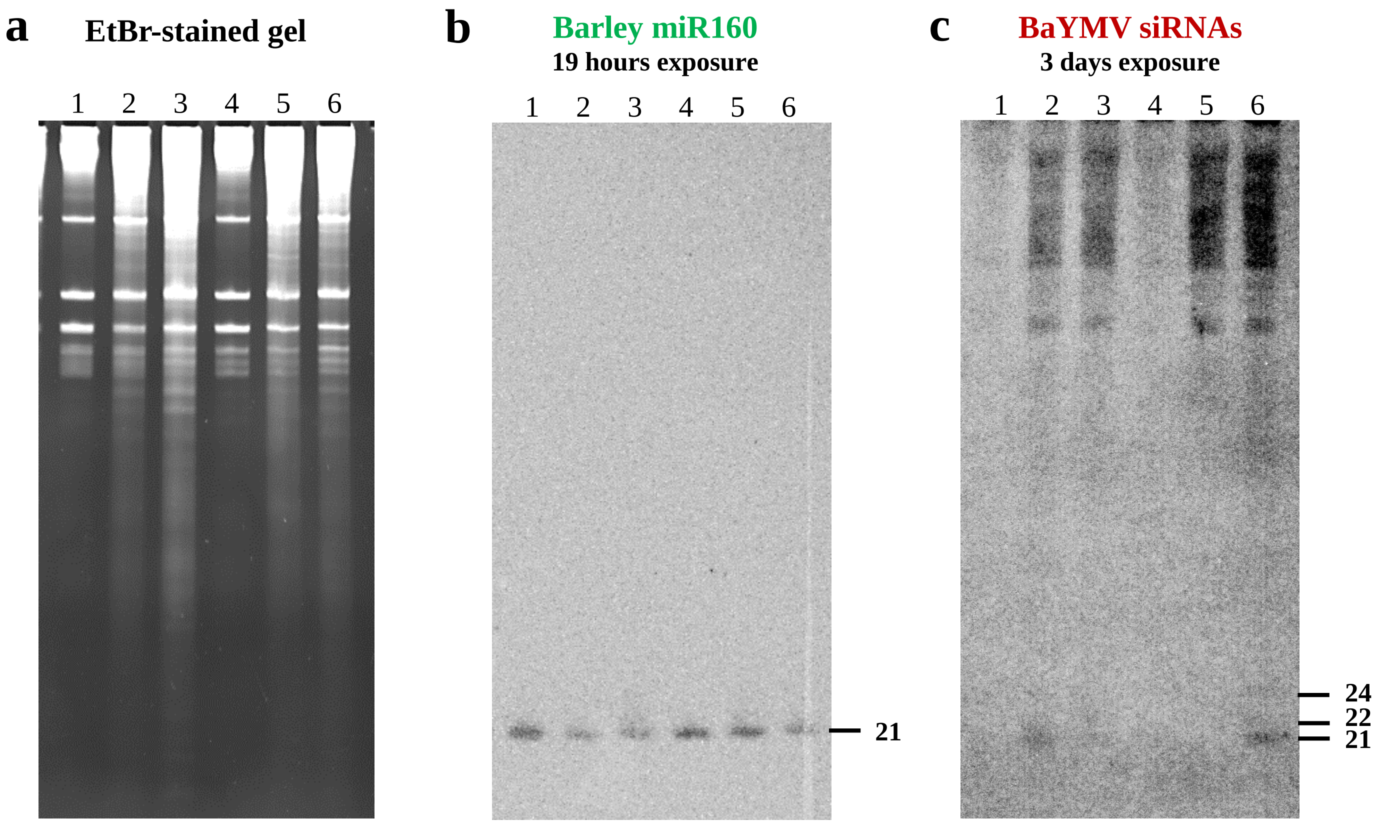

Plant virome reconstruction and antiviral RNAi characterization by deep sequencing of small RNAs from dried leaves

Victor Golyaev, Thierry Candresse, Frank Rabenstein, Mikhail M. Pooggin

Supplementary Figure S2. Quality control of total RNA extracted from dried barley leaves. The total RNA samples HYT-37 and HYT-38 were analysed by capillary electrophoresis on LabChip GX (Perkin Elmer), with LabChip GX Software v. 4.2.1745.0.

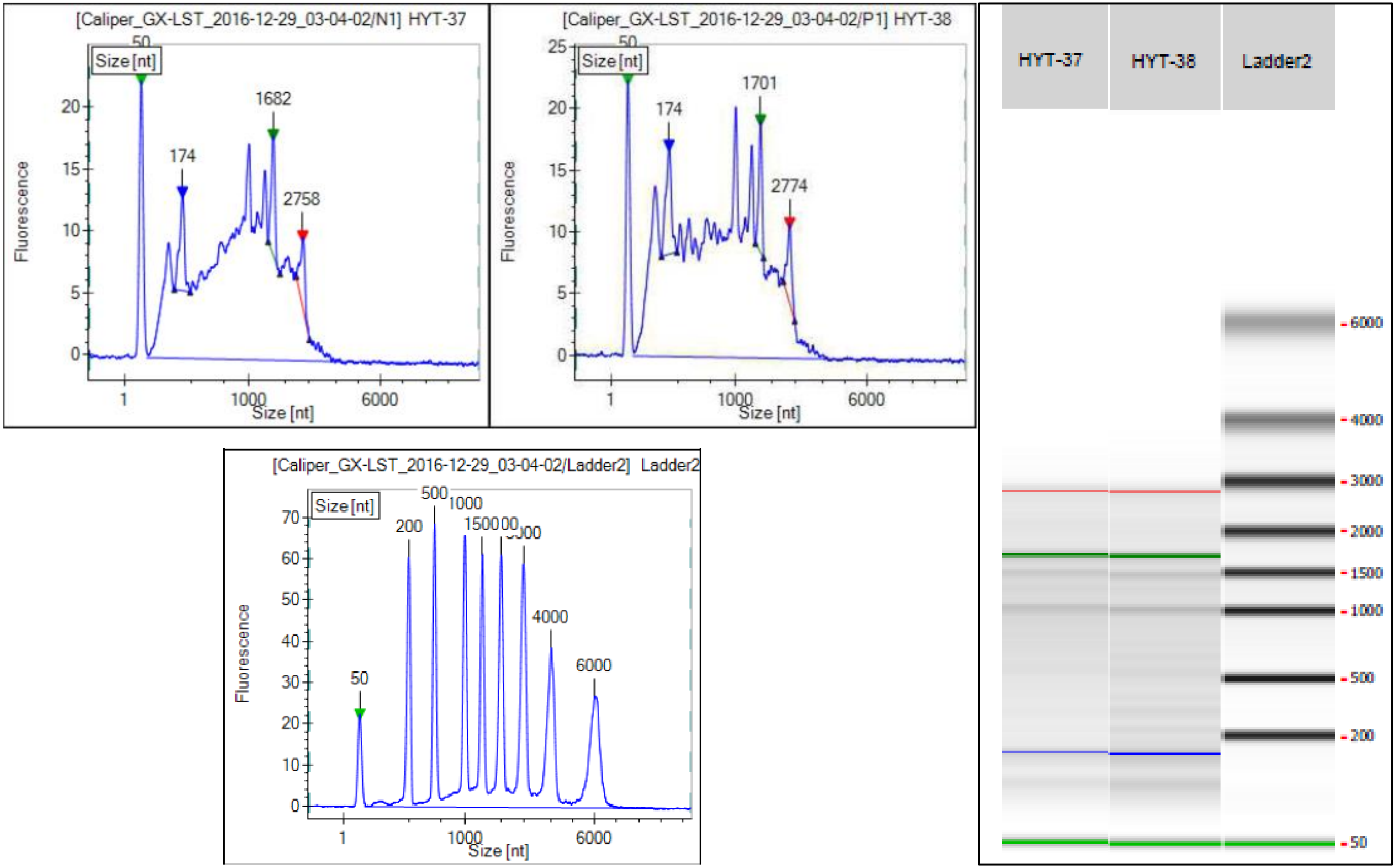

Supplement: Supplementary file 1 — Supplementary information 1 [file 41598_2019_55547_MOESM1_ESM.pdf]
