# Supplementary material for: Plant virome reconstruction and antiviral RNAi characterization by deep sequencing of small RNAs from dried leaves
Source: Sci Rep. 2019 Dec 17;9:19268. doi: 10.1038/s41598-019-55547-3 (PMC6917709; doi:10.1038/s41598-019-55547-3)
Supplement: Supplementary file 2 — Supplementary information 2 [file 41598_2019_55547_MOESM2_ESM.docx]

**Plant virome reconstruction and antiviral RNAi characterization by deep sequencing of small RNAs from dried leaves**

**Victor Golyaev**, **Thierry Candresse**, **Frank Rabenstein**, **Mikhail M. Pooggin**

**Supplementary Dataset S1. Reference sequences used for bioinformatics analyses**

Viral genome sequences used for reference-based small RNA assembly and virus reconstruction:

>HvEV_NC_028949

>BaYMV_RNA1_KX117192

>BaYMV_RNA2_MN107379

>JSBWMV_RNA1_MN123252

>JSBWMV_RNA2_KU236377

Reconstructed sequences of the viral genomes used for phylogenetic analysis and for mapping and counting of viral small RNAs:

>HvEV_HYT37_MN107382

>HvEV_HYT38_MN107383

>BaYMV_RNA1_HYT37_MN107377

>BaYMV_RNA1_HYT38_MN107378

>BaYMV_RNA2_HYT37_MN107381

>BaYMV_RNA2_HYT38_MN107380

>JSBWMV_RNA1_HYT38_MN123253

>JSBWMV_RNA2_HYT38_MN123254

*Note that the reference sequence nucleotides that are not covered by sRNA reads in HvEV_HYT37_MN107382, HvEV_HYT38_MN107383, JSBWMV_RNA1_HYT38_MN123253, and JSBWMV_RNA2_HYT38_MN123254 are shown in smaller case*

>HvEV_NC_028949

GTCAACCAACCATGTACAACCGAACAGTACATGAGACAGGCAATTCGTCACCAAAACAAAAAGAGGTCGAGTGGCTTAAAGCCACTCGACCTCTAAAAAGAGACGTGAATAAACGATGGAAGGCAAAGCTGGGAAAACCAAAATTGCGACAAAAACCATTAAGATTGAAGCGTCTCAAAGGGCAAGTGGTGAACCGATCCAACAACATGAACAGCCAATACGACAACATGAAAAATTATTACAATCAAATGACAAAAGTACGCATATTAGGTAGAACCATGGTACCCTGTGCATTGGCTTTGCAATTTCCCATGTCCGAACCATTGGTCAACAATTACATGGAAGCAAGTTACATTGCCACATATAAACTGATGTACAAAAACAAAGAAAGTTACGAAGGGTTGCACCCGCATGAACTGGCTGCATTTATAAAAGGTGTTAGATTAAATGTGGAAGACAGTTTCAACCATGCTGGTAACAAATTATTCTATGACTCTGTCGAACAAGAGGTGTCAACTCACACTATAGACACACCAAACGATCCAATATTGTGGCAGGCAATTAGAGAGATCGAAGCCAAATACGGAGGGGCCATTGGCATGCAATACCAAGCTCTGCGCAACTGTTGGACAAGAGTTCCGCACCTGGCCAAGGTTGGTGGTAGACACCAACTGTTGATGGTAGATGCAGAACCATGCGGACACAGGCGTTGCGCGGAGTGCGGAATCATGAATGGCTACTGGATCCCTGGCGAGGATGGATTATCCAATGCACACTGCGGGGCTTGCGGCACTTCATTAGATTTCGAGAGACAAACTCCAGAGCCTCTCGACACACCGCCAACCGAAGATTTCCAGCAAATCAACCAACCCATGGGTGCCTTCGAAATGGCGCAGATGGAAATCATTGAACAGTTAGACGGAGTAGGAGATTTGGAGGGGGTAGCACTGAAGCAAGCAATTGAAAATGATGAAAGACTGATAAGAGCGATGATATCAAGGGCGAACGTCAAGTTGAGTTTTGTTGATCCGGGCATGACACCTGAACAACGTAAATTGGTAGGTTCAAGCTTCAAAAATTTTATCTTGATAGACAAGGTAGGGCCAATAAACGAACATGCCATGATTGCAGCAGAACGTCAGCTTATGGCACAAGTTATGTATGACATGGCAGCCGGAGGTCAACCCATAGTGGACATTGGAGGGCCTAGCCCCGGTTGGACGGCAGTCAAATCGTACCAGACGGTGAGCCCCATCCTATCAATCAAAGACATAAAACGGTTCAACAATTACGACAAGATGCCGGTGCACTGCACACACAAACTGAAAGAATGTAGTTGTCACACGGGACAAGCACCATTAATTATGAGTGTTGACAGCTTGTATGATATATCACCAAAAGATGTCATGAAATTTATGTTACTGGCCAAATCAAATTACATAGTTTACTGCCTGAGCACAGCCACACTGGACCACTCCACAAACAAGGGTGTGCTGCCTTGGGATCAAGGGCTGTGGATAAAGCATCAAGGCAAGTTGGTGACAACATTCAATGGGTCATCTTCAACGTATGAAAACAATTGGGCCCAAACCTTATGGTGGACCAAGGCAGATGTGATAATAGTGGGGGACCATAGCTTATGCATACAAACCCTAAAAGTGTGCGGAAACCACATTCTGCGTGTAGCCACAATATGCAACGATTACAAAGCACAGTTTTTCTCACTAAATAAACCCGTTATAATGAGCTCATCTTCCAGTGTATGCATGCCAATACCTGTATGGAAAAATCCAATCAGTCACGTTGTCAAGAATGTGGTGGAGTGGAGGACGGTGGCCGTGGAAAAAGAATTTCTTAAATTGCTGTTAGTAAGAGCGAGCGCAGGTCAATGTTCTTATGATGATCTGGTCAAATATGCAATAGGGCTGGGTTATAGCAAGTACACACTAAAAGATAAGGTAATATCTATGAATTATATAACTGCGGATATGGCGTTAAACCATGCGTTAATAGCAAAGGCCATCACTGATAGAAAGCTGCTGGAATCAACTTACATCATGGCACACGACAAACAATTTTACGATTGGCATAACATGAGCCAGACGGACGTGTTACAAACCATGGGCACGTTAATCTTCGAAATTACAAAACACTTGCTCTCAAAGTGGTCTGCATCCGACGACAAGGTTCAGTTTGTGTTTAACAAAGTAGTTGACGGTATATCTGAATGGATAGCTGATCCAGCATGGTACAATCTGGTGGAGGCGATCACATTCGATGACATTACAAAAATGAACATCATTAGAGTGAACACATCCAGTCCGGACACAAACGAAGCCATCATATGTGACCATCACAGCGACCATTGCAGTCACACTGTACAACTGTCACCACATTATTGTCAATGTTGTGGGTTGTACCCCGTCGTGGCCGGTGAAAACCGTTGTGGCTGTTGCAAAAGCGATCACTGCCACCACAAGTGTCACCACAAGTGCACAAACAGATCGGAGCATCTCGCGGGCACAAAAACAACACCTCGGGAATGCACCTGCTGTGGTGTCGAGTACGATACATATGTGTGCGTTGTCTGCAACAGACCTGATGGGTACAGTGTCGTAACGGACGCAGAAGTGAGGAAAAGAGTGCATGAGGCAGAGTTGGCTGACAAGCAAAGAAAATCCAACTTAAAACAGTCTAAAACACAACAGTCACTCGAACAAGAGGGTGACACGGGCAGAGGCAAATCAGCAACCAAAACTCACAGCGCTGATCCCGACCCAAGTACCGCCAACGCGACCAACGTGGCGCATGCAGCACCCATCACGGAGCCAACAGCACCACTTGCGGACCCATCGAGTGACCAGCGTAACACCGCCACACCAACCGTGTACGATTTACTGAAGCTGCACATGGACGACTTAATCGAAAGCTCCACACCAAGGCTGTATTCAGATGTGTTGAGTTCATCCACCGCGGAGTCAACACCCCAGGTTAACGAACAGCCGACCAGGCAAGGCCGCGACCCACCAGCAGTTGAAACTCGGGAGCCGAGCATTAATGTTGTGAAACCAATGATGACAATGAGTATCAATCACCCAACCGACATAACAACCATCACGGACATCGAAGCGGGCAATGTCGAGGGACGCCTTTTCCTTGAACTGGTTAACCTCACAAGCTTGGAATTTGCTAAAATCATAGCTGACAATGAGCGAGGATGGGCATGCGTTGAAAGTGAAACAGAAGTGAATCATACTAAAGGTTTATCTCAAATCAAATATGTTGGGAGGAATATTACGGTCACAACCCCAAATAATTTTGAGATAGTTGAAATCACTGACGTTAGTGGCCAAGGCAACCAATGTGGTTATAACGTCATCAATGACACCGTGGGAGTGGACAATGACGTGTGGACTTCAATTATCAGTTCACGACAAATGTACAGCGACATGGAGGTGCAGCAATACTGCACAATGATGAACCTAAATTTGTTGCTATTGGGTAGGACAAACACCTTTTTCCATCGCCCAAACACAGCATCAGACACATTCATTGTCATGCGCCATTGCAGCTTGGACAGTCCAGAAGGCACTCCAAAAGAGTTATGGTTCAAACATTGGCAGCGCGCGAATGTAAAAGTTAAGTTTTCAAGACCATGGGTCCCGATTTTCTACCCATGGTTGGAAAGGGCTGCCTGGGACAGGTTCATGCTTGACAACAGAATTAACGAAGACTTTTGGCAATTAGCAACCGAGCAAAGACTACAATTGATGGTTGAATTTTACACAAACAATTTTGTGTGTAGCAACCTAAAAGAAGGATATTTGGCCATGCTAACAATGGATCTAAAGGCATGCCCGCCCGTCATTTACAATAACTCATTGATGATGGACCAACCAGACACACACCTAAAAGGCTGTGTGGTGAGCAAACAAGGTGCGGCAAAGTTACCATACGCTCTGACGCAGCACCAGTCCATAGAGCAAGACTCAATAATGGGTGCCCCCATCAATCAGCAACCACCAACCAATGAGTCAGAACTGGAAGTGTGGTTGAACAATGAAATCAAGCTAGAGTGCATTGATTATGCATACAATATGGAGCACATGATGTTATTGGAAGAAGAGATGCATTTTAAAGAGTGGCAGACAGTCAACGTAAAGTTCGTAGGTCAGCGTCTACGATTTTCGAAAGGCTTGTTCAAAAAAATTAAAACAGGTGACATAATCATGATTAAAGTGGCAGGTTACAAAATGACAACAACGGTGACCATCAGCATGCGCCAAGTGCTTTGCGACAAGCCGTACACCGGGCCGACAATTGACCAGGCCCTGGTGGGCGTGAACAAACAAAGCCATGCCTCCAAAATGGCACAAGTGATGACGTTGTTGAGCAAAACCCTGGACGCCAAGCACATATGGGATAAATTGAAATCAGCAACAGTGATCAAGGGCCCAGGTGGCAGCGGAAAAACCACCGGGCTGCTTCGTGAAGCACACAAAGGCAGCATAATTCTAACTAAAACAACCATGGCAAAAACCAACATATTGAAACACAACCCACCCTGCCCCGTCATGACCCTAGAGGCATACCACAAAAAAGATGACCAACATACAAAAATACTCATAGACGAGGCCGGCATGTTCTCACTGCTAGATTTTGCCACCCTAAGAATGCAAACAAACACACAAATAATTATGAGCGGAGATTTATTCCAAATAGGGCAACTAATTACCCACGTACACCCAGGAATTAGGGATTACCAACCTTGTATCAATTTTCCGGCAAACGTGACCACACTAAATTCAACATACAGGTACGGCAAGTCTGTGTGCGATGTGTTGCACACAGCTGGCATGAACGTGACGTCGTTGGCACCATATGACACAACCATAACTCTATTAGACATTAAGGAAATCAGATTGGGAACGATTAAAGAGTTAGTCAAAAAGTACACCCCGGACGCAGTATTGACATTTTACCAATCACAAAAAAATCTGGTGGAATCATGGCAGCTGGACCCAGATACCCACACCGTACACGAATTTCAAGGAAACGAATGTGACACAGTTATGGTGTTGCAAGGCCCACAAGCCACAGCAACCACAGGAATATGGAATAAACCAGAATATTGTATTTCGGCACTGACTAGGGCCAGGAAGCACGTGGTGTGGGTCACTTTTAATGCAACCACGGGCATGAACATGGTACAACGATGTGGTTTAAGCCCAGAACTGAGGGCCACCCACAACACCACATCATTGACCCAAACTTGGGCGGACATGGTGGAAGAAGACCAAACCAAAGAGGCAATGCGGGGCGGTGGGCTCAATACTGACGGGCTAACCATTTACGTTGGCCGCAACACCAACCACGTCACAGATTTAAAGTCCATGACCACCATTAAAAACAAAACAGTAGTGGGGGACGAAGAACTGTTTGTAGTGTTGCTGCAGAGCGACTGGTTAATGGGCATGGTTGACGAGAACAAATATTATCTGGTGGCCGGCCAAGAACTAGCCACCATGAAAGAGCCAAAAAATGTGACCGATTGGAGGCGCTTGCTGTCTGACCTCACAATTAGCGAGCAGGCACCATCCAGATTGCTGCAAGCAGCCAGCAATTTCTTCTCCGCAACGATGATTTTCAAAAACAACATGGTGCATCTCTCTGAAGACAGGGCCGTGTTGTACGTGCCAGACACACAAGCAAAACTAACCGAGTTAGAACAAAAAATAAGGGCTAAAACCAAGCTAGTGCAGATAACAAGAACCCACAAAGAATTGAAACTCCACTTTCTGCTCACCACGGTGGGGACCATAACAATTACTGGGCGACACAGAATAGAAGTGTTTGGTAATAGGGCGCTCAACTACTTGACAAGTCATGTTGATCAAGTTAATGACGCTCAATTCAAGGCATGGGTTGCACATTTGATAGACAACTTCAGATGCACAGCGTGCGCAGCAGAGATGTACATTGATCCAGTGGCATTAACTGAGCACTTGTCAAATCCAAACAGTGATACACAATGGACCGGGTGTAGCAGGTGGAGCTGCGAAAATTTCAAAGCCCCACCAAGCTCCGAAGAAAACTCAATCGAAGACGTGATGGACACAGAATCGTCTTCGTCAGAAGAGGAACCGGACAAGGAACCTGCCAATGGCGGCACTTTGCTAGGCGACGAAGCAGCCACAGGCCAAGAGTGTGAGCATGAATCAGACGAAGAATACTATGACACGATTGACACCATTCACGTAAACCCCACGACCGTCTGCACCACACCGCACAACAGAAGTGGCCAAGACGACGTTAACAACGAAATTGTTCAACTAATGTCAATAGCACAGCCAAGTGGAGGGCAGACCGCAGAAAACCAAACAGCAACAACTTCCCGCACACATCAATCATCACCACCACCCAACCCCCAACAACAGGAGCGCACATTAAATAATTTGGTGGTGTCAACCTTGCATAACAACCAACCCAGACGCACATCAAGAGAGAGGGTGATGCACCTCGTCGAACAGTACGCGCAACACACCACCGGGTGGATTATCTTTCCGCGATGCATCAGCATCAGGCATATGCAAACAGCTCTATTACATGGCTGCCCAATGCACTGCAGATGGACTCTGCAACGAAACGAAATGTGCAACATTTTTACCCTCATGGCCGGGCTGACTAGGGTGCTCACCATTGCTTTTAATAGAGCGGGCAACGTGGTTGGTCCCATGGACGGAAGTGTAGCGGACGATATGTACATCACATTGGTAACACATGCCGTGGACCAATGGCGGTTGGTGGCTTCGGGTCAAATGGAGCTGGATGAAGATTTAGCATTGGAGGGGTTGAAATTCATTAACCAATCACGATTGTCACCGATCCATGTGCATAGGCATGACACAATTGTCATTGATGATACCGAATGGCCGGCATGGATCGGCGCCTCCGTGAAGGAATATGAGCCATTTATGACCAGACCAAGATACCAACAATCCGTGACACACCACGCAATAAAGACCATGCTACCAGAATTGCCCACTGAAGTGTGCGGGTGCGAAACCAAAATTCTTGACGAAAACAAGATATGCGCCACATACCCCTGGTTAGACACGTATCTAATGACACCTGATGAACTTGCAAGTTGCATTAGTTGGATCCAATTCGTGCAGTATCGACAAATTGGGGGTCTGAAAACCCACACAGTTATAGATGGCACAGTAGTGGATAGCACAACCTTCGGGGGCTGTGCACTGTGCGCAGGGCTGAAGATGACAATAAATGAGGAAATTACAATGTTAATCAATAGTAGATCCGGCCCCGAACCCGGAACCATGTGGGTCAACATCAATCATGTAATACAATATCCACATGTGATCTCAGAGCTGTGCAAAATTCTAGAAATGCCTGGCATGATCATGCATTACCCAGATTTGGAGCTGTATTGGTCCGTCAGGCAACTGACCGAAATGGACGGCATGATACAAGACTTCGGCCAAGCCATGTCGTTAATTGAGCGACTCAGAGCAGGAGTTGATTGGGTGATAGAAAGGGTCACCAGCTTCAGTTTACAATGGAAGTGTGACCAATATAACACAGTCAATTCGGAGTACGTGACCATGCTGGGCAAAGCACTGTTGGACGCAGGGGTTGATCCATCATTACACTGTCATGCAATCACTCATACAGGCACACTATTGGTGGGGCTGCCAACAAGATGGTCAGCCTCGGGCCAAACCATTGTTATATCAAAATTCAAGGGCAGATTTCACTGCACCAGTCACGCACTGTCCATCACGCAGCGATGGGAGTGGCAAAGCGCTAGTGACATGCTAGTGGAGGCAGTAAAACACCGCATGCTGATACTCTTTGCAAACAAATTGAGTGGCCGCAGAGAGTTGGTTCGGTCCATTTTGGAAGATTCTGTAGCCATCAACCTGGAAGAAGTAAGAAGGGCCATGGGAGCAGTTAAGCTCAACTTGAGTTTGGATTACCACAGGGAGCACAACACCGGGGTGAACGCTATGTTGAACGATCGCAAGAACAAACTGGTGCAGGGACAACTCGTGGGCCCCCACCAGGCAATGTACTTGTCACCAAATCAAATCCGGATGTTTGGCAGAGATATTAATAGAACATGTTGCACGCACAACTTGACAGAACACGGCACCTGGCTACCCGACGAAGGGTTATTTGCCACAGCGGAACAGGTGGCAGCGAAAATAATATCTGGCTGGTGTGGGAAAAACAAATTGTGCACATACTCGGGTACATATAGCTATATACCTTTAGTGAACTCTAGCTGGAACATGAAAAGTGTAAAACCATTACGTAGTACACCGCTATACAGTGCGTGTGCACTAGCCATGAATGGGGCTTTAAACAATTTGATCACATCTATTAAAAACTTGAAAGATAAAGCAGAAACCGTGACCGGGTTTGCATTGAGTGATAATGAAAAAGCCCTGATAGCCATGTGTGACAAGGTTGCAACCACAAAAACAAACCCTTGGTGCAGCAGCGAGTCAGTCAGTGATGGCGACAAAGCCATGTTGGGAAGTGAGTATTTAGGCCTGGACTGGCATTCGTTTTGTGAGCAACTGGTAAAAACCAGTGCAAGGGAAACATTCATTTTGGTCCCCGAGCAAGACAATCACAGCGGTGATTACTACAACATCCATGACAGGCAAGAGTATTTTGAATGTCAAGGGGTTGCGAGCACCACATTAATAACCCTCAACCCCACCACATGGAGCAACCTGCTTATCAAGCACAGCATGAGGTACAACAACAAAATAATACACGCAGAACAGCTAATGTCAATATTAGGTTTGCGGCTTGTCAGCATAGTGGTGACCTCCCCAACAGTGTCAGTGCCTGGCCATTATTCACCATGTATACCAAACAACAGCAATCAAGTGGTCTACAAAATCAATACCCCACAATTGTCATGGAATACATCCGTTTTCAGCCACCACAAGACAACAGTAAAGGTGGACACCAAAATGTACCGCCATCTGAGCTTTAGAGTTGTGTCTGATACGTGCAGCTTCGAAGATTTATTGTCCTACGCCAGAACGTACATGCAAACTACCACCTACACTCAGACTGGATACTACAGGGAGAATTTGGACCAAATCAATGATTTGCATCTCATTTGCGCGTGCGTTTATCATGAATATCAGCAGAAGAAGACAAGCTGGTGCACTCTAGCCGCGAAACATTGTGATTTACCAACCATGGATGTGGGCCGGGTAATGACACATTTGAACACCAATTTGACCCATGCCGCATTTAAGCTGATGCAAGCATGGAAGTTGAATGACGGAACTTTGCATGACATTAAATTGGCTTCCAAAGACACGATAGGACCCGAGATGACAGACATAATCAAAAATCTTGCCACAATGAACATTGAGAAAAAAGAGGTGAGCCGGCAAATCATGGTACAAAACGGAGATCGCGCTTTGCCAGACATCACAACAAATTGCCCATGTGAGAGGCAAATGACTAGATCCAACGATGTAGTAATGCCAATACTGGGCGGATTGGTCAATGACGGATCATTGCTGACAGAGTTTTTGGCGGAAGAACTACGATCAAACTCAAGTTATTGGTGCTTAATACAATCCAGCACTGACGGCACTAACACGGCCGTACAGTTAGGGGACGGAGTTAGGCGTTCCCACAATTTGTCAAGTGTAAATCTCAAAAAGAAACGTAGGTGCAAACACCCCGGCGGTGAAATAAACCTTACAGAGAAGTACCTACAGCAAAGGATGGAAATCACTGCCAATATGAGCAGCACGAACGACACTCAATCACGTGAGTCAACTGGCCCACCTATCACAGGCACATACAGCACACCAACAATGAACCGATGCAAGACCAGTGGCCATGAACCATCAGTCGGCAACTTACAGCAACCTGCAGCGAGTAATAATGAGGACGAGGGCACCACCGAAAGTACCTGGAGACTAATGGAACTATCGCATGTCATCATTAAACGAGTGTCCCAAGCCGTCACCAGCAGGGTGGGTGAATGCCGGCAAAATAATTGTAAGGCCAGATCCAAGGAAATCAAACGACTGTTGAAAGATTTAGCTTCAGCAGTGTGCCACAAGTGCAATACCTGGGCAGAGGAGCATTTTGCGACCAGCAAGGGCCACGAAGAGTCTGGAGCCACGGAACCGCTGAATAGAACCACAACACCCGAGCCAACACCAGGCAGCACAAGACCCACAGGCGGAGAAAGAGTGTGGGTGTTGATGTCCCCAACACAACGCGTTGGCATCGTAATGCAAGAATTAAACCAGGCCCACCCACATGCCGAGCTCTCACAATTGAGAACTGTGACTAGTGACAAGAGTCATGACAACGACAACATGGACAGTGCGATACGCGCAAAGGCCGACCAATTGACCGAGCTAATGGAGAGTGGGTTTTTATCAGAATTTGACAGGGCGAACACCATTAGCTCGTGCGACGATAACATCTTGGAATGTGAAGAGCCCACGACGTTTGTTAAAAAGCAGTGGCAGCAGTGCAATGTGGACTGTGTGATCACAGAAGAGTCCCATACCCAAAGCGACCTGGAGGAACCACAGTTGACCACCGGAGCCTCCGAGCCAACGTCGAGCGAGGCACAATCACATAATGACAAAAATGCATACGCACGCATGTGCGACAAGACCCACAAGCTGTTGTGTGATACAACGGCTAACAATGCCGGGTTGATGGAAGATTACATTGAATCAACAACACTAGAGTGGTTGACTGAAGAAGCAAGGCAAGCCAATCGTACGTGTTGGTGCCATGTGACCATGCCCGAATGTTTACAAGAGTTAGTGGGAATTGATGCATGCCATGTCGTTGTCAAACCATATGCTCACATTACAGGTGTGGCCACCAGATCCAAAGAAATATACAATCCCAACACAGAAGGAAACTGCGTGGTAAATAGCCTGAACTATCTTGAGCAACGCCATCCCAGAGTAGACAAAGTGATGGAAAGGGCTTGCGGTGTGTACGGGAAGACCATGTGGTTGACGGAGTCCGACGCAGCAGCCATCAGCTGGTTGTGCAACCTAGAAACCCGTTATAAACACAACCAAACTGTGCATGAATTGGCTACTGGGACAAGCGCTGCCCCGTTGTGCATAAATAGAGTGGCAGGGGACGCAGTAGACCATTGCACGGTGCACAACACCACTGTCAGCCATCGCAGCGAAAAGCCGATGCAAATTAGCATATTGAGCAGGGCAACAACAATTGAAATAGAGCAAAACATGTGCACTCACTTGGACATCCAAGTTGGGCTCACAGAATTGCTACACGATTTGAACGAATTAGTAAACTTCCAAGGAGTGGATGAGTTTGTCGCTAAATACAGAAGCAGTTTTTCTACTCAAAGGGAATTAGAGGCAAACTTGCGATTAACCGGCCAAGTGCACACCATCAGGACCAAATCCACACCAATCATTCCAGGCGAGTGGGTAAATTGCGCCAGGCTAAAATACTTCAAAAACACCAGCGACAAATTGAAACCAGGAGAGTGTGTCCTGTGGACAGATGGCGTGCACATGCATTGGGCCGCTGTGATATCTTCAAATAGACACGGCCAGTACGTGTGGACACCATGCCCCATGACGACCGCAGTTCCTGTGGTACTACGAATTTCCATGAAGTGGGCCAAACCAGCACCACAGTCACCCATAGCAACAAACACAATATTGGGCGAGATCCAGCATGTCACAGCACTCAATGAGGCGACCGCACGGCACATGGTATCACACAAGCTCGTGGTAGGTAAACCAACAATTAACAATCCACATGCAACAGTGCTGGTTGTAGCGGATTTTGACAATCGCAAGCATCATAAATTCGACGACAGAACGGTCTTGCGTTCTTGGAATAGAGAAAATATTGTGGTGATCAATAAGTGCCCAGGAAGGGTGCCAACTGAAGTGTTTTTTGAGGATAGCGACATGATGACAGCAGTGTTGTACCAGGGCGACCCTGAGATTCACATAGTGACAGACCACACCTCAACTGCCCATTGGGTCAAGAGCTTCTTAATGAGAAACAAGACGAGATCAACGGTGAAGCGTAACGTGGTGGTAATTTCAGATTCTTGGCACACGTTGCAAGAAAAGAAAACGCAGGAAAGGCTTCAACCTCACGTGCACAAGATCAATGACATGTGGCACCTAAACAAACTAGTTCCTGGGACCTTTACTATTGGCACGGAACAAGAGTTGTGCCAACTATTGCAAATTACTGAACCGGATTTAAGTGTGACAGGCCTGTTAGCTAACCTACCGTTGCAGCTAACAATACACACAGCATTCCACGAACCCTTAATGCACACCAGCTACCACCGAAGCCAATTAATAATGGTTCGCAACGACAAATATGTGCGGTCCGGGTTCGTCAACAAAATACAAATCAAGGCAGCCCAACGTGGTGGTTCTTGGATGGAAATCCCAGCAGGAACAACTGCAGGAAAATGGTGGGACCAAGAACAGGACGCACATCAACTGCCAAACATTGACGTGTCAATAGGTAAGGTGGAAGCCATGGTTGGTGCAGCCATAACTAACATCCCATCCACTCAGAATCGAACATCAACGCATGAAGGGAAAACCGCAATAATTCCTTGGATGGACTACCCCATGTACGTCGACACCAGCATAGCGGACACACCAGCATTAGAATTGGTTCCAGCCCAAATTATGGACATGTGGGACACCACAGATTTGAGTGGGTATTTAGACAGATTTGCACCAGAAAACCAGTTCAGCATCAAAGTGAAAACTGTGCCCACAAAGTTGGCACAGTCGATAAAGAGCACACTAGTTAAATACCCAATATACAGCAGGCCAGTATACACAAAAATGGCCAACCAAGAATTTAACGCAGTGACAGGAAGGCTGGGTAAAGTAACACAGTACAGAGTGGGGGATTATTGTGCAATCAGCGAGGCAAACGGCATAGCAAACACATTTTTCCAAAAAGATTGGAAGCATGTGGTGGCAGAATACCAAAGCAACACAATAACTTATAACACAGACAAAGTCAAAGAATGGTTGCAGGGCAGACCTAATGTGGAAAGTATAGTCGGGGAGGTTAACAACATTCTAACGGAAGGATTTATGAAACACCCAATCAACAAGCTCAACGTTCACCTCAAACTGGAATCTCTGTTGAAATCACAACCAGTTGTGGATCATAGGCAAACAAAGGCCAGGATCTTAGTATGGCAAGCAAAAGGCATGTGTGCCATATACAGCCCCGTGTTCAAAATGGCAAAAGACAGGTTAAAATCACTGCTAAACACAAAAACTGTATATGCAGACGGATTGCGTCCGGACCAATTAGCACTGAGAGTGATGCAAGCGGATCCTGCCAATTACATCATGGAGAATGACCTGGAACAACAAGATAGGCAAACCGATGACAAACTGTTGGATGTGGAAATGGCAATTTACCACATGTTGGGTGTGCAGGAAGAACTGTTGTCGCTATGGAGGCAGTGCCATAACAACTGGAATTTTAAGGGAAGGTCATGTCGCGGAGAACGAAATTGGATGAGGCTAACAGGCCAGGCCACCACTGCACTAGGCAACGCAATAACCAATTTGTGCGTGCATTGGCGGCTGTGCAGCAAATTGGGCAACGATTGGAAACTGTTTGTGTTATTAGGAGATGATGGAGCGTTATTGACCAACAGGACATTGTCAAACAAGGAGGTAAAAACCCACGGAAAATTGTTGTATAACATGATAATGAAACCCAATACACACAATAATGTCGGCACATTTTGCTGTTTCAACATATACAAGTTGCCCACAAACCGATGGACCATGGGCCCCGACATTGCAAGACTGCGAAGACGGTTTGAAGTAACTAACGGGGCGTCAGAGGCCACCCTTGTCAATCATCACGCAAGGTGCATGTCATATTGTTGTATGTTAGGATCAATATTACCCATTCAACAATTAATCAAGGAGAGGGGTTACACCGTAGAGCCACCAATGTGGTATGATTGGAACTCCAACGCAAGAGCCACAGAGGACAAGTACCACTGGCTACCAGGGAAAAGTGACACAGAGCTGCGCATGCTGCTGGATATGATGAGACAAACAAACTACATCGATCATAAACTGTTGCACTGGGTAGAAGCAACCCGCTAGTGCTCCTACCCACGTTTTGGTGACGAACAAGATTGCCCCCCCCCC

>HvEV_HYT37_MN107382

gTCAACCAACCATGTACAACCAAACAGTACATGAGACAGGCAATTCGTCACCAAAACAAAAAGAGGTCGAGTGGCTCAAAGCCACTCGACCTCTAAAAAGAGACGTGAATAAACGATGGAAGGCAAAGCTGGGAAAACCAAAATTGCGACAAAAACCATTAAGATTGAAGCGTCTCAAAGGGCAAGTGGTGAACCGATCCAACAACATGAACAGCCAGTACGACAACATGAAAAATTATTACAATCAAATGACAAAAGTACGCATATTAGGTAGAACCATGGTACCCTGCGCATTGGCTTTGCAATTTCCTATGTCTGAACCATTGGTTAATAATTACATGGAAGCAAGTTACATCGCCACATACAAACTGATGTACAAAAACAAAGAAAGTTACGAAGGGTTGCACCCGCATGAACTGGCTGCATTCATAAAAGGTGTTAGATTAAATGTGGAAGACAGTTTCAACCATGCGGGCAACAAATTATTCTATGACTCTGTCGAGCAAGAGGTGTCAACTCACACCATAGACACACCAAACGATCCAATATTGTGGCAGGCAATTAGGGAGATCGAAGCCAAGTACGGCGGAGCCATTGGCATGCAATACCAGGCTCTGCGCAATTGTTGGACAAGAGTTCCACACCTGGCCAAGGTTGGCGGTAGACACCAATTGTTGATGGTAGATGCAGAACCATGCGGACACAGGCGTTGCGCGGAGTGCGGAATCATGAATGGCTACTGGATCCCTGGCGAGGATGGATTGTCCAATGCACACTGCGGGGCCTGCGGCACTTCATTAGATTTAGAGAGACAAACTCCGGAGCCTCTCGAAACACCGCCAACCGAAGCTTTCCAGCAAATCAACCCGCTCATGGGTGCTTTCGAAATGGCGCAGATGGAAATTATTGAACAGTTAGATGGAGTAGGAGATCTGGAGGGGGTAGCACTGAAGCAAGCGATAGAAAATGATGAAAGACTGATAAGAGCGATGATATCAAGGGCGAACGTCAAGTTGAGTTTTGTTGATCCGGGCATGACACCTGAACAACGTAAATTAGTAGGTTCAAGCTTCAAAAATTTTATCTTGATAGACAAGGTAGGACCAATAAACGAACATGCTATGATCGCGGCAGAACGTCAGCTCATGGCACAAGTTATGTATGACATGGCAGCCGGAGGTCAACCCATAGTGGACATTGGAGGGCCTAGCCCCGGTTGGACGGCAGTTAAATCGTACCAAACGGTGAGCCCCATCCTATCAATCAAAGACGTTAAACGGTTCAACAACTACGACAAAATGCCGGTGCATTGCACACACACATTGAAAGAATGTAATTGTCACACGGGACAAGCACCATTAATCATGAGTGTTGACAGCTTGTATGATATATCACCAAAGGATGTTATGAAATTTATGTTACTTGCCAAATCAAATTATATAGTTTACTGTTTGAGCACAGCCACACTGGATCACTCCACAAACAAGGGTGTGCTGCCTTGGGACCAAGGGCTGTGGATAAAGCATCAAGGCAAGTTGGTGACAACATTCAATGGGTCATCTTCGACGTATGAGAACGATTGGGCCCAAACCTTATGGTGGACCAAGGCAGACGTAATAATAGTGGGGGACCATAGTTTATGCATACAGACCCTAAAAGTATGCGGAAACCACATTCTACGTGTAGCTACAATATGCAATGACTACAAAACACAGTTTTTCTCACTGAATAAACCCGTTATAATGAGTTCATCTTCCAGTGTGTGTATGCCGATCCCTGTGTGGAAAAATCCAATCAGTCACGTTGTCAAGAACGTGGTGGAGTGGAGAACGGTGGCCGTAGAAAAAGAATTTCTTAAATTGTTGTTAGTGAGAGCGAGCGCAGGTCAATGCTCTTATGATGACTTAGTCAAATATGCAATAGGATTGGGTTACAGCAAGTACACACTAAAAGACAAGGTAATATCCATGAATTATATAACTGCAGATATGGCATTaAACCATGCGTTGATAGCAAAGGCCATCACTGATAGAAAGCTGCTAGAATCAACTTACATCATGGCACACGACAAACAATTTTACGATTGGCATAATATGAGCCAGACGGACGTGTTACAGACCATGGGCACGTTGATCTTCGAAATTACGAAACACCTGCTCTCAAAGTGGTCTGCATCCGACGACAAGGTCCAGTTTGTGTTCAACAAAGTGGTTGACGGTATATCTGAATGGATAGCTGATCCAGCATGGTACAATCTGGTGGAGGCGATTACATTTGATGACATTACGAAGATGAACATTATTAGAGTAAACACATCCAGCCCGGACACAAACGAAGCCATCATATGTGACCACCACAGCGACCATTGCAGTCACACTGTACAACTGTCGCCACATTATTGTCAATGTTGCGGGTTGTACCCcgtcgTGGTTGGTGAAAACCGTTGCGGCTGTTGCAAAAGTGATCACTGCCATCATAAATGTCACCACAAGTGCACAAACAGAGCGGAGCATCTCGCCGGCACAAAAACAACACCTCGGGAATGCACCTGCTGTGGTGTTGAGTACGACACATACGTGTGCGTCGTCTGCAACAGACCTGATGGGTACAGTGTCGTGACGGACGCAGAAGTGAGGAAGAGAGTACATGAGGCAGAGTTAGCTGACAAGCAAAGAAAATCCAACTTGAAACAGTCCAAAACGCAACAGTCACTCGAGCAAGAGGGTGACACGGGCAGAGGCAAAGCAGCAACCAAAACTTACAGCGCTGATCTCGACCCGAGTACCACCAACGCAACCAAcgtggcgcatgcagcacccatcacgGAGCCAACGGCACCGCTCGCAGACCCATCGAGTGACCAGCGTAACACCGCCACGCCAACCGTGTATGATTTGCTGAAGCTGCACATGGACGACTTAATTGAAAGTCCCACACCAAGGCTGTATTCAGATGTATTAAGTTTATCTGCCGCAGAGTCAACACCCCAGGTCAACGAACAGCCGACCACACAAAGCCGCGACCCACCAGCAGTTGAAACTCGGGAGCCGAGCATAAATGTTGTGAAACCAGTCATGACAATGAGCATCAATCACCCAACTGCCACAACAACCGCCACGGACATTGAGGCGGGCAATGTTGAGGGACGCCTTTTCCTTGAACTGGTTAACTTAACAAGCTTGGAATTTGCCAAAATTATAGCTGACAATGAGCGGGGATGGGCATGCGTTGAAAGTGAAACAGAAGTGAATCTCACTAAAGGTTTATCTCAAATCAAATATGTTGGGAGGAATATCACGGTCACAACCCCAGACAATTTTGAGGTAGTTGAAATCACTGACGTTAGTGGCCAAGGCAACCAATGTGGTTATAACGTCATTAATGACACCGTGGGAGTGGACAATGACGTGTGGACTTCAATTATTAGTTCACGACAAATGTACAGCGACATGGAAGTGCAGCAATACTGCACAATGATGAGCCTAAATTTACTGCTATTGGGTAGGACAAACACCTTCTTCCATCGTCCAAACACAGCATCAGACACATTCATTGTCATGCGCCATTGCAGCTTGGACAGCCCAGAAGGCACTCCAAAAGAGCTATGGTTTAAACATTGGCAGCGTGCGAATGTAAAAGTTAAGTTTTCAAGGCCATGGGTCCCGATTTTCTACCCATGGTTGGAAAGGGCAGCCTGGGACAGGTTCATGCTTGATAACAGAATTAACGAAGACTTTTGGCAATTAGCGACCGAGCAAAGACTACAATTGATGGTTGAATTTTACACAAACAATTTTGTGTGTAGCAACCTAAAAGAAGGATATTTGGCCATGCTAACAATGGATCTAAAGGCATGCCCGCCCGTCATTTACAATAACTCATTGATGATGGACCAACCGGACACACACCTAAAAGGCTGTGTGGTAAGCAAACAAGGTGTGGCAAAGTTACCATACGCTCTGACGCAGCACCAGTCCATAGAGCAAGACGCAATAATGGGTGCCCCCATTAATCAGCAACCACCAACTAATGAGTCAGAACTGGAAGTGTGGTTGAATAATGAAATTAAGTTAGAATGCATTGATTATGCATACAACATGGAACACATGATGTTATTGGAAGAGGAGATGCATTTTAAAGAGTGGCAGACAGTCAACGTAAAATTTGTGGGTCAGCGTCTACGATTCTCAAAAGGCTTATTCAGAAAAATTAAAACAGGTGACATAATTATGATCAAGGTGGCAGGTTACAAAATGACAACAACGGTGACCATCAGCATGCGCCAAGTGCTTTGCGACAAGCCGTACACCGGACCAACAATTGACCAGGCTCTGGTGGGCGTAAATAAACAAAGTCATGCCTCTAAAATGGCACAAGTGATGACGTTATTGAGCAAAAACCTGGACGCTAAGCACATATGGGATAAATTGAAATCAGCAACAGTGATTAAGGGCCCAGGCGGCAGCGGAAAAACCACCGGGCTGCTTTGTGAAGCACACAAAGGCAGCATAATTTTAACCAAAACAACCATGGCAAAAACCAACATATTGAAGCACAACCCACCCTGCCCCGTCATGACTCTAGAGGCATACCACAAAAAGGATGATCAACATACAAAAATACTCATAGACGAGGCTGGCATGTTCTCACTGCTAGATTTTGCCACCTTAAGAATGCAAACAAACACACAAATAATTATGAGCGGAGATTTATTCCAAATAGGGCAACTAATCACCCACGTGCACCCAGGAattaGGGATTACCAACCTTGCATCAATTTTCCTGCAAACGTAACCACACTGAATTCAACATATAGGTACGGCAAGTCTGTGTGTGATGTGTTACACACAGCCGGTATGAACGTGACGTCGTTGGCACCATATGACACAACTATAACCCTATTAGACATTAAGGAAATCAGATTGGGAACGATCAAAGAGTTAGTCAAAAAGTACACCCCGGACGCAGTATTGACATTTTACCAATCACAAAAGAGTCTAGTGGAATCATGGCAGCTGGACCCAGACACCCACACCGTACACGAATTTCAAGGAAATGAGTGTGACACAGTTATGGTGTTGCAAGGTCCACAAACCATAGCGACCACGGGAATATGGAAtAAACCAGAATATTGTATTTCGGCACTGACTAGGGCCAGGAAGCACGTGGTGTGGGTCACTTTTAATGCAACCACGGGCATGAACATGGTACAACGGTGTGGTTTAAGCCCAGAACTAAGGGCCACCCACAACACCACATCGTTAACCCAAACCTGGGCGGACATGGTGGAAGAAGACCAAACCAAAGAGGCAATGCCGGGCGGTGGGCTGAATCCTTATgggctGACCATCTATGTTGGCCGCAACACCAACCACGTCACAGACTTAAAGTCCATGACCACCATTAAAAACAAAGCAGTAGTGGGGGACGAAGAACTATTTGTAGTGTTGCTGCAGAGTGAATGGTTGATGGGCATGGTTGACGAAAACAAATATTACCTGGTGGCCGGCCAAGAACTAGCCACCATGAAAGAGCCCAAAAATGTGACTGATTGGAGGCGCCTGCTGTCTGACCTCACGATCAGCGAGCAGGCACCACCCCGGTTGCTGCAAGCAGCCAGCAATTTCTCCTCCGCAACGATGATTTTCAAAAACAACATGGTGCACCTCTCTGAAGACAGAGCCGTGTTGTATGTGCCAGACACACAAGCAAAACTGGCCGAGTTAGAACAAAAAATAAGAGCCAAAACCAAGCTGGTGCAGATAACAAGAACCCACAAAGAATTAAAACTCCACTTTCTGCTCACCACGGTGGGGACCATAACAATCACTGGTCGGCACAGAATAGAAGTGTTTGGTAACAGGGCGCTCAACTATTTGACGAGTCATGTCGACCAAGTTAATGATGCTCAATTCAAGGCATGGGTTGCACATTTAATAAATAACTTTAGATGCACAGCGTGCGCAGCAGAGATGTACATTAATCCGGTGGCATTAACTGAGCACTTGTCAGATCCAAAAAGTGACACACAATGGACCGGGTGTAGCAGGTGGAGCTGCGAAAATTTTAAAGCCCCGCCAAGCTCCGAAGAAAACTCTATCGAAGACGTAATGGACACAGAATCGTCTTCGTCAGAAGAGGAACCGGACAAGGAACCTGCCAGTGACGGCACTTTGTTAGGCGACGAAGCAACCACAGGCCAAGAGTGTGAGCATGAATCAGACGAAGAATACTATGACACAATTGACACTATTCACATAAACCCTACGACAGTCTGCACCACACCGCGCAGTAGAAGTGGCCAAGACGATATTAACGACGAAATCGTTCAACTAATGTCAACAGCACAGCCAAGTGGGGAGCAAACCGCAGAAAGCCAAACAACAACAACTTCCCGCACACATCACACATCACCACCACCCAACCCCCAACAACAGGAGCGCACACTAAGTAGTTTGGTGGTGTCAACCTTACATAACAACCAACCCAGACGCACGTCAAGAGAGAGGGTGATGCACCTCGTCGAACAATATGCGCAACACACTACCGGGTGGATTATCTTTCCGCGGTGCATCAGCATCAGGCACATGCAAACAGCTCTGTTACATGGCTGCCCAATGCACTGCAGATGGACTTTACAACGGAGCGAAATGTGCAACATTTTCACCCTCATGGCCGGGCTGACCAGGGTGCTCACCATTGCTTTCAATAGAGCCGGCAACGTGGTTGGCCCCATGGATGGAAGTGTAGCGGACGACATGTACATCACGTTGGCAACACATGCTGTGGATCAATGGCGGTTGGTGGCTTCGGGTCAAATGGAGTTGGATGAAGATTTAGCATTGGAGGGGCTAAAATTTATCAACCAATCACGATTGTCACCGATCCATGTGCATAGGCATGACACTATTGTCATCGACGATACCGAATGGCCGGCATGGATCGGCGCCTCCGTGAAGGAATATGAGCCATTCATGACCAGACCAAGATACCAACAATCTGTGACACACCACGCGATAAAGACCATGCTACCAGAGTTGCCCACTGAAGTGTGCGGGTGCGAAACAAAACTTCTTGACGAAAACAAGATATGCGCCACATACCCTTGGTTGGACACGTACACAATGACACCTGATGAACTTGCAAGTTGCATCAGTTGGATTCAATTCGTGCAGTACCGGCAAATTGGGGGTCTGAAAACCCACACAGTTATAGATGGTACAGTAGTAGACAGCACAACCTTTGGGGGTTGTGCACTGTGCGCAGGGCTGAAGATGACAATAAATGAGGAAATTACAATGTTAATCAATAGTAGATCTGGTCCCGAACCCGGAACCATGTGGGTCAACATCAACCATGTAATACAATATCCACACGTGATATCAGAACTGTGCAAAATTCTAGAAATGCCTGGCATGATCATGCATTATCCAGATCTGGAGCTGTATTGGTCCGTCAGGCAACTAACCGAAATGGACGGCATGATACAAGACTTTGGCCAAGCTATGTCGTTAATTGAGCGACTCAGGGCAGGAGTTGATTGGGTGATCGAAAGGGTCACCAGCTTCAGTTTACAATGGAAGTGTGACCAGTATAACACAGTCAATTCGGAGTACGTGACGATGCTGGGAAAAGCACTGTTGGATGCGGGGGTTGATCCATCATTGCACTGTCATGCAATCACTCATACAGGCACACTATTGGTGGGGCTGCCAACACGATGGTCAGCCTCGGGCCAAACCATTGTCATATCAAAATTTAAGGGCAGATTCCACTGCACCAGTCACTCACTGGCCATCACGCAGCGATGGGAGTGGCAAAGCGCTAGTGACATGCTAGTGGAGGCAGTAAAACACCGCATGCTGATACTCTTTGCAAACAAATTGAGTGGCCGCAGAGAGTTGGTTCGGTCCATTTTGGAAGATTCCGTAGCCATCAATCTGGACGAGGTAAGAAGGGCCATGGGAGCAGTTAAGTTGAACTTGAGTTTGGATTACCACAGGGAGCATAACACCGGGGTGAACGCTATGTTAAATGATCGCAAAAACAAACTGGTGCAGGGCCAACTCGTGGGCCCCCACCAGGCAATGTACTTGTCACCAAATCAAATCCGAATGTTTGGCAGAGACATTAATAGAACATGTTGTACGCACAACTTGACAGAACACGGCACATGGCTACCTGACGAAGGGTTATTTGCCACAGCGGAACAGGTGGCAGCGAAAATTATATCTGGTTGGTGTGGGAAAAACAAATTGTGCACATACTCGGGTACATATAGTTACATACCGTTAGTGAACTCCAGCTGGAACATGAAAAGTGTAAAACCATTACGCAGCACACCGCTATACAGTGCGTGTGCACTGGCTATGAATGGGGCATTAAACAATTTGATCACATCTATAAAAAACTTAAAAGACAAAGCAGAAACCGTGACCGGGTTTGCATTGAGTGATAATGAGAAAGCCTTGATAGCCATGTGTGACAAGGTTGCAACCACAAAAACAAACCCTTGGTGCAGCAGCGAGTCAGTCAGTGATGGCGACAAAGCCATGTTGGGAAGTGAGTATTTGGGCCTAGACTGGCATTCATTTTGTGATCAACTGGTAAAAACCAGTGCAAGAGAGACATTCATTTTGGTTCCCGAGCAAGACAATCACAGCGGCGATTACTACAACATCCATGACAGACAGGAGTATTTTGAATGTCAAGGGGTTGCAAGCACCACATTAATAACCCTCAATCCCACCACATGGAGCAACCTGCTCATCAAACACAGCATGAGGTACAACAACAAAATAATACACGCAGAACAGCTGATGTCAATATTGGGTTTGCGGCTTGTCAGCATAGTGGTGACCTCCTCAGCAGTGTCAGTGCCTGGTCACTACTCACCATGCATACCAAACAACAGCAATCAAGTGGTCTACAAAATTAATACCCCACAATTGTCATGGAACACATCCGTTTTCAGCCACCACAAGACAACAGTAAAGGTGGACTCCAAAATGTACCGCCACCTAAGCTTCAGAGTTGTGTCCGATACGTGCAGCTTCGAAGATTTATTGTCCTACGCCAGAACGTACATGCAAACCACCACTTATACTCAGACTGGATACTACAGGGAGAATTTAGATCAAATCaatgatttgCATCTCATTTGCGCGTGCGTTTATCACGAATATCAGCAGAAAAAGACAAGCTGGTGCACACTAGCCGCGAAACATTGTGACTTACCAACCATGGATGTGGGCCGGGTAATGGCACATTTGAACACCAATTTGACCCATGCCGCCTTCAAGCTGATGCAAGCATGGAAGTTAAATGACGGAACTTTGCACGACATTAAATTGGCTTCTAAAAACACGATAGGACCTGAAATGACAGACACAATCAAAAATCTTGCCACAATGAACATTGAGAAGAAAGAGGTGAGCCGGCAAATTATGGTGCAAAACGGAGATCGCGCTTTGCCAGACATCACGACAAATTGTCCATGTGAGAGGCAAATGACCAAATCTAATGATGTGGTGATGCCAATACTGGGCGGATTGGTCAATGATGGATCATTGCTGACAGAGTTTTTGGCGGAAGAACTACGATCAAACTCAAGTTATTGGTGCTTAGTACAATCCAGCACGGATGGTACTAACACAGCCGTACAACTGGGGGACGGAGTCAGGCGTTCTCACAATTTGTCAAGCGTGAACCTCAAAAAGAAACGGAGGTGCAAACACCCCGGCAGTGAAACAAACCTCACGGAGAAGTACATACAGCAAAGGATGGAAGTCACTGCCAATATGAACAGCGCAAACGACACTCAACCACGTGAGTCAGATGGCCCacctatcacaGACACACGCAGCACACCAACAATGACCCGGTGCAAGACTAGTGGCCATGAACCATCAGTTGACAACTTGCATCAACCTGCAgcgagtaataatgaggacGGGGACACCACTGAAAGTACTTGGAGACTAATGGAACTATCGCATGTCATCATCAAACGAGTGTCCCAAGCTGTCACCAGCAGGGTGGGCGAGTGCCGGCAAAACAATTGCAAGGCCAGATCTAAGGAAATTAAAAGACTGTTGAAAGATTTAGCTTCAGCAGTGTGCCACAAGTGCAACACCTGGGCAGAGGAGCATTTTGCGGCCAGCAGGGACCATGAAGAGTCTGGGGCCACGGAACCGCTGAATGGAACTACGACACCCGAGCCAACACCGCGCAGCACAAGACCCACAAGCGGAGAAAGAGTGTGGGTGTTGATGTCCCCCACACAACGCGTTGGCATCTTAATGCAAGAATTAAACCAGGCCCATccacatgccgagctctcacaattgagaactgtgactagtgACATGAGTCATGACAACGACAACACGGACAACGAGATACGCGCAAAGGCCGACCAATTGGCAGAGCTAATGGAGAATGGGTTTTCATCAGAATTTGACCGGACCGACGCCATCAGCTCGTACGACGATACCATCTCGGAATGTGAGGAGCCCACCACGTTTGTTAGACAGCAGTGGCAGCAGTACGATGTGGACCATGtgatcacAGAAGAGTTCCATATCCAAAGCGACTTGGAGGAACCACAGTTGACCTCCAGAACCCCAGAGCCAGCGTTGAGCGAGGCACAATCACAGGACGACAAAACCGCATACGCACGCATGTGTGACaagaccTGCAAGCTGTTGAGTGACACAACGGCTAACGCTGCCGGGTTGCTGGAAGATTATATTGAATCAACAACACTAGAATGGTTGACTACAGAAGCAAGGCAAGCCAACCGTACGTGTTGGTGCCATGTGACCACACCCGAATGTTTACAAGAGTTAGTAGGAATTGACGCATGCCATGTCATTGTCAAACCATATGCTCACATTacAGGTGTGGCCACCAGAACCAAAGAAATATACAATCCCAACACAGAGGGAAACTGCGTGGTAAATAGCCTGAACTATCTTGAGCAACACCACCCCAGAGTAGACAAAGTAATGGAAAGGGCTTGCGGCGTGTACGGGAAAACCATGTGGTTAACGGAGTCCGACGCGGCAGCCATTAGCTGGTTATGCAACCTAGAAACTCGTTATAAACACAACCAAACCGTGCATGAATTGGCGACTGGGTCAAGCGCCTCCCCATTATGTATAAATAGAGTGACAGGGGACGCAGTTGACCATTGCACGGTGCACAGCATCACCGTCAGCCATCGCAGCGAAAAGCCGATGCAAATTAGTATAGTGAGCAGGGCGACAACAACTGAAATAGAGCAGAACATCTGCACCCACTTGGACATCCAAGTCGGGCTCGCAGAATTGTTACAAGATTTGAACGAATTAGTAAACTTCCAAGGAGTGGGAGAGTTTATCGCTAAATACAGAAGCAGTTTTTCCACTCAAAGGGAATTAGAGGCAAACTTGCGATTAACAGGCCAAGTGCACACCATCAGGACCAAATCTACACCAGTCATCCCAGGCGAGTGGGTAAATTGCGCCAGGCTAAAATACTTTAAAAACACCAGCGACAAATTGAAACCGGGGGAATGTGTCCTGTGGACAGATGGTGTGCACATGCATTGGGCCGCTGTAATATCTTCAAACAGACACGGCCAGTACATGTGGACACCATGCCCCATGACGACCGCAGTTCCTGTGGTACTACGAATTTCCATGAAGTGGGCCAAACCAGCACTACAGTCTCCTACAGCAACAAACACAATATTAGGCGAGATCCAGCACGTCACAGCACTCAATGAGGCGACCGCACGGCACATGGTATCACACCAgctcgtggTGGGTAAACCAACAATCAACAATCCACATGCAACAGTGCTGGTTGTAGCGGATTTTGACAATCGTAAGCACCACAAATTTGACGACAGAACGGTTTTGCGTTCCTGGGATAGAGAAAACATTGTGGTAATCAACAAGTGCCCAGGAAGGGTGCCAACTGAAGTGTTCTTTGAGGAAAGTGACATGATGACAGCAGTGTTATACCAGGGCGACCCTGAGATCCACATAGTGACAGACCACACTTCAACTGCCCATTGGGTCAAGAGCTTTTTGATGAGAAACAAGATAAGATCAACAGTGAAACGTAACGTGGTGGTAATCTCAGATTCTTGGCACACGCTGCAAGAAAAGAAAACGGAGGAGAAGCTTGAACCTCACGTACACAAGATCAATGACATGTGGCACCTAAACAAACTAAttCCTGGAACCTTCACCATTGGCACGGAACAGGAGTTGTGCCAATTACTGCAAATTACTGAACCGGACTTAAGTGTGACAGGCCTGTTAGCTAACCTACCGTTGCAGCTGACAATACACACGGCATTCCACGAACCCTTAATGCACACCAGCTACCACCAAAGTCAATTAATAATGGTTCGCAATGACAAATATGTGCGGTCTGGGTTCGTGAACAAAATACAAATCAAGGCAGCCCAACGTGGTGGTTCCTGGATGGAAATTCCAGCAGGAACAACTGCAGGAAAATGGTGGGACCAAGAACAGGACGCACATCAAGTGCCAAACAATGACGTGTCAATAGGCAAGGTGGAGGCCCTGGTTGGCGCATCCATAACTAGCATTCCATCCACTCAGAACCAAACGTCAACGCACGAAGGAAAAACCGCAATAATTCCTTGGATGGACTACCCCATGTACGTGGACACCAGCGTAGCGGACACACCAACATTGGAATTGGTTCCAGCCCAAATTATGGACATGTGGGACACCACAGATTTGAGCGGGTATTTGGATAGATTTGCACCAGAAAACCAGTTCAGCATCAAAGTGAAAACTGTGCCCACAAAGTTGGCACAGTCAATAAAGAGCACACTAGTCAAATACCCAATATACAGCAGGCCAGTGTATACAAAAATGGCCAACCAAGAATTCAACGCAGTAACAGGAAGGCTTGGTAAAGTGACACAGTACAGAGTGGGGGATTATTGTGCAATTAGTGAGGCAAACGGCATAGCAAACACATTTTTCCAAAAAGACTGGAAACAAGTGGTGGCAGAATACCAAAGCAATACAATAACCTATAATACGGACAAAGTAAAAGAATGGTTGCAAGGCAGACCTAATGTAGAAAGTATAGTTGGGGAGGTTAACAACATTCTGACGGAAGGGTTTATGAAACACCCAATCAACAAGCTTAACGTTCACCTCAAACTGGAATCTTTGTTAAAATCACAACCAGTTGTGGACCATAGGCAAACAAAGGCCAGGATTTTAGTATGGCAAGCAAAAGGCATGTGTGCCATATACAGCCCAGTGTTTAAAATGGCAAAAGATAGGTTAAAATCACTGCTAAATACAAAAACTGTGTACGCAGATGGATTGCGTCCGGACCAATTAGCACTGAGAGTGACGCAAGCGGATCCTGCCAATTACATCATGGAGAACGACCTGGAACAACAGGACCGACAAACCGATGACAAACTGTTGGACGTGGAAATGGCAATTTATCACATGCTGGGTGTGCAGGAAGAACTGTTGTCGTTATGGAGGCAGTGCCATAACAACTGGAACTTTAAGGGAAGGTCATGTCGCGGAGAACGAAACTGGATGAGGCTAACAGGCCAGGCTACCACTGCACTAGGCAACGCAATAACCAATTTGTGCGTGCATTGGCGGCTGTGCAGCAAATTGGGTAATGATTGGAAACTgtttgtgttattaggAGACGATGGGGCGTTGTTGACCAACAGGACATTGTCAAACAAAGAAGTGAAAACcCATGGTAAATTGTTGTACAATATGATAATGAAACCCAATACACACGACAATGTTGGCACATTCTGCTGTTTCAACATATACAAGTTGCCCACAAACCGATGGACCATGGGCCCCGACATTGCAAGACTGCGAAGGCGGTTTgaagtaactaacgGGGCGTCAGAGGCCACCCTTGTCAACCATCACGCAAGGTGCATGTCATATTGTTGTATGTTAGGATCAATATCACCCATTCAACAATTGATCAAGGAGAGGGGTTACACCGTAGAGCCACCAATGTGGTATGACTGGAACTCCAATGCAAGAGCCACAGAGGACAAGTACCACTGGCTACCGGGGAAAAGTGACACAGAGCTGCGCATGCTGCTGGATATGATGAGACAAACAAACTACATCGATCACAAACTGTTGCACTGGGTGGAAGCTACCCGCTAGTGCTCCTACCCACGTTTTGGTGACGAACAAGATTGCCCCCCCCCT

>HvEV_HYT38_MN107383

ATCAACCAACCATGTACAACCAAACAGTACATGAGACAGGCAATTCGTCACCAAAACAAAAAGAGGTCGAGTGGCTCAAAGCCACTCGACCTCTAAAAAGAGACGTGAATAAACGATGGAAGGCAAAGCTGGGAAAACCAAAATTGCGACAAAAACCATTAAGATTGAAGCGTCTCAAAGGGCAAGTGGTGAACCGATCCAACAACATGAACAGCCAGTACGACAACATGAAAAATTATTACAATCAAATGACAAAAGTACGCATATTAGGTAGAACCATGGTACCCTGCGCATTGGCTTTGCAATTTCCTATGTCTGAACCATTGGTTAATAATTACATGGAAGCAAGTTACATCGCCACATACAAACTGATGTACAAAAACAAAGAAAGTTACGAAGGGTTGCACCCGCATGAACTGGCTGCATTCATAAAAGGTGTTAGATTAAATGTGGAAGACAGTTTCAACCATGCGGGCAACAAATTATTCTATGACTCTGTCGAGCAAGAGGTGTCAACTCACACCATAGACACACCAAACGATCCAATATTGTGGCAGGCAATTAGGGAGATCGAAGCCAAGTACGGCGGAGCCATTGGCATGCAATACCAGGCTCTGCGCAATTGTTGGACAAGAGTTCCACACCTGGCCAAGGTTGGCGGTAGACACCAATTGTTGATGGTAGATGCAGAACCATGCGGACACAGGCGTTGCGCGGAGTGCGGAATCATGAATGGCTACTGGATCCCTGGCGAGGATGGATTGTCCAATGCACACTGCGGGGCCTGCGGCACTTCATTAGATTTAGAGAGACAAACTCCGGAGCCTCTCGAAACACCGCCAACCGAAGATTTCCAGCAAATCAACCAACCCATGGGTGCTTTCGAAATGGCGCAGATGGAAATTATTGAACAGTTAGATGGAGTAGGAGATCTGGAGGGGGTAGCACTGAAGCAAGCGATAGAAAATGATGAAAGACTGATAAGAGCGATGATATCAAGGGCGAACGTCAAGTTGAGTTTTGTTGATCCGGGCATGACACCTGAACAACGTAAATTAGTAGGTTCAAGCTTCAAAAATTTTATCTTGATAGACAAGGTAGGACCAATAAACGAACATGCTATGATCGCGGCAGAACGTCAGCTCATGGCACAAGTTATGTATGACATGGCAGCCGGAGGTCAACCCATAGTGGACATTGGAGGGCCTAGCCCCGGTTGGACGGCAGTTAAATCGTACCAAACGGTGAGCCCCATCCTATCAATCAAAGACGTTAAACGGTTCAACAACTACGACAAAATGCCGGTGCATTGCACACACACATTGAAAGAATGTAATTGTCACACGGGACAAGCACCATTAATCATGAGTGTTGACAGCTTGTATGATATATCACCAAAGGATGTTATGAAATTTATGTTACTTGCCAAATCAAATTATATAGTTTACTGTTTGAGCACAGCCACACTGGATCACTCCACAAACAAGGGTGTGCTGCCTTGGGACCAAGGGCTGTGGATAAAGCATCAAGGCAAGTTGGTGACAACATTCAATGGGTCATCTTCGACGTATGAGAACGATTGGGCCCAAACCTTATGGTGGACCAAGGCAGACGTAATAATAGTGGGGGACCATAGTTTATGCATACAGACCCTAAAAGTATGCGGAAACCACATTCTACGTGTAGCTACAATATGCAATGACTACAAAACACAGTTTTTCTCACTGAATAAACCCGTTATAATGAGTTCATCTTCCAGTGTGTGTATGCCGATCCCTGTGTGGAAAAATCCAATCAGTCACGTTGTCAAGAACGTGGTGGAGTGGAGAACGGTGGCCGTAGAAAAAGAATTTCTTAAATTGTTGTTAGTGAGAGCGAGCGCAGGTCAATGCTCTTATGATGATTTAGTCAAATATGCAATAGGATTGGGTTACAGCAAGTACACACTAAAAGACAAGGTAATATCCATGAATTATATAACTGCAGATATGGCATTAAACCATGCGTTGATAGCAAAGGCCATCACTGATAGAAAGCTGCTAGAATCAACTTACATCATGGCACACGACAAACAATTTTACGATTGGCATAATATGAGCCAGACGGACGTGTTACAGACCATGGGCACGTTGATCTTCGAAATTACGAAACACCTGCTCTCAAAGTGGTCTGCATCCGACGACAAGGTCCAGTTTGTGTTCAACAAAGTGGTTGACGGTATATCTGAATGGATAGCTGATCCAGCATGGTACAATCTGGTGGAGGCGATTACATTTGATGACATTACGAAGATGAACATTATTAGAGTAAACACATCCAGCCCGGACACAAACGAAGCCATCATATGTGACCACCACAGCGACCATTGCAGTCACACTGTACAACTGTCGCCACATTATTGTCAATGTTGCGGGTTGTACCCcgtcgtgGTTGGTGAAAACCGTTGCGGCTGTTGCAAAAGTGATCACTGCCATCATAAATGTCACCACAAGTGCACAAACAGAGCGGAGCATCTCGCCGGCACAAAAACAACACCTCGGGAATGCACCTGCTGTGGTGTTGAGTACGACACATACGTGTGCGTCGTCTGCAACAGACCTGATGGGTACAGTGTCGTGACGGACGCAGAAGTGAGGAAGAGAGTACATGAGGCAGAGTTAGCTGACAAGCAAAGAAAATCCAACTTGAAACAGTCCAAAACGCAACAGTCACTCGAGCAAGAGGGTGACACGGGCAGAGGCAAAGCAGCAACCAAAACTTACAGCGCTGATCTCGACCCGAGTACCACCAACGCAACCAACGTGGCGCATGCGACacccatcacgGAGCCAACGGCACCGCTCGCAGACCCATCGAGTGACCAGCGTAACACCGCCACGCCAACCGTGTATGATTTGCTGAAGCTGCACATGGACGACTTAATTGAAAGTCCCACACCAAGGCTGTATTCAGATGTATTAAGTTTATCTGCCGCAGAGTCAACACCCCAGGTCAACGAACAGCCGACCACACAAAGCCGCGACCCACCAGCAGTTGAAACTCGGGAGCCGAGCATAAATGTTGTGAAACCAGTCATGACAATGAGCATCAATCACCCAACTGCCACAACAACCGCCACGGACATTGAGGCGGGCAATGTTGAGGGACGCCTTTTCCTTGAACTGGTTAACTTAACAAGCTTGGAATTTGCCAAAATTATAGCTGACAATGAGCGGGGATGGGCATGCGTTGAAAGTGAAACAGAAGTGAatcatactaaAGGTTTATCTCAAATCAAATATGTTGGGAGGAATATCACGGTCACAACCCCAGACAATTTTGAGGTAGTTGAAATCACTGACGTTAGTGGCCAAGGCAACCAATGTggttataacgtCATTAATGACACCGTGGGAGTGGACAATGACGTGTGGACTTCGATTATTAGTTCACGACAAATGTACAGCGACATGGAAGTGCAGCAATACTGCACAATGATGAGCCTAAATTTACTGCTATTGGGTAGGACAAACACCTTCTTCCATCGTCCAAACACAGCATCAGACACATTCATTGTCATGCGCCATTGCAGCTTGGACAGCCCAGAAGGCACTCCAAAAGAGCTATGGTTTAAACATTGGCAGCGTGCGAATGTAAAAGTTAAGTTTTCAAGGCCATGGGTCCCGATTTTCTACCCATGGTTGGAAAGGGCAGCCTGGGACAGGTTCATGCTTGATAACAGAATTAACGAAGACTTTTGGCAATTAGCGACCGAGCAAAGACTACAATTGATGGTTGAATTTTACACAAACAATTTTGTGTGTAGCAACCTAAAAGAAGGATATTTGGCCATGCTAACAATGGATCTAAAGGCATGCCCGCCCGTCATTTACAATAACTCATTGATGATGGACCAACCGGACACACACCTAAAAGGCTGTGTGGTAAGCAAACAAGGTGTGGCAAAGTTACCATACGCTCTGACGCAGCACCAGTCCATAGAGCAAGACGCAATAATGGGTGCCCCCATTAATCAGCAACCACCAACTAATGAGTCAGAACTGGAAGTGTGGTTGAATAATGAAATTAAGTTAGAATGCATTGATTATGCATACAACATGGAACACATGATGTTATTGGAAGAGGAGATGCATTTTAAAGAGTGGCAGACAGTCAACGTAAAATTTGTGGGTCAGCGTCTACGATTCTCAAAAGGCTTATTCAGAAAAATTAAAACAGGTGACATAATTATGATCAAGGTGGCAGGTTACAAAATGACAACAACGGTGACCATCAGCATGCGCCAAGTGCTTTGCGACAAGCCGTACACCGGACCAACAATTGACCAGGCTCTGGTGGGCGTAAATAAACAAAGTCATGCCTCTAAAATGGCACAAGTGATGACGTTATTGAGCAAAAACCTGGACGCTAAGCACATATGGGATAAATTGAAATCAGCAACAGTGATTAAGGGCCCAGGCGGCAGCGGAAAAACCACCGGGCTGCTTTGTGAAGCACACAAAGGCAGCATAATTTTAACCAAAACAACCATGGCAAAAACCAACATATTGAAGCACAACCCACCCTGCCCCGTCATGACTCTAGAGGCATACCACAAAAAGGATGATCAACATACAAAAATACTCATAGACGAGGCTGGCATGTTCTCACTGCTAGATTTTGCCACCTTAAGAATGCAAACAAACACACAAATAATTATGAGCGGAGATTTATTCCAAATAGGGCAACTAATCACCCACGTGCACCCAGGAATTaGGGATTACCAACCTTGCATCAATTttcCTGCAAACGTAACCACACTGAATTCAACATATAGGTACGGCAAGTCTGTGTGTGATGTGTTACACACAGCCGGTATGAACGTGACGTCGTTGGCACCATATGACACAACTATAACCCTATTAGACATTAAGGAAATCAGATTGGGAACGATCAAAGAGTTAGTCAAAAAGTACACCCCGGACGCAGTATTGACATTTTACCAATCACAAAAGAGTCTAGTGGAATCATGGCAGCTGGACCCAGACACCCACACCGTACACGAATTTCAAGGAAATGAGTGTGACACAGTTATGGTGTTGCAAGGTCCACAAACCATAGCGACCACGGGAATATGGAATAAACCAGAATATTGTATTTCGGCACTGACTAGGGCCAGGAAGCACGTGGTGTGGGTCACTTTTAATGCAACCACGGGCATGAACATGGTACAACGGTGTGGTTTAAGCCCAGAACTAAGGGCCACCCACAACACCACATCGTTAACCCAAACCTGGGCGGACATGGTGGAAGAAGACCAAACCAAAGAGGCAATGCGGGGCGGTGGGCTCACTACTGATGGATtaaccatttacgttggcCGCAACACCAACCACGTCACAGACTTAAAGTCCATGACCACCATTAAAAACAAAGCAGTAGTGGGGGACGAAGAACTATTTGTAGTGTTGCTGCAGAGTGAATGGTTGATGGGCATGGTTGACGAAAACAAATATTACCTGGTGGCCGGCCAAGAACTAGCCACCATGAAAGAGCCCAAAAATGTGACTGATTGGAGGCGCCTGCTGTCTGACCTCACGATCAGCGAGCAGGCACCACCCCGGTTGCTGCAAGCAGCCAGCAATTTCTCCTCCGCAACGATGATTTTCAAAAACAACATGGTGCACCTCTCTGAAGACAGAGCCGTGTTGTATGTGCCAGACACACAAGCAAAACTGGCCGAGTTAGAACAAAAAATAAGAGCCAAAACCAAGCTGGTGCAGATAACAAGAACCCACAAAGAATTAAAACTCCACTTTCTGCTCACCACGGTGGGGACCATAACAATCACTGGTCGGCACAGAATAGAAGTGTTTGGTAACAGGGCGCTCAACTATTTGACGAGTCATGTCGACCAAGTTAATGATGCTCAATTCAAGGCatgGGTTGCACATTTAATAAATAACTTTAGATGCACAGCGTGCGCAGCAGAGATGTACATTAATCCGGTGGCATTAACTGAGCACTTGTCAGATCCAAAAAGTGACACACAATGGACCGGGTGTAGCAGGTGGAGCTGCGAAAATTTTAAAGCCCCGCCAAGCTCCGAAGAAAACTCTATCGAAGACGTAATGGACACAGAATCGTCTTCGTCAGAAGAGGAACCGGACAAGGAACCTGCCAGTGACGGCACTTTGTTAGGCGACGAAGCAACCACAGGCCAAGAGTGTGAGCATGAATCAGACGAAGAATACTATGACACAATTGACACTATTCACATAAACCCTACGACAGTCTGCACCACACCGCGCAGTAGAAGTGGCCAAGACGATATTAACGACGAAATCGTTCAACTAATGTCAACAGCACAGCCAAGTGGGGAGCAAACCGCAGAAAGCCAAACAACAACAACTTCCCGCACACATCACACATCACCACCACCCAACCCCCAACAACAGGAGCGCACACTAAGTAGTTTGGTGGTGTCAACCTTACATAACAACCAACCCAGACGCACGTCAAGAGAGAGGGTGATGCACCTCGTCGAACAATATGCGCAACACACTACCGGGTGGATTATCTTTCCGCGGTGCATCAGCATCAGGCACATGCAAACAGCTCTGTTACATGGCTGCCCAATGCACTGCAGATGGACTTTACAACGGAGCGAAATGTGCAACATTTTCACCCTCATGGCCGGGCTGACCAGGGTGCTCACCATTGCTTTCAATAGAGCCGGCAACGTGGTTGGCCCCATGGATGGAAGTGTAGCGGACGACATGTACATCACGTTGGCAACACATGCTGTGGATCAATGGCGGTTGGTGGCTTCGGGTCAAATGGAGTTGGATGAAGATTTAGCATTGGAGGGGCTAAAATTTATCAACCAATCACGATTGTCACCGATCCATGTGCATAGGCATGACACTATTGTCATCGACGATACCGAATGGCCGGCATGGATCGGCGCCTCCGTGAAGGAATATGAGCCATTCATGACCAGACCAAGATACCAACAATCTGTGACACACCACGCGATAAAGACCATGCTACCAGAGTTGCCCACTGAAGTGTGCGGGTGCGAAACAAAACTTCTTGACGAAAACAAGATATGCGCCACATACCCTTGGTTGGACACGTACACAATGACACCTGATGAACTTGCAAGTTGCATCAGTTGGATTCAATTCGTGCAGTACCGGCAAATTGGGGGTCTGAAAACCCACACAGTTATAGATGGTACAGTAGTAGACAGCACAACCTTTGGGGGTTGTGCACTGTGCGCAGGGCTGAAGATGACAATAAATGAGGAAATTACAATGTTAATCAATAGTAGATCTGGTCCCGAACCCGGAACCATGTGGGTCAACATCAACCATGTAATACAATATCCACACGTGATATCAGAACTGTGCAAAATTCTAGAAATGCCTGGCATGATCATGCATTATCCAGATCTGGAGCTGTATTGGTCCGTCAGGCAACTAACCGAAATGGACGGCATGATACAAGACTTTGGCCAAGCTATGTCGTTAATTGAGCGACTCAGGGCAGGAGTTGATTGGGTGATCGAAAGGGTCACCAGCTTCAGTTTACAATGGAAGTGTGACCAGTATAACACAGTCAATTCGGAGTACGTGACGATGCTGGGAAAAGCACTGTTGGATGCGGGGGTTGATCCATCATTGCACTGTCATGCAATCACTCATACAGGCACACTATTGGTGGGGCTGCCAACACGATGGTCAGCCTCGGGCCAAACCATTGTCATATCAAAATTTAAGGGCAGATTCCACTGCACCAGTCACTCACTGGCCATCACGCAGCGATGGGAGTGGCAAAGCGCTAGTGACATGCTAGTGGAGGCAGTAAAACACCGCATGCTGATACTCTTTGCAAACAAATTGAGTGGCCGCAGAGAGTTGGTTCGGTCCATTTTGGAAGATTCCGTAGCCATCAATCTGGACGAGGTAAGAAGGGCCATGGGAGCAGTTAAGTTGAACTTGAGTTTGGATTACCACAGGGAGCATAACACCGGGGTGAACGCTATGTTAAATGATCGCAAAAACAAACTGGTGCAGGGCCAACTCGTGGGCCCCCACCAGGCAATGTACTTGTCACCAAATCAAATCCGAATGTTTGGCAGAGACATTAATAGAACATGTTGTACGCACAACTTGACAGAACACGGCACATGGCTACCTGACGAAGGGTTATTTGCCACAGCGGAACAGGTGGCAGCGAAAATTATATCTGGTTGGTGTGGGAaAAACAAATTGTGCACATACTCGGGTACATATAGTTACATACCGTTAGTGAACTCCAGCTGGAACATGAAAAGTGTAAAACCATTACGCAGCACACCGCTATACAGTGCGTGTGCACTGGCTATGAATGGGGCATTAAACAATTTGATCACATCTATAAAAAACTTAAAAGACAAAGCAGAAACCGTGACCGGGTTTGCATTGAGTGATAATGAGAAAGCCTTGATAGCCATGTGTGACAAGGTTGCAACCACAAAAACAAACCCTTGGTGCAGCAGCGAGTCAGTCAGTGATGGCGACAAAGCCATGTTGGGAAGTGAGTATTTGGGCCTAGACTGGCATTCATTTTGTGATCAACTGGTAAAAACCAGTGCAAGAGAGACATTCATTTTGGTTCCCGAGCAAGACAATCACAGCGGCGATTACTACAACATCCATGACAGACAGGAGTATTTTGAATGTCAAGGGGTTGCAAGCACCACATTAATAAccctcaaccccaccacatggagCAACCTGCTCATCAAACACAGCATGAGGTACAACAACAAAATAATACACGCAGAACAGCTGATGTCAATATTGGGTTTGCGGCTTGTCAGCATAGTGGTGACCTCCTCAGCAGTGTCAGTGCCTGGTCACTACTCACCATGCATACCAAACAACAGCAATCAAGTGGTCTACAAAATTAATACCCCACAATTGTCATGGAACACATCCGTTTTCAGCCACCACAAGACAACAGTAAAGGTGGACTCCAAAATGTACCGCCACCTAAGCTTCAGAGTTGTGTCCGATACGTGCAGCTTCGAAGATTTATTGTCCTACGCCAGAACGTACATGCAAACCACCACTTATACTCAGACTGGATACTACAGGGAGAATTTAGATCAAATCAATGATTTGCATCTCATTTGCGCGTGCGTTTATCACGAATATCAGCAGAAAAAGACAAGCTGGTGCACACTAGCCGCGAAACATTGTGACTTACCAACCATGGATGTGGGCCGGGTAATGGCACATTTGAACACCAATTTGACCCATGCCGCCTTCAAGCTGATGCAAGCATGGAAGTTAAATGACGGAACTTTGCACGACATTAAATTGGCTTCTAAAAACACGATAGGACCTGAAATGACAGACACAATCAAAAATCTTGCCACAATGAACATTGAGAAGAAAGAGGTGAGCCGGCAAATTATGGTGCAAAACGGAGATCGCGCTTTGCCAGACATCACGACAAATTGTCCATGTGAGAGGCAAATGACCAAATCTAATGATGTGGTGATGCCAATACTGGGCGGATTGGTCAATGATGGATCATTGCTGACAGAGTTTTTGGCGGAAGAACTACGATCAAACTCAAGTTATTGGTGCTTAGTACAATCCAGCACGGATGGTACTAACACAGCCGTACAACTGGGGGACGGAGTCAGGCGTTCTCACAATTTGTCAAGCGTGAACCTCAAAAAgaaacgtAGGTGCAAACACCCCGGCAGTGAAACAAACCTCACGGAGAAGTACATACAGCAAAGGATGGAAGTCACTGCCAATATGAACAGCGCAAACGACACTCAACCACGTGAGTCAGATGGCCCacctatcacaggcacatacagcacaccAACAATGACCCGGTGCAAGACTAGTGGCCATGAACCATCAGTTGACAACTTGCATcaacctgcagcgagtaataatgaggacGGGGACACCACTGAAAGTACTTGGAGACTAATGGAACTATCGCATGTCATCATCAAACGAGTGTCCCAAGCTGTCACCAGCAGGGTGGGCGAGTGCCGGCAAAACAATTGCAAGGCCAGATCTAAGGAAATTAAAAGACTGTTGAAAGATTTAGCTTCAGCAGTGTGCCACAAGTGCAACACCTGGGCAGAGGAGCATTTTGCGGCCAGCAGGGACCATGAAGAGTCTGGGGCCACGGAACCGCTGAATGGAACTACGACACCCGAGCCAACACCGCGCAGCACAAGACCCACAAGCGGAGAAAGAGTGTGGGTGTTGATGTCCCCCACACAACGCGTTGGCATCTTAATGCAAGAATTAAACCAGGCCCATccacatgccgagctctcacaattgagaactgtgactagtgacATGAGTCATGACAACGACAACACGGACAACGAGATACGCGCAAAGGCCGACCAATTGGCAGAGCTAATGGAGAATGGGTTTTCATCAGAATTTGACCGGACCGAcaCCATCAGCTCGTACGACGATACCATCTCGGAATGTGAGGAGCCCACCACGTTTGTTAGACAGCAGTGGCAGCAGTACGATGTGGACCATGtgatcacAGAAGACTTTCATATCCAAAGCGACTTGGAGGAACCACAGTTGACCTCCAGAACCCCAGAGCCAGCGTTGAGCGAGGCACAATCACAGGACGACAAAACCGCATACGCACGCATGTGTGACAAAacccacaagctgttgtgtgatacaacggcTAACGCTGCCGGGTTGCTGGAAGATTATATTGAATCAACAACACTAGAATGGTTGACTACAGAAGCAAGGCAAGCCAACCGTACGTGTTGGTGCCATGTGACCACACCCGAATGTTTACAAGAGTTAGTAGGAATTGACGCATGCCATGTCATTGTCAAACCATATGCTCACATTACAGGTGTGGCCACCAGAACCAAAGAAATATACAATCCCAACACAGAGGGAAACTGCGTGGTGAATAGCCTGAACTATCTTGAGCAACACCACCCCAGAGTAGACAAAGTAATGGAAAGGGCTTGCGGCGTGTACGGGAAAACCATGTGGTTAACGGAGTCCGACGCGGCAGCCATTAGCTGGTTATGCAACCTAGAAACTCGTTATAAACACAACCAAACCGTGCATGAATTGGCGACTGGGTCAAGCGCCTCCCCATTATGTATAAATAGAGTGACAGGGGACGCAGTTGACCATTGCACGGTGCACAGCATCACCGTCAGCCATCGCAGCGAAAAGCCGATGCAAATTAGTATAGTGAGCAGGGCGACAACAACTGAAATAGAGCAGAACATCTGCACCCACTTGGACATCCAAGTCGGGCTCGCAGAATTGTTACACGATTTGAACGAATTAGTAAACTTCCAAGGAGTGGGAGAGTTTATCGCTAAATACAGAAGCAGTTTTTCCACTCAAAGGGAATTAGAGGCAAACTTGCGATTAACAGGCCAAGTGCACACCATCAGGACCAAATCTACACCAGTCATCCCAGGCGAGTGGGTAAATTGCGCCAGGCTAAAATACTTTAAAAACACCAGCGACAAATTGAAACCGGGGGAATGTGTCCTGTGGACAGATGGTGTGCACATGCATTGGGCCGCTGTAATATCTTCAAACAGACACGGCCAGTACATGTGGACACCATGCCCCATGACGACCGCAGTTCCTGTGGTACTACGAATTTCCATGAAGTGGGCCAAACCAGCACTACAGTCTCCTACAGCAACAAACACAATATTAGGCGAGATCCAGCACGTCACAGCACTCAATGAGGCGACCGCACGGCACATGGTATcacacaagctcgtggtGGGTAAACCAACAATCAACAATCCACATGCAACAGTGCTGGTTGTAGCGGATTTTGACAATCGTAAGCACCACAAATTTGACGACAGAACGGTTTTGCGTTCCTGGGATAGAGAAAACATTGTGGTAATCAACAAGTGCCCAGGAAGGGTGCCAACTGAAGTGTTCTTTGAGGAAAGTGACATGATGACAGCAGTGTTATACCAGGGCGACCCTGAGATCCACATAGTGACAGACCACACTTCAACTGCCCATTGGGTCAAGAGCTTTTTGATGAGAAACAAGATAAGATCAACAGTGAAACGTAACGTGGTGGTAATCTCAGATTCTTGGCACACGCTGCAAGAAAAGAAAACGGAGGAGAAGCTTGAACCTCACGTACACAAGATCAATGACATGTGGCACCTAAACAAACTagttCCTGGAACCTTCACCATTGGCACGGAACAGGAGTTGTGCCAATTACTGCAAATTACTGAACCGGACTTAAGTGTGACAGGCCTGTTAGCTAACCTACCGTTGCAGCTGACAATACACACGGCATTCCACGAACCCTTAATGCACACCAGCTACCACCAAAGTCAATTAATAATGGTTCGCAATGACAAATATGTGCGGTCTGGGTTCGTGAACAAAATACAAATCAAGGCAGCCCAACGTGGTGGTTCCTGGATGGAAAtcccaGCAGGAACAACTGCAGGAAAATGGTGGGACCAAGAACAGGACGCACATCAAGTGCCAAACAATGACGTGTCAATAGGCAAGGtggaagccatggtTGGCGCATCCATAACTAGCATTCCATCCACTCAGAACCAAACGTCAACGCACGAAGGAAAAACCGCAATAATTCCTTGGATGGACTACCCCATGTACGTGGACACCAGCGTAGCGGACACACCAACATTGGAATTGGTTCCAGCCCAAATTATGGACATGTGGGACACCACAGATTTGAGCGGGTATTTGGATAGATTTGCACCAGAAAACCAGTTCAGCATCAAAGTGAAAACTGTGCCCACAAAGTTGGCACAGTCAATAAAGAGCACACTAGTCAAATACCCAATATACAGCAGGCCAGTGTATACAAAAATGGCCAACCAAGAATTCAACGCAGTAACAGGAAGGCTTGGTAAAGTGACACAGTACAGAGTGGGGGATTATTGTGCAATTAGTGAGGCAAACGGCATAGCAAACACATTTTTCCAAAAAGACTGGAAACAAGTGGTGGCAGAATACCAAAGCAATACAATAACCTATAATACGGACAAAGTAAAAGAATGGTTGCAAGGCAGACCTAATGTAGAAAGTATAGTTGGGGAGGTTAACAACATTCTGACGGAAGGGTTTATGAAACACCCAATCAACAAGCTTAACGTTCACCTCAAACTGGAATCTTTGTTAAAATCACAACCAGTTGTGGACCATAGGCAAACAAAGGCCAGGATTTTAGTATGGCAAGCAAAaGGCATGTGTGCCATATACAGCCCAGTGTTTAAAATGGCAAAAGATAGGTTAAAATCACTGCTAAATACAAAAACTGTGTACGCAGATGGATTGCGTCCGGACCAATTAGCACTGAGAGTGACGCAAGCGGATCCTGCCAATTACATCATGGAGAACGACCTGGAACAACAGGACCGACAAACCGATGACAAACTGTTGGACGTGGAAATGGCAATTTATCACATGCTGGGTGTGCAGGAAGAACTGTTGTCGTTATGGAGGCAGTGCCATAACAACTGGAACTTTAAGGGAAGGTCATGTCGCGGAGAACGAAACTGGATGAGGCTAACAGGCCAGGCTACCACTGCACTAGGCAACGCAATAACCAATTTGTGCGTGCATTGGCGGCTGTGCAGCAAATTGGGTAATGATTGGAAACTATTTGTGTtattaggaGACGATGGGGCGTTGTTGACCAACAGGACATTGTCAAACAAAGAAGTGAAAACGCATGGTAAATTGTTGTACAATATGATAATGAAACCCAATACACACGACAATGTTGGCACATTCTGCTGTTTCAACATATACAAGTTGCCCACAAACCGATGGACCATGGGCCCCGACATTGCAAGACTGCGAAGGCGGTTTGAAGTGACCAATGGGGCGTCAGAGGCCACCCTTGTCAACCATCACGCAAGGTGCATGTCATATTGTTGTATGTTAGGATCAATATCACCCATTCAACAATTGATCAAGGAGAGGGGTTACACCGTAGAGCCACCAATGTGGTATGACTGGAACTCCAATGCAAGAGCCACAGAGGACAAGTACCACTGGCTACCGGGGAAAAGTGACACAGAGCTGCGCATGCTGctggATATGATGAGACAAACAAACTACATCGATCACAAACTGTTGCACTGGGTGGAAGCTACCCGCTAGTGCTCCTACCCACGTTTTGGTGACGAACAAGATTGCCCCCCCCTc

>BaYMV_RNA1_KX117192

AACAAACACAAACATCCCACGCTCCCCAAGCTTACAATACCTGTAGAGTGATTTTTGTCATCCTACTGTTTGTAAGTTTTTCAGCAAATCAACCCGCTTGCTTTCGCACCTCCTTCACCTTCACTCTCGCTCTTATGGAGCAAACCCTAGCTCAAGCTGTGTCACGGAAAGGTAAAACAAACACACCTATGGCAGAAGAGCGGAAACATTTCAGCCCCATGAATTTTTCCGCAAACTTTGTTGCTCCTGAGCTCTTCTATAGTGCTAATGTGAGGAAAATTAAAAACATCTTCAGAGAGCGCAGCACAACTCGGTTTCTGGACGCAATTTCAAGTGATTTTGAGCTTGTTGCTTTTCTCACCTTGAGTCCCGCTCACTTAATGCAACTTGAGACAACTCTGCGCCAAGAGATACGTTCGTGTGTTGTGCCAATTGTCACTTCTGATGCTAGCTTCGAAACAGTTGCTGTCATCAAAACTGCTCTGGATGGCATGAGATTTCACTTTGGACACACCACTCTTGAAAAAGGGTGGATGTCTATGATGCGCCATGCAGAGAGTTGCCTGCAAGAGAGCTCCTCTTCGGCTGTAAATGATCTGCAGATGCAGATTAAACGTGTTGGATCACTGCTCTTGAGTGGAAAAAATCGCGTAGAAAGCTGCGAACTATCTGTCTTGAACCTCACAGCGCGACGTTTCAGGATCGAATATGGGCTAAATGGTACATACTTTGGCGAACATGTTGCTATGCTACTCGACTTAAAGCGTTATATCTATGGTACCGTGCCAAAAGAGTTTTTGTGGGCGAAAACCAAAAAGCACTCGCTATTCACAACCCCCGAGTGGATCAAGCGTACGCCCATCGACTGTTTCTTGTTTTGTCTTCGCGTTATCCCTATCTTACATAGGTTTGGTGTAGCCATATCACTTCTTTACTGGTCGTGTGTTGCTGCTCTGAATTTTCCTGCTTTTATGGCTTTCTTATTTAAGCGTCAGTTTGCTAAGTATTTAGCTCACTCTTTTGCCAAACACACCATCTACTTTATATTTTTAACTATAATTGCTATTTTATGGTCTTTTCGCACTTTCGCTTCTCAAAAACCAAAAATAGTGCTTCAGGCTAGGAGCACAGCAGAAAAAGAGAAAAAGCTTATGATGATTCTAGCTAGTGTCGTTGGGATTACTTATCTCTTCGATTATGACATAGCAGAAGCTCTTGGGAATTGTCTACATAAAATAAGCCGTCTATCCTCATATCTTCTCGACGACCATCAAGGCATCGCATCACGAATGTTTGGAGCGAGCTATGGGCTTCAGGCTGGCGATAGTGCTGAGGATGCAGTCACAACGATAATCAGCGACCTACTGTCTGTTACTTTTAAAATTGTGGACGAAGACGCATCACAGGGCACCGTGGAGGATGCATCTGAGACAACCTTTCACAGTTGGGTTGGTGTTAACACACTTGCAGGTCGCAATATGTCAAGACCACTCCAGTATGACGTCAATAAAACGTATGCACTCACACCTCAAAATGTCCAGCTACAAGCGCGAGCTATGGCAGACGCAAACAACTGCTGGTCAATGGTTGTTGGTCATACAGGATCTGGAAAGTCAACTTACTTACCTGTGCAGTACTCCAATTACTTATCCACCAAGAGTGATAGGCGACAGCAGATTCTTATCTGCGAACCAACTCAGGCTGCCACTGAGAACGTTTGCGCTGGCGTTGCTGCCAATCTTGGCAGAGCTGTTTACGGGCGCCATGAGGGTTGGTCCAGAATGGGGGATCATTGCATTCAAGTTATGACGTATGGTTCCGCGCTCCAGTGCCATGCTATGGACCCTAGCTTCATATCAACTTTCGACGCAATTTTTCTTGATGAAGCACACGATGTCAAAGAGCACTCTCTAGTCTTTGAGAGCATTTGTGACACATTTAAATCCGTTCGAAAGTTCTATGTTTCAGCTACTCCTAGAGATGGAAGCGTGTGTCCTGAAGCAGCTAGGAAATATCCTTTACATGTTGAAACTAGCGTTTGCGATTCGTATCGGAAGTTCATTGCAGCTCAAGGTGGGGGAGATTTGCTGGACATCTCAAAACATGATACAGCTTTAGTCTTTCTAGCAGGACGGCCTGAGTGTATTAAAGCTGCTAATGCCTGGAATGCTAGTGTCACCGGTGAGAAGCGCGCCTTTCCTTTGTCGAGCGACAACTTTGCTACCGACTTCTCGATGCTCACCGAGAGGCTCAAGACACACAAAACCATCATCTTCACCACGAACATAATTGAAACAGGTGTCACTCTTAGCGTTGATTGTGTTGTTGATTTCGGCCATACGATGCGCCCATGTTTGGATCTCAATCAAAAATCATTACGTCTTGATCGACGCAGAGTGACGCGAAACGAAAGACAGCAGAGAATTGGGCGAGCAGGTCGGCTTAAAGACGGGTACGCCATAGTGTGTGGAGATGTTGATAGAGCTGTAAATGTCATCTCTCCTGATGTTCTTTATGGTGCCGCTCTTCTGAGTTTTAAACACAATGTGCCTTTCTACATGAACGAAACATTTGAGAGTTCCTGGTTGGAGGGTGTTACAAAGGCACAAGCTGATACAATGACAATATTTAAGCTTCCCATATTTCTAACACGAGATCTCATTAATGCAGATGGCTCAGTTGCTAGGGAGTTTCTCGATGTTTTAAAGAAGCATCAATTCACAACAAGTGACATAAAGCAAGCACCAAGTGTCACAGCCAAACATATCTTCCCAACTTGGGCTTCATACTTCTCTCTCCACCAGGCGCTTCATTATGGTGATGACAAGGATGAGATACCACACGAGTTGCGATATGCGCGCGTTCCTTTCTCCGTCACAACTTTGAGCAAATTTGATTGGCCAGCCTTAGCTCTTGCATGCGAGAAACATAGAGCTTCCATGTCAAATGTGTTTGCTGGCATCGAGGAACCCGCACGCGTTGTTACTCTACAAACAAATCCTGCGAACATACAAGCAAGCATCACACATTTAACGCACATGAGTAAGAACTATAAAACTCTTATTGAAAACAACCAGCACGTGCGTCAATCCATGATGACGAATGTTATGTTTAAGTGGTTCTCTTCCACTCGCATCACTAAAGATCTTGATAGGAACTTGCGAAGATGCACGGATAACCTCTCAGTTGTCGAGGCCACGCTTAGCTCACTTAGGCAAATCTTAGCTGGCAACACGCAGGTTCATGCAACGCCGCACATGCAGAGCACTCTTGAGGACATAATTGAACTCCAGGCTAGTGACACACTTACGGAAGAAAGCTTAGCAAACGCACTTGGCATCTTTGTTCCAAAGTGCAACCTCTTTCTGCTGCTAGCTACTAAAGGTTTCAAGCTTGTGTACGTTGTCTGCATCTTACTTCTTGTGAACCTTGTTTATACTGGCCTTCGCAAGTGGCGCGAGCATCTCAAGCAAAAAGGAAGCAACGAAATCCTAACTAACACAATGCCAGTTAGCGAAGGGGGCGAAATTCTCGCTGAGGTTATGAAAATGGAGCCTAAAATGAGAAAGAACATCAAGAGGGATATGGATGCAGCTGTTGAGAGCAAACTATGTGGTTTCACTTTTGTTTTTCCAGATGATGATAAGATTGGTCTTGAAGGCAAGGGGAACAAATACCGCCCTCGCGAAGACGCTCGCCTGATGTACTCCACTAGAGAGGACGCCACCTTTGACGCCTGGAATGAGAAAGCGAAGGAAAGACGCAAGAAGGTAACTGACAAAGCTGAGCCAGAGCTTCGGAGAGCCTACGAGAAGAGACCATACTTCAATTTCTACGACCTTCAAACAGATAGCAACATTCTGGAAGCTATTTTCTACACCACTGAAGGCGATGAGTTTTTCCGAACAGCAGACCCTAATAAGGACATGAACTTGGTTGCTGATAAACTACGCTCCTTTCTCGATACAAAGCTTGTTGTTGGACATCATCAGCGAAAGCTACTAGAAGAAACAGCTGAGGTTGTTATCAAGGATACGAAGGGAACTGCGCACAAGATGAGTATTTCACAGCATGATCCAGATTCTCTGAAGCAGAATGGGTCCGGCAAAGTTGGGTATCCAGAACACCGAGGACAGTTTCGGCAGGAAGGAGTTGCCATCACAGGGGATTACGATCTTGAAGCTGAGTTTGGTGCCGACACCGATGAAATAACGCTTGAGGCCTCTACTGGAATTTTATTGTCACAAGTTGGGGTCGATGTTGCAACTCGAGTTGGGAGAATTTGCATTGGCACTTTCAATATGAACTGTTACTTCTATAGCGACTGGATTCTAGTTCCAGGACACCTGCAAGATAGATCTGGCAACGTGACAATTCAATTCCCTGACCAAACAGTGCAAACCACAACTGACGCACTCAACGCGAATGGTGTGAAACGATTCTATGGATTAGATGTGATAGCAATTCGTCGCCCTGCTATCTTGCGGCCTCGCACCAAGTTGGTCAAAGCTTTTGCTATTGAGGAACCAGTTATCGCACAAATGGTCTTTGTCGATGCACAGGGAGTCCGTAAGTTCACTCAATCCGATTGGGCCAGAAAGGAGGAGAATTCTGGGCGTTGGTCACACAAGATCTCCACTGTTCTTGGTATGTGCGGATGCCCAGTCTTGGACGTTGGAAAGAACAGATTGATAGGGATCCATGTTGCCACAAACTACACAAAGAAGCGCAACGAGTTCCAACCCTTCACTCAGGAGGTTGTCGATTTTATAAATGGACCTGGAACAAAAATCCCCTACTGCCCATGGGTATTTGATAGACCGGCTTGTGGGTATTCATCCCACAACGCTTTGTTCGAGAAACCAACCACACTGGCTGACGTCATTCACATGCAGGCTTCTGATGGTTTGCACAACATTAACAATGCCATTGAGGGTTTTGGGAGTTCACTAAGGGGTCAGCTTGTGTCACCACCTACTGAATCCACCAGGCAACGTTTCGATAAGCTGTTTGGTAGTGGCAGCTTCGAGCTCATTGGGCAGATGAACAAAGGCTTAATCGACAAACACGTGATTGTTGGGGAAAACGATGATGTTTATGACTTCATGCGCGAGCACCCAACATTTACTTGGTTGAAAGATTTCATGAATGAATATGCGCCTAGTGTTTTGTCTTACTCTGCTTACTACAAAGACCTTTGTAAGTATAATCGTGCAAAACACGTGCTCACTTACAACCCAGAAGAGCTCCATTGTGCGACGAAAGGACTGATCAAGATGTTGGAAGACGCTGGCTTGACACAAGGCAGTGTGAGGACACCCCAACAAGTTGTTTCGGACATTCAATGGAACACTTCCGCTGGACCGAGTTATCAAGGGAAGAAACGCGACCTCTGTGCCCACTTGAGTGATGACGAGGTGCTGCATCTTGCTGAGGTGTGCCGTCAACAATTTCTCGAGGGCAAGTCAACTGGAGTGTGGAACGGTTCTCTGAAAGCTGAGTTGAGAACCATTGAGAAAGTTGAGGCGGAGAAAACGCGTGTTTTCACAGCCTCTCCCATAACAAGCCTGTTTGCCATGAAATTCTACGTTGATGACTTTAACAAGAAGTTCTATGCTACCAATCTGAAAGCCCCCCACACTGTTGGCATCAATAAGTTCGGTAGGGGTTGGGAAAAATTGCATGACAAGCTTAATCGCCCTGGATGGTTGCATGGCAGTGGAGATGGTTCTAGGTTCGATAGCTCTATCGATCCTTTCTTCTTCGACGTTGTGAAAACTATTCGTAAGCACTTTCTCCCTTCTGAGCATCATAAAGCCATTGATCTCATATATGATGAGATTCTCAACACAACCATCTGCCTGGCTAATGGGATGGTTATCAGGAAGAATGTTGGAAACAATAGCGGGCAGCCTAGCACTGTTGTTGACAACACACTTGTGCTTATGACTGCATTTCTCTACGCGTACATTCACAAGACAGGGGATCGTGAGTTAGCCTTGCTAAACGAGCGTTTCATCTTCGTTTGCAACGGTGATGACAATAAGTTTGCCATATCTCCGCAGTTTGATGAAGAGTTCGGGCACGATTTTTCCCCAGAACTTGTTGAACTTGGACTTACGTATGAGTTTGATGACATCACTAGCGACATTTGCGAGAATCCTTATATGTCCCTCACCATGGTTAAAACTCCTTTTGGTGTTGGTTTCTCTCTTCCCGTGGAAAGAATCATAGCCATAATGCAGTGGTCCAAGAAAGGGGGGGTTTTGCACTCATATTTAGCTGGCATCTCCGCAATTTACGAATCCTTCAACACACCAAAACTCTTCAAATCAATCTATGCGTACCTCCTTTGGCTCACTGAGGAGCACGAGGCAGAAATTCTTGCAGCCATGACTCAATCCTCAACCGCCCTTCCTATACCATCAATGCTCGATGTTTATCGGTTGCATTATGGTGATGATGAGATTTGGCTGCAAGCAGCTGATCCTCTCACTGATGCGCAAAAAGAAGCTGCACACACTGCCGCAGCTGATAGAGCACGCCTCGACCTAGCCGATGCCGATCGTAGACGTAAAGTTGAAGCGGACCGTGTGGAAGCGGCAAGAGTTAAAAAGGCGGCTGATGCTGTGCTAAAACCAGTTAACCTTACAGCAACACGCATGCCCACAGAAGATGATGGCAAACTCAAAACGCCATCTGGTGCTAGAATACCATCATCAGCTGCAGATGGTAACTGGAGCGTGCCGGCAACGAAACAAGTTAACGCTGGTTTAACACTTAAAATTCCCCTGAATAAGCTCAAAAGCGTGCCTAAGTCTGTTATGGAACATAACAACTCAGTTGCACTTGAATCTGAGCTAAAAGCCTGGACTGATGCTGTGCGCACAAGTTTAGGTATCACAACTGATGAGGCATGGATCGATGCCCTCATACCCTTCATCGGATGGTGCTGCAACAATGGCACCTCAGACAAGCATGCTGAGAATCAAGTAATGCAAATTGACAGCGGAAAGGGAGCTGTCACAGAGATGAGTCTGTCCCCATTTATTGTTCATGCTCGAATGAATGGTGGACTCCGAAGGATCATGCGTAACTACAGTGATGAAACCGTCTTACTCATCACAAACAACAAACTAGTTGCACATTGGTCAATGAAGCATGGCGCTTCTGCAAATGCAAAATATGCTTTCGATTTCTTCGTCCCACGATCATGGATGAACCCACAGGATATCGAAGTATCAAAGCAAGCGCGCTTAGCAGCACTTGGAACTGGAACGTATAACACCATGTTAACTTCCGACACTACCAATCTGCGTAAGACAACCAACCACAGGGTTCTGGACTCAGATGGACACCCAGAACTAACCTAAACCATCACCCCGTGCCTTATACATCACCCACCCCCACTCATAACTCAGTCTTTAAATTATGTTCGTGACGTCTTTGTATCAGGCTCGGAACGGTTCTATGATCCATATGCCTTTGGCAGTTACAATGTCAGCATTCTCATTGAGGAGTATGACAGCGTTTGATCAGCCGTTTTTAATCATCCGTCGTTGN

>BaYMV_RNA1_HYT37_MN107377

AACAAACACAAACATCTCACGTTTCCCAAGCTTACAATACCTGTAGAGTGATTTCTGTCATCCTACTGTCTGTAAGTTTCTCAGCGAATCAACCCGCTTGCTTTCGCACCTCCTTCATCTTCACTCTCGCTCTTATGGAGCAAACCCTAGCTCAAGCTGTGTCACGGAAAGGTAAAACAAACACACCTATGGCAGAAGAACGGAAACATTTCAGCCCCATGAATTTTTCCGCAAACTTTGTTGCCCCTGAGCTCTTCTATAGTGCTAATGTGAGGAAAATTAAAAATATCTTCAGAGAGCGCAGCACAACTCGGTTTCTGGACGCAATTTCAAGTGATTTTGAGCTTGTTGCTTTTCTCACCTTGAGTCCCGCTCACTTAATGCAACTTGAGACAACTCTGCGCCAAGAGATACGTTCGTGTGTTGTGCCAATTGTCACTTCTGATGCTAGCTTCGAAACAGTTGCTGTCATCAAAACTGCTCTGGATGGCATGAGATTTCACTTTGGACACACCACTCTTGAAAAAGGGTGGATGTCTATGATGCGCCATGCAGAGAGTTGCCTGCAAGAGAGCTCCTCTTCAGCTGTGAATGATCTGCAGATGCAGATTAAACGTGTTGGATCGCTGCTTTTGAGTGGAAAAAATCGCGTAGAAAGCTGCGAACTATCTGTCTTGAACCTCACAGCGCGACGTTTCAGGATCGAATATGGGCTAAATGGTACATACTTTGGCGAACATGTTGCTATGCTACTCGACTTAAAGCGTTATATCTATGGTACCGTGCCAAAAGAGTTTTTGTGGGCGAAAACCAAAAAGCACTCGCTATTCACAACCCCCGAGTGGATCAAGCGTACGCCCATCGACTGTTTCTTGTTTTGTCTTCGCGTTATCCCTATCTTACATAGGTTTGGTGTGGCCATATCACTTCTTTACTGGTCGTGTGTTGCCGCTCTGAATTTTCCTGCTTTTATGGCTTTCTTATTTAAGCGTCAGTTTGCTAAGTATTTAGCCCACTCTTTTGCTAAACACTCCATCTACTTTATATTTTTAGCTATAATTGCTATTTTATGGTCTTTTCGCACTTTCGCTTCTCAAAAACCAAAAATAGTGCTCCAGGCTAGGAGCACAGCAGAAAAAGAGAAAAAGCTTATGATGATTCTAGCTAGTGTCGTTGGGATCACTTATCTCTTCGATTATGATATAGCAGAAACTCTCGGGAATTGTCTACATAAAATAAGCCGTCTCTCCTCATATCTTCTCGACGACCATCAAGGCATCGCATCACGAATGTTTGGAGCGAGCTATGGGCTCCAGGCTGGCGATAGTGCTGAGGATGCAGTTACAACGATAATCAGCGACCTACTGTCTGTCACTTTTAAAATTGTGGACGAAGACGCATCACAGGGCACCGTGGAGGATGCATCTGAGACAACTTTTCACAGTTGGGTTGGTGTTAACACACTTGCAGGTCGCAACATGTCAAGACCACTCCAGTATGACGTCAATAAGACGTATGCACTCACACCTCAAAATGTCCAGCTCCAAGCGCGAGCTATGGCAGACGCTAACAACTGCTGGTCAATGGTTGTTGGTCACACAGGATCTGGAAAGTCAACATACTTACCTGTGCAGTACTCCAATTACTTATCCACCAAGAGCGATAGGCGACAGCAGATTCTTATCTGCGAGCCAACCCAGGCTGCCACTGAGAACGTTTGCGCTGGCGTTGCTGCCAATCTTGGCAGAGCTGTTTACGGGCGCCATGAGGGTTGGTCCAGAATGGGGGATCATTGCATTCAAGTTATGACGTATGGTTCCGCGCTCCAGTGCCATGCTATGGACCCTAGCTTCATATCAACTTTCGACGCAATTTTTCTTGATGAAGCACACGACGTCAAAGAGCACTCTCTAGTCTTTGAGAGCATTTGTGACACATTTAAATCCGTTCGAAAGTTCTATGTTTCAGCTACTCCTAGAGATGGAAGTGTATGTCCTGAAGCAGCTAGGAAATATCCTTTACATGTTGAAACCAGCGTTTGCGATTCGTATCGGAAGTTCATTGCAGCTCAGGGTGGGGGAGATTTGCTGGACATCTCAAAACACGACACAGCTTTAGTCTTTCTAGCAGGACGGCCTGAGTGTATTAAAGCTGCTAACGCCTGGAATGCTAGTGTCACTGGTGAGAAGCGCGCCTTTCCTTTGTCGAGCGACAACTTTGCTACCGACTTCTCAATGCTCACCGAGAGGCTCAAAACACACAAAACCATCATCTTCACCACGAACATAATTGAGACAGGTGTCACTCTTAGCGTTGATTGTGTTGTTGATTTCGGCCATACGATGCGCCCATGTTTGGATCTCAATCAAAAATCATTACGTCTTGATCGACGCAGAGTGACGCGAAACGAAAGACAGCAGAGGATTGGGCGAGCAGGTCGGCTTAAAGACGGATACGCCATAGTGTGTGGAGATGTTGATAAAGCTGTAAATGTCATCTCTCCTGATGTTCTTTATGGTGCCGCTCTTCTGAGCTTTAAACATAATGTGCCTTTCTACATGAACGAAACATTTGAGAGTTCCTGGTTGGAGGGTGTCACAAAAGCACAAGCCGACACAATGACAATATTCAAGCTTCCTATATTTTTAACACGAGACCTCATTAATGCAGATGGCTCAGTTGCTAGGGAGTTTCTTGATGTTTTAAAGAAGCATCAATTCACAACAAGTGACATAAAGCAAGCACCAAGTGTCACAGCCAAACATATCTTCCCAACTTGGGCTTCATACTTCTCTCTCCACCAGGCGCTTCATTATGGTGATGACAAGGATGAGATACCACACGAGTTGCGATATGCGCGCGTTCCTTTCTCCGTCACAACTTTGAGCAAATTTGATTGGCCAGCCTTAGCTCTTGCATGCGAGAAACATAGAGCTTCCATGTCAAATGTGTTTGCTGGCATTGAGGAACCCGCACGTGTTGTTACTCTACAAACAAATCCTGCGAACATACAAGCGAGCATCACACATTTAACGCATATGAGTAAGAACTATAAAACTCTTATTGAGAACAACCAGCACGTGCGTCAATCCATGATGACGAATGTCATGTTTAAGTGGTTCTCTTCCACTCGCATCACTAAAGATCTTGATAGGAACTTGCGAAGATGCACGGATAACCTCTCGGTTGTCGAGGCCACGCTTAGCTCACTCAGACAAATCTTAGCTGGCAACACGCAGGTTCATGCAACGCCGCACATGCAGAGCACTCTTGAGGATATTATTGAACTCCAGGCTAGCGACACACTTACGGAAGAAAGCTTAGCAAACGCACTTGGCATCTTTGTTCCAAAGTGCAACCTCTTTCTGCTGCTAGCTACCAAAGGTTTCAAGCTTGTGTACGTTGTCTGCATCTTACTTCTTGTGAACCTTGTTTATACTGGTCTTCGCAAGTGGCGCGAGCATCTCAAGCAAAAAGGAAGTGACGAAATCCTAACTAACACAATGCCAGTTAGCGAAGGGGGCGAAATTCTTGCTGAGGTTATGAAAATGGAGCCTAAAATGAGAAAGAACATCAAGAGGGATATGGATGCAGCTGTTGAGAGCAAACTATGCGGTTTCACCTTTGTTTTTCCAGATGATGATAAAATTGGTCTTGAAGGCAAGGGGAACAAATACCGCCCTCGCGAAGACGCTCGCCTGATGTACTCCACTAGAGAGGACGCCACCTTTGACGCCTGGAATGAGAAAGCGAAGGAAAGACGTAAGAAGGTAACCGACAAAGCTGAGCCAGAGCTTCGGAGAGCCTACGAGAAGAGACCATACTTCAATTTCTACGACCTTCAAACAGATAGCAACATTCTGGAAGCTATTTTCTACACCACTGAAGGCGATGAGTTTTTCCGAACAGCAGACCCTAATAAGGACATGAACTTGGTTGCTGATAAACTACGCTCCTTTCTCGATACAAAGCTTGTTGTTGGACACCATCAGCGGAAGCTACTAGAAGAAACAGCTGCGGTTGTCATTAAGGATACGAAGGGAACTGCGCACAACATGGAAATTTCACAGCATGATCCAGATTTTCTGAAGCAGAATGGGTCCGGCAAAGTTGGGTATCCAGAACACCGAGGACAGTTTCGGCAGGAAGGAGTTGCCACCACAATGGATTACGATCTTGAAGCTGAGTTTAGCGCCGACACCGATGAAATAACGCTTGAGGCCTCTACTGGAATTTTATTGTCACAAGTTGGGGTCGATGTTGCAACTCGAGTTGGGAGAATTTTAATTGGCACTTTCAATATGAACTGTTACTTCTATAGCGACTGGATTCTAGTTCCAGGACACCTGCAAGATAGATCTGGCAACGTAACAATTCAATTCCCTGACCAAACAGTGCAAACCACAACTGACGCACTCAACGCGAATGGTGTGAAACGATTCTATGGATTAGATGTGATAGCAATTCGTCGCCCTGCTGCCTTGCGGCCTCGCACCAAGTTGGTCAAAGCTTTTGCTATTGAGGAACCAGTTATCGCACAAATGGTCTTTGTTGATGCGCAGGGAGTCCGCAAGTTCACTCAATCCGATTGGGCCAGGAAGGAGGAGAATTCTGGGCGTTGGTCACACAAGATCTCTACTGTTCTTGGCATGTGCGGATGCCCAGTCTTGGACGTTGGAAAGAACAGATTGATAGGGATCCATGTTGCCACAAACTACACAAAGAAGCGCAACGAGTTCCAACCCTTCACTCAGGAGGTTGTCGATTTTATAAATGGACCTGGAACAAAGATCCCCTACTGCCCATGGGTATTTGATAGACCGGCTTGTGGGTATTCATCCCACAACGCTCTGTTCGAAAAACCAACCACACTAGCTGACGTCATTCACATGCAGGCTTCTGATGGTTTGCACAACATTAACAATGCCATTGAGGGTTTTGGGAGTTCACTGAGGGGCCAGCTTGTTTCACCACCTACTGAATCCACCAGGCAACGTTTCGATAAGCTGTTTGGTAGTGGCAGCTTCGAGCTCATTGGGCAGATGAACAAAGGTCTAATCGACAAGCATGTGATTGTCGGGGAAAACGATGATGTTTATGACTTCATGCGTGAGCACCCAACATTCACTTGGTTGAAAGATTTCATGAATGAGTATGCGCCTAGTGTTTTGTCTTACTCTGCTTACTACAAGGACCTTTGTAAGTATAATCGCGCAAAACACGTGCTAACTTACAATCCAGAAGAGCTCCATTGTGCGACGAAAGGACTGATCAAGATGTTGGAAGACGCTGGCTTGACACAAGGCAGTGTGAGGACACCCCAACAAGTTGTTTCGGACATTCAATGGAACACTTCCGCTGGACCGAGTTATCAAGGGAAGAAACGCGACCTCTGTGCCCACTTGAGTGATGACGAGGTGCTGCACCTTGCTGAGGTGTGCCGTCAACAATTTCTCGAGGGCAAGTCAACTGGAGTGTGGAACGGTTCTCTGAAAGCTGAGTTGAGAACCATTGAGAAGGTTGAGGCGGAGAAAACGCGTGTTTTCACAGCCTCTCCCATAACAAGCCTGTTTGCCATGAAATTCTACGTTGATGACTTTAACAAGAAGTTCTATGCTACCAATCTGAAAGCCCCCCACACTGTTGGCATCAATAAGTTCGGTAGGGGATGGGAAAAATTGCATGACAAGCTTAATCGCCCTGGTTGGTTGCACGGCAGTGGGGATGGTTCTAGGTTCGATAGCTCTATCGATCCTTTCTTCTTTGACGTTGTGAAAACTATTCGTAAGCACTTTCTCCCTTCTGAGCATCATAAAGCCATTGATCTCATATACGATGAGATTCTCAACACAACCATCTGCCTGGCTAATGGGATGGTTATCAGGAAGAATGTTGGAAACAATAGCGGGCAGCCTAGCACTGTTGTTGACAACACACTTGTGCTTATGACTGCATTTCTCTACGCATACATTCACAAGACAGGGGATCGTGAGTTAGCCTTGCTAAACGAGCGTTTCATCTTTGTCTGCAACGGTGATGACAATAAGTTTGCCATATCTCCGCAGTTTGATGAAGAGTTCGGGCACGATTTTTCCCCAGAACTTGTTGAACTTGGACTTACGTATGAGTTTGATGACATCACTAGCGACATTTGCGAGAATCCTTATATGTCCCTCACCATGGTTAAAACTCCTTTTGGCGTTGGTTTCTCTCTTCCCGTGGAAAGAATCATAGCCATAATGCAGTGGTCCAAGAAAGGGGGGGTTTTGCACTCATATTTAGCTGGCATCTCTGCAATTTACGAATCCTTCAACACACCGAAACTCTTCAAGTCAATCTATGCGTACCTTCTTTGGCTCACTGAGGAGCACGAGGCAGAAATTCTTGCAGCCATGACTCAATCCTCAACCGCCCTTCCTATACCATCAATGCTCGATGTTTATCGGTTACATTATGGTGATGATGAAATTTGGCTGCAAGCAGCTGATCCTCTCACTGATGCGCAAAAGGAAGCTGCACACACTGCCGCAGCTGATAGAGCACGCCTCGAACTAGCCGATGCTGATCGTAGACGTAAAGTTGAAGCGGACCGTGTGGAAGCGGCAAGAGTTAAGAAGGCAGCTGATGCTGTGCTAAAACCAGTTAACCTCACAGCAACACGCATGCCCACAGAAGATGATGGCAAGCTTAAAACGCCATCTGGTGCTAGAATACCATCATCAGCTGCAGATGGTAACTGGAGCGTGCCGGCAACGAAGCAAGTTAACGCTGGTTTAACACTTAAAATTCCCCTGAATAAGCTCAAAAGCGTGCCTAAGTCTGTTATGGAACATAACAACTCAGTTGCACTTGAATCTGAGCTAAAAGCCTGGACTGATGCTGTGCGCACAAGTTTAGGTATCACAACTGATGAGGCATGGATCGATGCCCTCATACCCTTCATCGGATGGTGCTGCAACAATGGCACCTCAGACAAGCATGCTGAGAATCAAGTAATGCAAATTGACAGTGGAAAGGGAGCTGTCACAGAGATGAGTCTGTCCCCATTTATTGTTCATGCTCGAATGAATGGTGGACTCCGAAGGATCATGCGTAACTACAGTGATGAAACCGTCTTACTCATCACAAACAACAAACTAGTTGCACATTGGTCAATGAAGCATGGCGCTTCTGCAAATGCAAAATATGCTTTCGATTTCTTCGTCCCACGATCATGGATGAACCCACAAGACATCGAAGTATCAAAGCAAGCGCGCTTAGCAGCACTTGGAACTGGAACGTATAACACCATGTTAACTTCCGACACTACCAATCTGCGTAAGACAACCAACCACAGGGTTCTGGACTCAGATGGACACCCAGAACTAACCTAAACCATCACCCCGTGCCTTATACATCACCCACCCCCACTCATAATTCAGTCTTTAAATTATGTTCGTGACGTCTTTGTATCAGGCTCGGAACGGTTCTATGATCCATATGCCTTTGGCAGTTACAATGTCAGCATTCTCATTGAGGAGTATGACAGCGTTTGATCAGCCGTTTTTCAATCATCCGTCGTTG

>BaYMV_RNA1_HYT38**_**MN107378

AACAAACACAAACATCGCACGCTCCCCAAGCTTACAATACCTGTAGAGTGATTTCTGTCATCCTACTGTTTGTAAGCTTCTCAGCGAATCAACCCGCTTGCTTTCGCACCTCCTTCATCTTCACTCTCGCTCTTATGGAGCAAACCCTAGCTCAAGCTGTGTCACGGAAAGGTAAAACAAACACACCTATGGCAGAAGAGCGGAAACCTTTCAGCCCCATGAATTTTTCCGCAAACTTTGTTGCCCCTGAGCTCTTCTATAGTGCTAATGTGAGGAAAATTAAAAATATCTTCAGAGAGCGCAGCACAACTCGGTTTCTGGACGCAATTTCAAGTGATTTTGAGCTCGTTGCTTTTCTCACTTTGAGCCCCGCTCACTTAATGCAACTTGAGACAACTCTGCGCCAAGAGATACGTTCGTGTGTTGTGCCAATTGTTACCTCTGATGCTAGCTTCGAAACAGTCGCTGTCATCAAAACTGCTCTGGATGGCATGAGATTTCACTTTGGACACACCACTCTTGAAAAGGGGTGGATGTCTATGATGCGCCATGCAGAGAGTTGCCTGCAAGAGAGCTCCTCTTCAGCTGTGAATGATCTGCAGATGCAGATTAAACGCGTTGGATCACTGCTCTTGAGTGGAAAAAATCGCGTAGAAAGCTGCGAACTATCTGTCTTGAACCTCACAGCGCGACGTTTCAGGATCGAATATGGGCTAAATGGTACATACTTTGGCGAACATGTTGCTATGCTACTCGACTTAAAGCGTTATATCTATGGTACCGTGCCAAAAGAGTTTTTGTGGGCGAAAACCAAAAAGCACTCGCTATTCACAACCCCCGAGTGGATCAAGCGTACGCCCATCGACTGTTTCTTGTTCTGTCTTCGCGTTATCCCTATCTTACATAGGTTTGGTGTGGCCATATCACTCCTTTACTGGTCGTGTGTTGCTGCTCTGAATTTTCCTGCTTTTATGGCTTTCTTATTTAAGCGTCAGTTTGCTAAGTATTTAGCCCACTCTTTTGCTAAACACTCCATCTACTTTATATTTTTAACTATAATTGCTATTTTATGGTCTTTTCGCACTTTTGCTTCTCAAAAACCAAAAATAGTGCTTCAGGCTAGGAGCACAGCAGAAAAAGAGAAAAAGCTTATGATGATTCTAGCTAGTGTCGTTGGGATCACTTATCTCTTCGATTATGACATAGCAGAAACTCTCGGGAATTGTCTACATAAAATAAGCCGTCTATCCTCATATCTTCTCGACGACCATCAAGGCATCGCATCACGAATGTTTGGAGCGAGCTATGGGCTTCAGGCTGGCGATAGTGCTGAGGATGCAGTCACAACGATAATCAGCGACCTACTGTCTGTTACTTTTAAAATTGTGGACGAAGACGCATCACAGGGCACCGTGGAGGATGCATCTGAGACAACCTTTCACAGTTGGGTTGGTGTTAACACACTTGCAGGTCGCAATATGTCAAGACCACTCCAGTATGACGTCAATAAAACGTATGCACTCACACCTCAAAATGTCCAGCTCCAAGCGAGAGCTATGGCAGACGCTAACAACTGCTGGTCAATGGTTGTTGGTCATACAGGATCTGGAAAGTCAACATACTTACCTGTGCAGTACTCCAATTACTTATCCACCAAGAGTGATAGGCGACAGCAGATTCTGATCTGCGAGCCAACTCAGGCTGCCACTGAGAACGTTTGCGCTGGCGTTGCTGCCAATCTTGGCAGAGCTGTTTACGGGCGCCATGAGGGTTGGTCCAGAATGGGGGATCATTGCATTCAAGTTATGACGTATGGTTCCGCGCTCCAGTGCCATGCTATGGACCCTAGCTTCATATCAACTTTCGACGCAATTTTTCTTGATGAAGCACACGACGTCAAAGAGCACTCTCTAGTCTTTGAGAGCATTTGTGACACATTTAAATCCGTTCGAAAGTTCTATGTTTCAGCCACTCCTAGAGATGGAAGTGTCTGTCCTGAAGCAGCCAGGAAATATCCTTTACATGTTGAAACCAGCGTTTGCGATTCGTATCGGAAGTTCATTGCAGCTCAGGGTGGGGGAGATTTGCTGGACATCTCAAAACATGACACAGCTTTAGTCTTTCTAGCAGGACGGCCTGAGTGTATTAAAGCTGCTAACGCCTGGAATGCTAGTGTCACCGGTGAGAAGCGCGCCTTTCCTTTGTCGAGCGACAACTTTGCTACCGACTTCTCAATGCTCACCGAGAGGCTCAAAACACACAAAACCATCATCTTCACCACGAACATAATTGAGACAGGTGTCACTCTTAGCGTTGATTGTGTTGTTGATTTCGGCCATACGATGCGCCCATGTTTGGATCTCAATCAAAAATCATTACGTCTTGATCGACGCAGAGTGACGCGAAACGAAAGACAGCAGAGAATTGGGCGAGCAGGTCGGCTTAAAGACGGGTACGCCATAGTGTGTGGAGATGTTGATAGGGCTGTAAATGTCATCTCTCCTGATGTTCTTTATGGTGCCGCTCTTCTGAGTTTTAAACACAATGTGCCTTTCTACATGAACGAAACATTTGAGAGTTCCTGGTTGGAGGGTGTTACAAAGGCACAAGCTGATACAATGACAATATTCAAGCTTCCTATATTTCTAACACGAGACCTCATTAATGCAGATGGCTCAGTTGCTAGGGAGTTTCTCGATGTTTTAAAGAAGCATCAATTCACAACAAGTGACATAAAGCAAGCACCAAGTGTCACAGCCAAACATATCTTCCCAACTTGGGCTTCATACTTCTCTCTCCATCAGGCGCTTCATTATGGTGATGACAAGGATGAGATACCACACGAGTTGCGATATGCGCGCGTTCCTTTCTCCGTCACAACTTTGAGCAAATTCGATTGGCCAGCCTTAGCTCTTGCATGCGAGAAACATAGAGCTTCCATGTCAAATGTGTTTGCTGGCATTGAGGAACCCGCACGCGTTGTTACTCTACAAACAAATCCTGCGAACATACAAGCGAGCATCACACATTTAACGCATATGAGTAAGAACTATAAAACTCTTATTGAGAACAACCAGCACGTGCGCCAATCCATGATGACGAATGTTATGTTCAAGTGGTTCTCTTCCACTCGCATTACTAAAGATCTTGATAGGAACTTGCGAAGATGCACGGATAACCTCTCAGTTGTCGAGGCCACGCTTAGCTCACTTAGACAAATCTTAGCTGGCAACACGCAGGTTCATGCAACGCCGCACATGCAGAGCACTCTTGAGGATATTATTGAACTCCAGGCTAGCGACACACTCACGGAAGAAAGCTTAGCAAACGCACTTGGCATCTTTGTTCCAAAGTGCAACCTCTTTCTGCTGCTAGCTACTAAAGGTTTCAAGCTTGTGTACGTTGTCTGCATCTTACTTCTTGTGAACCTTGTTTATACTGGTCTTCGTAAGTGGCGCGAGCATCTCAAGCAAAAGGGAAGTAACGAAATCCTAACTAACACAATGCCAGTTAGCGAAGGGGGCGAAATTCTCGCTGAAGTTATGAAAATGGAGCCTAAAATGAGAAAGAACATCAAGAGGGATATGGATGCAGCTGTTGAGAGCAAACTATGTGGCTTCACTTTTGTTTTTCCAGATGATGATAAAATTGGTCTTGAAGGCAAGGGAAACAAATACCGCCCTCGCGAAGACGCTCGCCTGATGTACTCCACTAGAGAGGACGCCACCTTTGACGCCTGGAATGAGAAAGCGAAGGAAAGACGCAAGAAAGTGACCGACAAAGCTGAGCCAGAGCTTCGGAGAGCCTATGAGAAGAGACCATACTTCAATTTCTACGACCTTCAAACAGATAGCAACATTTTGGAAGCTATTTTCTACACCACTGAAGGCGATGAGTTTTTCCGAACAGCAGACCCTAACAGGGACATGAACTTGGTTGCTGATAAACTGCGCTCTTTTCTCGATACAAAGCTTGTTGTTGGACACCATCAGCGGAAGCTACTAGAAGAAACAGCTGCGGTTGTTATCAAGGACACGAAGGGAACTGCGCACCACATGGAAATTTCACAGCATGATCCAGATTTTCTGAAGCAGAATGGGTCCGGCAAAGTTGGGTATCCAGAACACCGAGGACAGTTCCGGCAGGAAGGAGTTGCCGTCACAGGGGATTACGATCTTGAAGCTGAGTTTGGCGCCGACACCGATGAAATAACGCTTGAGGCCTCTACTGGTATTTTATTGTCACAAGTTGGGGTCGATGTTGCAACTCGAGTTGGAAGAATTTGCATTGGCACTTTCAATATGAACTGTTACTTCTATAGCGACTGGATTCTAGTTCCAGGACACCTGCAAGATAGATCTGGCAACGTAACAATTCAATTCCCTGACCAAACAGTGCAAACCACAACCGACGCACTCAACGCGAATGGTGTGAAACGATTCTATGGATTAGATGTAATAGCAATTCGTCGCCCTGCTATCTTGCGGCCTCGCACCAAGTTGGTCAAAGCTTTTGCTATTGAGGAACCAGTTATCGCACAAATGGTCTTTGTTGATGCACAGGGAGTCCGTAAGTTCACTCAATCTGATTGGGCCAGGAAGGAGGAGAATTCTGGGCGTTGGTCACACAAGATCTCCACTGTTCTTGGTATGTGCGGATGCCCAGTCTTGGACGTTGGAAAGAACAGATTGATAGGGATCCATGTTGCTACAAACTACACAAAGAAGCGCAACGAGTTCCAACCCTTCACTCAGGAGGTTGTCGACTTTATAAATGGACCTGGAACAAAAATCCCCTACTGCCCATGGGTATTTGATAGACCGGCTTGTGGGTATTCATCCCACAACGCTTTGTTCGAGAAACCAACCACACTGGCTGACGTCATTCACATGCAGGCTTCTGATGGTTTGCACAACATCAACAATGCCATTGAGGGTTTTGGGAGCTCACTGAAGGGTCAGCTTGTGTCACCACCTACTGAATCCACCAGGCAACGTTTCGATAAGCTGTTTGGTAGTGGCAGCTTTGAGCTCATTGGGCAGATGAACAAAGGCTTAATCGACAAGCACGTGATTGTTGGGGAAAACGATGATGTTTATGACTTCATGCGCGAGCACCCAACATTTACTTGGTTGAAAGATTTCATGAATGAATATGCGCCTAGTGTTTTGTCTTATTCTGCTTACTACAAGGACCTTTGTAAGTATAATCGTGCAAAACACGTGCTAACTTACAATCCAGAAGAGCTCCATTGTGCGACGAAAGGACTGATCAAGATGTTGGAAGACGCTGGCTTGACACAAGGCAGTGTGAGGACACCCCAACAAGTTGTTTCGGACATTCAATGGAACACTTCCGCTGGACCGAGTTATCAAGGGAAGAAACGCGACCTCTGTGCCCACTTGAGCGATGACGAGGTGCTGCATCTTGCTGAGGTTTGTCGTCAACAATTTCTTGAGGGCAAGTCAACTGGAGTGTGGAACGGTTCTCTGAAAGCTGAGTTGAGAACCATTGAGAAAGTTGAGGCGGAGAAAACGCGCGTTTTCACAGCCTCTCCCATAACAAGCCTGTTTGCCATGAAATTCTACGTTGATGACTTTAACAAGAAGTTCTATGCTACCAATCTGAAAGCCCCCCACACTGTTGGCATCAATAAGTTCGGTAGGGGATGGGAAAAATTGCATGACAAGCTTAATCGCCCTGGATGGTTGCACGGCAGTGGGGATGGTTCTAGGTTCGATAGCTCTATCGATCCTTTCTTCTTTGACGTTGTGAAAACTATTCGTAAGCACTTTCTCCCTTCTGAGCATCATAAAGCCATTGATCTCATATATGATGAGATTCTCAACACAACCATCTGCCTGGCTAATGGGATGGTTATCAGGAAGAATGTTGGAAACAATAGCGGGCAGCCTAGTACTGTTGTTGACAACACACTTGTGCTTATGACTGCATTCCTCTACGCATACATTCACAAGACAGGGGATCGTGAGTTAGCCTTGCTAAACGAGCGTTTCATCTTTGTTTGCAACGGTGATGACAATAAGTTTGCCATATCTCCGCAGTTTGATGAAGAGTTCGGGCACGATTTTTCCCCAGAACTTGTTGAACTTGGACTTACGTATGAGTTTGATGACATCACTAGCGACATTTGCGAGAATCCTTATATGTCCCTCACCATGGTTAAAACTCCTTTTGGTGTTGGTTTCTCTCTTCCCGTGGAAAGAATCATAGCCATAATGCAGTGGTCCAAGAAAGGGGGGGTTTTGCACTCATATTTAGCTGGCATCTCTGCAATTTACGAATCCTTCAACACACCAAAACTCTTCAAATCAATCTATGCGTACCTTCTTTGGCTCACTGAGGAGCACGAGGCAGAAATTCTTGCAGCCATGACTCAATCCTCAACCGCCCTTCCTATACCATCAATGCTCGATGTTTATCGGTTGCACTATGGTGATGATGAAATTTGGCTGCAAGCAGCTGATCCTCTCACTGATGCGCAAAAGGAAGCTGCACACACTGCCGCAGCTGATAGAGCACGCCTCGACCTAGCCGACGCTGATCGTAGACGTAAAGTTGAAGCGGACCGTGTGGAAGCGGCAAGAGTTAAGAAGGCAGCTGATGCTGTGCTAAAACCAGTTAACCTCACAGCAACACGCATGCCCACAGAAGATGATGGCAAACTCAAAACGCCATCTGGTGCTAGAATACCATCATCAGCTGCAGATGGTAACTGGAGCGTGCCGGCAACGAAGCAAGTTAACGCTGGTTTAACACTCAAAATTCCCCTGAATAAGCTCAAAAGTGTACCTAAGTCTGTTATGGAACATAACAACTCAGTTGCACTTGAATCTGAGCTAAAAGCCTGGACTGATGCTGTGCGCACAAGTTTAGGTATCACAACTGATGAGGCATGGATCGATGCCCTCATACCCTTCATCGGATGGTGCTGCAACAATGGCACCTCAGACAAGCATGCTGAAAATCAAGTAATGCAAATTGACAGCGGAAAGGGAGCTGTCACAGAGATGAGTCTGTCCCCATTTATTGTTCATGCTCGAATGAATGGTGGACTCCGAAGGATCATGCGTAACTACAGTGATGAAACCGTCTTACTCATCACAAACAACAAACTAGTTGCACATTGGTCAATGAAGCATGGCGCTTCTGCAAATGCAAAATATGCTTTCGATTTCTTCGTCCCACGATCATGGATGAACCCACAGGACATCGAAGTATCAAAGCAAGCGCGCTTAGCAGCACTTGGAACTGGAACGTATAACACCATGTTAACTTCCGACACTACCAATCTGCGTAAGACAACCAACCACAGGGTTCTGGACTCAGATGGACACCCAGAACTAACCTAAACCATCACCCCGTGCCTTATACATCACCCACCCCCACTCATAATTCAGTCTTTAAATTATGTTCGTGACGTCTTTGTATCAGGCTCGGAACGGTTCTATGATCCATATGCCTTTGGCAGTTACAATGTCAGCATTCTCATTGAGGAGTATGACAGCGTTTGATCAGCCGTTTTCAATCATCCGTCGTTGG

>BaYMV_RNA2_MN107379

AACAAACACAAACATCACACGCTCCCCAAGCTCACAATACTTGTAGAGTGATTTCTGTCATCCTACTGTTTGTAAGCTTCCCAGCAAATCAACCCGCTTGCTTTCAAACCCTCCACCATGTCTACTTCCAGCTCACGGTTATTCTTTGACTGTGGCAGTCTTGATTGGCCAAATAAAAGCTTATTTGGTGATCCCACAACACGAGATGTGATGGACGAACACATTTCCAGTACGTGGAATGCGGTTATTAGGAGGCACATGTTGGCGCCCAACGCCGACGCCGAAACCATATTGGGCCGTGATGGTTTACCATCCGCTCAGTTTGACGCTTACGGAGCCATGCTGCCTAGTTTCATTCAAGCTCTAAACGCACCCACAACGCGTCTCCGAATCAGCGCACCGCTGTCCACCGCCGAGTCCATTTTATGCGCCGATGCATCCCACGCTCCTTGGTTGTACATGGCAAACAGTGTGTGCGCATATGAGGCAACCCATTTGCAGCCTGTACAAACTTTTATCGCTTTCAACTTCGCGCATGGTTATTGCTACCTCAGTCTTTTCATACCACTAAGCTTTCGCATCACCCCCGAGAATGCTCGAAGTTTTAATCGGTTCCTTGAGCAGCTTCCCGACATTCTAGGTGCGTACCCAACATTGGCCGCACTGTATAAAACAATGCTATTTGCTGTCAAGCTTTTCCCAGAGGTGCTACAAGCTCCAATCCCCATTATTGCAAAAAGGCCTGGTGTGCTACAATTCCACGTTAGTGATGCTAGAGGGCTACCACCCTCATGGTTCCCCATGAAGTGTGGCAGTGTAGCATCTTTCATAGCGCTTATTACCAACAACCTGAACAGCGATTTGCTTAATGGGATTGTTGGTTCAAACGGTGATGGTGAGCACTACACAAATTGGAACTCTGGGCACGATCACTGGATTGTGCATCGATTTATAACAGTCAAGGATTTGCATAGCGGTTTAAGATCAGCTTTAGAAGTTGACTTGGACACAGAGGGCGGGCGAAACGCCGTTCTCGATCTGCTTCTAGATCTTGGGGTCACTAATCTTGTTCGAAGAGAGAAACGTTTCCCAGCATATCTCCAGGGAGCTGAGAGCGTATATCTGCTTCTATCATGTGAGAGAGTTGGGAACGAGTTAGTTGCTGTGCAAGATGCTCTGCAGGAACCATTGGCTAATTATACTGGAAAGGATTTGAGAGCTCTCATTATCAATCTTGGCGGTTTACCAAGCAGACACCCTGAAATTTGTTACACACGTAACATCTTTGAAAATGATAATCATCTGGTTTGGAATTTTGAGTTTTACCGAATAGCCTCAATTACGAAGAATGCACAAATCGACAGAGATGTCCTGAGCTCATCCATGGCGAACTTATTTAGTGACTTTGTTTCAGAGTCATCAAATGGTGAGTACAGAGTTAAGGAACCGAGGCCTGTTACTCAGTACAGGGTTGAGCATGATGAACCAGTTGCTAGTGGCGCCCCATCTGCTTGGTGGCAAGTTCTTATTGGCATCATCACCGCTATTCTGGGCGCAATAATATTCTTTCTTTGGAGGTGTTTCTTGCGTGCTAAGCGTGTGAAATTCCAGGCAAAAGATTCCTTTCCTTGGTTCACCACGTCTGGTGATGATGACCTCCCGCCTCCCCCTGGTGATTCCCCGTCACGCCCTCCTGGGCGCAGCCCAGATCGAGTTCTCCCGCGTACAGTTGTTCGAGATCTCAGCTTCAATGATGATGATGATTTACACAGTGTTGATCTTAACGAAGCTGGGTCGCGTTTTGGGGAGGTTGTCTCTTTAATTGCAAGGGGAAATCTTCGCGAGCTAGCTGGTGCGATTCCTGAATCTCTTAGCAACTTAACTCTCCTTCAAACGAGCGCAAGTGGATCTGGTTTCTACACTATGGTGGCTCTGTACCTTGCCACTTTAGGGGATGCTATCACTGCATTTCATGAGCATAATGATGCTTCACCTGCCACAATTCAATCACTGCGAACGCTCGAGCTTCAGCTTGAAGCCCGTGGTTTGCGTTTCAATGAAGCTGGCACACCCGCAAATCTCATTCAGAGGGGCGTTAAATCTTCTGTTGGTAGAGCACTTGTGCGACTCACACAGAGTGCTCTTTTAGCCACTGGTGAGAACTTTCGGACACGCATGGCAGCCACACTTGAGAGAATCGCTGCTGAGCGTCTCAACACGTTAACCGCCTATGATCAGCGAGTCATTGAAATGACAACTGAACTCCTAGCAGCAATTAAGACTGCTCTTGAGGTCGAGCGCAGTGAACTCACTCCACATCTCGCAAACGCCGAGGCTCTGCTTCAAGTTTACAATAATCTCTTCAGCACCGATTATGCCTCAGCATCGCTGCTCGCTCTTAGGCGCGAGATGATTCTTAGGAGTGCTGAAGGGCGTGTTGGAGAACAGCCCACTAGTGCTTCTGATGCAGCTAATGAGGAATTAGTCCAACGTTCTATGACAAAGCTGGACAAGGAAATTGAGTTATTCCAAGCACAAATTGATAGCCAGCGTCGCGCTGTTACCATCACTGAAGCATCCAATCTTAGAGAGAATATATTGCAGCCAATCAACACCGTTGCAAATATTGCTATGGCTGGAGCTTTTCTGCGTGGGGGCGCGCGCCATCGCATGCCAGGAATACCAGACGTAGCTGCACCCATGCCAAACCCTTTTCGAGCTTTCTCAGGGAGGGGACACTCACTCACAACAACTCGAGGCGCTGGTCTTTTCCGACGCCCTCGAGTTTAGAGTTTCCATTATAGCAGTATTTACTTTCCGCCGCCCTAGCATAATTTAGATCAACATTATCCATTTCTCCCTCCTTTCGCCATCTGCACACCGCTGCCATTAATATGGTAGAGTTAGTGGTTCATCCCCCGCTACGCTCAACATCTCCATCCTTAGATCACCTGCAAACACACAAAATACCAAAAATACCATTTATCATTCTGTTTCAAACGCCAGCATTTTAAATTACCCGCAATTGCATTATCGTTTTGCCATCAAAGTATGGTTCGTTTCATCAACATCTGCTCGCATGATTGGGTGTATGGGGCCAGTGTTGTTTTGGGTATGGAATTTGATTGATGAGAAATTTATTCTTCTTGCCATGAGTTTTCCAGGGGATAAGCTCGGTGCTACAGCTAGCGGCTGTATCCTTCGCGATGCCCCAAACCACCCAACACCACTGATTCTTAGGAATTAGCCTTTCAAATATCCCAACTGCAATGAGCACGATAGCATTGCATCTTCACTCAAGGTCCGGTTAGAGCCCCTGGCAGGTGTGGGCTACAGTAGCATGTGAATGCTGCTCTGTCACCATCTGTCGTCTGCGGTCTCTATCTAGCACTGCGAGTGAAGCCACGTCTTTAAATCCCCAGCATGTTTACTTTATGTTCTGCATAGAAAACGTCACATCTGTGATACAAAAACATGCAATACGTGCCCATCATTGGTCGCGGGTTGCAGTCTGAC

>BaYMV_RNA2_HYT37_MN107381

AACAAACACAAACATCTCACGCTCCTCAAGCTTACAATACCTGTAGAGTGATTTCTGTCATCCTACTGTTTGTTAGCTTTCCAGCAAATCAACCCGCTTGCTTTCAAACCCTCCACCATGTCAACTTCCAGCTCACGGTTATTCTTTGACTGTGGCAGTCTCGATTGGCCAAATAAAAGCTTATTTGGCGATCCCACAACACGAGATGTCATGGACGACCACATTTCCAGTACGTGGAATGCGGTTATTAGGAGGCACATGTTGGCGCCCAACGCCGACGCCGAAACCATATTGGGCCGTGATGGTTTACCATCTGCTCAATTTGACGCTTACGGAGCCATGCTACCGAGCTTCATCCAAGCTCTAAACGCACCCACGACGCGTCTTCGAATCAGTGCACCGCTGTCCACCGCCGAGTCCATTTTGTGCGCTGACGCATCCCACGCTCCTTGGTTGTACATGGCAAACAGCGTGTGCGCATATGATGCAACTCATTTGCAGCCTGTACAAACTTTTATCGCTTTCAACTTTGCGCATGGTTATTGCTACCTAAGCCTTTTCATACCACTAAGCTTTCGCATTATCCCCGAGAATGCTCGAAGTTTCAGTCGGTTCCTTGAGCAGCTTCCCGACATTTTAGGTGCGTATCCAACATTGGCCGCACTGTATAAAACAATGCTATTTGCTGTGAAGCTTTTCCCAGAGGTACTACAAGCTCCAATCCCCATTATTGCAAAAAGGCCTGGTGTGCTACAATTCCACGTTAGTGATGCTAGAGGGCTACCACCCTCATGGTTCCCCATGAAGTGTGGCAGTGTAGCATCTTTCATAGCGCTTATTACCAACAACCTGAACAGCGATTTGCTTAATGGGATTGTTGGTTCAAACGGTGATGGTGAGCACTACACAAACTGGAACTCTGGTCATGATCACTGGATTGTGAATCGATTTATAACAGTCAAGGATTTGCATAGTGGTTTAAAATCAGCTTTGGAAGTTGATTTGGACACAGAGGGTGGGCGAAACGCTGTTCTCGACTTGCTTCTAGATCTTGGGGTCACTAATCTTGTTCGGAGAGAGAAACGTTTCCCAGCATATTTCCAGGGAGCTGAAAGTGTATATCTGCTTCTATCATGTGAGAGAGTTGGAAATGAGTTAGTGGCTGTGCAAGATGCCCTGCAGGAACCATTGGCTAATTACACTGGAAAGGATTTGAGAGCTCTCATCATCAATCTTGGCGGTTTACCAAGCAGACACCCTGAAATTTGTTACACACGCAACATCTTTGAGAATGATAATCACCTGGTTTGGAACTTTGAGTTCTACCGAATAGCCTCAATCACGAAGAATGCACAAATCGACAGAGATGCCCTGAGCTCATTCATGGCGAACTTATTTAGTGACTTTGTTTCAGAGTCATCAAATGGTGAGTACAGAGTTAAGGAACCAAGACCTGTTACCCAGTACAGGGTTGAGCATGATGAACCAGTTGCTAGTGGTGCCCCATCTGCCTGGTGGCAAGTGCTTGTTGGCATCACCACCGCTATTCTAGGCGCAATTATATTCTTTCTCTGGAGGTGTTTCTTGCGTGCTAAGCGCGTGAAATTCCAGGCAAAGGATTCCTTTCCATGGTTTACCACTTCTGGTGATGATGACCTTCCGCCCCCCCCCGGTGACTCCCCGTCTCGCCCTCCTGGGCGTAGTCCAGATCGAGTCCTTCCACATGCAGTTGTTCGAGATCTCAGCTTCAATGATGATGATGATTTACACAGTGTTGATCTTAACGAAGCTGGGTCGCGTTTTGGGGAAGTTGTCTCTTTAATTGCAAGGGGAAATCTCCGCGAGCTAGCTGGTGCGATTCCCGAATCTCTTAGCAACTTAACTCTTCTTCAAACGAGTGCAAGTGGATCTGGTTTTTACACTATGGTGGCTCTGTACCTTGCCACTTTAGGGGATGCTATCACCGCATTTCATGAGCATAATGATGCTTCACCTGCCACAATTCAATCACTGCGAACGCTCGAGCTTCAGCTCGAAGCCCGTGGTTTACGTTTCAATGAAGCTGGCACACCTGCAAATCTCATTCAGAGGGGCGTTAAATCTTCTGTTGGTAGAGCTCTTGTGCGACTTACACAAAGTGCTCTCCTAGCCACTGGTGAGAACTTTCGAACACGCATGGCAGCTACACTCGAAAGAATTGCTGCTGAGCGCCTCAACACGCTAACCGCCTATGATCAGCGAGTCATCGAGATGACAACTGAACTTCTAGCAGCAATTAAGACTGCTCTTGAAGTCGAGCGCAGCGAACTCACCCCACATCTCGCAAACGCCGAGGCTCTGCTCCAAGTCTACAATAATCTCTTCAGCACCGATTATGCCTCAGCGTCGCTACTTGCTCTTAGGCGCGAAATGATTCTCAGGAGTGCTGAGGGGCGTGTTGGAGAACAGCCCACTAGTGCTTCTGATGCTGCTAATGAGGAATTAGTCCAACGTTCTATGACAAAGTTGGATAAGGAGATAGAGTTATTCCAAGCACAAATTGATAGCCAGCGTCGCGCTGTCACCATCACTGAAGCATCCAATCTTAGAGAGAATATATTGCAGCCAATCAACACTGTCGCAAATATTGCTATGGCTGGAGCTTTTCTGCGTGGGGGCGCGCGCCATCGCATGCCAGGGATGCCAGATGTGGCTGCACCCATGCCAAACCCTTTTCGAGCTTTCTCAGGGAGGGGACATTCACTCACTACAACTCGAGGTGCTGGTCTTTTCCGACGCCCTCGAGTTTAGAATTTCCATTATAGCAGTATCTACTTTCCGCCGCCCTAGCATAATTTAGATCAACACTATCCATTTCTCCCTCCCTTCGCCATCTGCACACCGCTGCCATTAATATGGTAGAGTTAGTGGTTCATCCCCCGCTACGCTCAACGTCTCCATCCTTAGACCACCTGCAAACACACAAAATACCAAAAATACCATTTATCATTCTGTCTCAAACGCCAGCATTTTAAATTACCCGCAATTGCATTATCGTTCTGCCATCAAAGTATGGTTCGTTTCATCAACATCTGCTCGCATGATTGGGTGTATGGGGCCAGTGTTGTTTTGGGTATGGAATTTGATTGATGAGAAATTGATTCTTCTCACCATGAGTTTTTCAGAGGATAAGTTCGGTGCTACAGCTAGCCGCTGTATCCTTCGCGATGCCTCACACCACAAAACACCACTGATTCTTAGGAATTAGCCTTTCAAATATCCCAACTGCAATGAGCACGATAGCATTGCATCTTCACTCAAGGTCCGGTTAGAGGCCCTGGCAGGTGTGGGCTACAGTAGCATGTGAATGCTGCTCTGTCACCATCTGTCGTCTGCGGTCTCTATCTAGCACTACGAGTGAAGCCACGTCTTTAAATCCCCAGCATGTTTACTTTATGTTCTGCATAGAAAACGTCACATCTGTGTTACAAAAACATGCAATACGTGCCCATCATCGGTCGCGGGTTGCAGTCTGAC

>BaYMV_RNA2_HYT38_MN107380

AACAAACACAAACATCGCACGCTCCCCAAGCTTACAATACCTGTAGAGTGATTTCTGTCATCCTACTGTTTGTAAGCTTCCCAGCAAATCAACCCGCTTGCTTTCAAACCCTCCACCATGTCTACTTCCAGCTCACGGTTATTCTTTGACTGTGGCAGTCTTGATTGGCCAAATAAAAGCTTATTTGGCGATCCCACAACACGAGATGTGATGGACGAACACATTTCCAGCACGTGGAATGCGGTTATTAGGAGGCACATGTTGGCGCCCAACGCCGACGCCGAAACCATATTGGGCCGTGATGGTTTACCATCCGCTCAGTTTGACGCTTATGGAGCCATGCTACCTAGTTTCATTCAAGCTCTAAACGCACCCACAACGCGTCTTCGAATCAGCGCACCGCTGTCCACCGCCGAGTCCATTTTATGCGCCGATGCATCCCACGCTCCTTGGTTGTACATGGCAAACAGTGTGTGCGCATATGAGGCAACTCATTTGCAGCCTGTACAAACTTTTATCGCTTTCAACTTCGCGCATGGTTATTGCTACCTCAGTCTTTTCATACCACTAAGCTTTCGCATCACCCCCGAGAATGCTCGAAGTTTTAGTCGGTTCCTTGAGCAGCTTCCCGACATTCTAGGTGCATACCCAACATTGGCCGCACTGTATAAAACAATGCTATTTGCTGTTAGGCTTTTCCCAGAGGTGCTACAAGCTCCAATCCCCATTATTGCAAAAAGGCCTGGTGTGCTACAATTCCACGTTAGTGATGCTAGAGGGCTACCACCATCATGGTTCCCCATGAAGTGTGGCAGTGTAGCATCTTTCATAGCGCTTATTACCAACAACCTGAACAGCGATTTGCTTAATGGGATTGTTGGTTCAAACGGTGATGGTGAGCACTACACAAATTGGAACTCTGGGCACGATCACTGGATTGTGCATCGATTTATAACAGTCAAGGATTTGCATAGCGGTTTAAAATCAGCTTTGGAAGTTGACTTGGACACAGAGGGCGGGCGAAACGCCGTTCTCGATTTGCTTCTAGATCTTGGGGTCACTAATCTTGTTCGAAGAGAGAAACGTTTTCCAGCATATTTCCAGGGAGCTGAGAGCGTATATCTGCTTCTATCATGTGAGAGAGTTGGGAACGAGTTAGTTGCTGTGCAAGATGCTCTGCAGGAACCATTAGCTAATTATACTGGAAAGGATTTGAGAGCTCTCATTATCAATCTTGGCGGTTTACCAAGCAGACACCCTGAAATTTGTTACACACGTAACATCTTTGAAAATGATAATCATCTGGTTTGGAATTTTGAGTTTTACCGAATAGCCTCAATTACGAAGAATGCACAAATCGACAGAGATGTCCTGAGCTCATCCATGGCGAACTTATTTAGTGACTTTGTTTCAGAGTCATCAAATGGTGAGTACAGAGTTAAGGAACCAAGGCCTGTCACTCAGTACAGGGTTGAGCATGATGAACCAGTTGCTAGTGGCGCTCCATCTGCTTGGTGGCAAGTTCTTATTGGCATCACCACCGCTATTCTGGGCGCAATAATATTCTTTCTTTGGAGGTGTTTCTTGCGTGCTAAGCGTGTGAAATTCCAGGCGAAGGATTCCTTTCCATGGTTCACCACGTCTGGTGATGATGACCTCCCGCCTCCCCCTGGTGATTCCCCGTCACGCCCTCCTGGGCGCAGCCCAGATCGAGTTCTCCCACGTACAGTTGTTCGAGATCTCAGCTTCAATGATGATGATGATTTACACAGTGTTGATCTTAACGAAGCTGGGTCGCGTTTTGGGGAGGTTGTCTCTTTAATTGCAAGGGGAAATCTTCGCGAGCTAGCTGGTGCGATTCCTGAATCTCTTAGCAACTTAACTCTCCTTCAAACGAGTGCAAGTGGATCTGGTTTCTACACTATGGTGGCTCTGTACCTTGCCACTTTAGGGGATGCTATCACTGCATTTCATGAGCATAATGATGCTTCACCTGCTACAATCCAATCACTGCGAACGCTCGAGCTTCAGCTTGAAGCCCGTGGTCTGCGTTTCAATGAAGCTGGCACACCCGCAAATCTCATTCAGAGGGGCGTTAAATCTTCTGTCGGTAGAGCACTTGTGCGACTCACACAAAGTGCTCTTTTAGCCACTGGTGAGAACTTTCGGACACGCATGGCAGCCACACTTGAAAGAATTGCTGCTGAGCGCCTCAACACGTTAACCGCCTATGATCAGCGAGTCATTGAAATGACAACTGAACTCCTAGCAGCAATTAAAACTGCTCTTGAGGTCGAGCGCAGTGAGCTCACTCCACATCTCGCAAACGCCGAGGCTCTGCTTCAAGTTTACAATAATCTCTTTAGCACCGATTATGCCTCAGCATCGCTGCTTGCTCTTAGGCGCGAGATGATTCTTAGGAGTGCTGAAGGGCGTGTTGGAGAACAGCCCACTAGTGCTTCTGATGCAGCTAATGAGGAATTAGTCCAACGTTCTATGACAAAGTTGGATAAGGAGATTGAGTTATTCCAAGCACAAATTGATAGCCAGCGTCGCGCTGTTACCATCACTGAAGCATCCAATCTTAGAGAGAATATATTGCAGCCAATCAACACCGTCGCAAATATTGCTATGGCTGGAGCTTTTCTGCGTGGGGGCGCGCGCCATCGCATGCCAGGGATACCAGACGTGGCCGCACCCATGCCAAACCCTTTTCGAGCTTTCTCAGGGAGGGGACATTCACTCACAACAACTCGAGGTGCTGGTCTTTTCCGACGCCCTCGAGTTTAGAGTTTCCATTATAGCAGTATTTACTTTCATCCGCCCTAGCATAATTTAGATCAACATTATCCATTTCTCCCTCCTTTCGCCATCTACACACCGCTGCCATTAATATGGTAGAGTTAGTGGTTCATCCCCCGCTACGCTCAACGTCTCCATCCTTAGATCACCTGCAAACACACAAAATACCAAAAATACCATTTATCATTCTGTCTCAAACGCCAGCATTTTAAATTACCCGCAATTGCATTATCGTTTTACCATCAAAGTATGGTTCGTTTCATCAACATCTGCTCGCATGATTGGGTGTATGGGGCCAGTGTTGTTTTGGGTATGGAATTTGATTGATGAGAAATTGATTCTTCTTGCCATGAGTTTTCCAGGGGATAAGTTCGGTGCTACAGCTAGCGGCTGTATCCTTCGCGATGCCCCACACCACAAAACACCACTGATTCTTCGGAATTAGCCTTTCAAATATCCCAACTGCAATGAGCACGATAGCATTGCATCTTCACTCAAGGTCCGGTTAGAGTCCCTGGCAGGTGTGGGCTACAGGAGCATGTGAATGCTGCTCTGTCACCATCTGTCGTCTGCGGTCTCTATCTAGCACTGCGAGTGAAGCCACGTCTTTAAATCCCCAGCATGTTTACTTTATGTTCTGCATAGAAAACGTCACATCTGTGTTACAAAAACATGCAATACGTGCCCATCATCGGTCGCGGGTTGCAGTCTGAC

>JSBWMV_RNA1_MN123252

CTCGGGATAATAAATGAAGAAGAAGTAGTCAGGGCGGCGATAGCTACTAGCGCCACTCGGACTAATTCTGAGCTTCATAGAACTGTTTGTGAGCAGATTCGTGAGCAGTTCTTGGATACAGTAGAAAATCAGAAACAAAAAAAAAAAATAGATGTGAGGAGGGATTTAACTCAGGAACAGCTGCAAACGTTGAACGAGTTATATCCAGAGAGACATATTGTTACGAGTGGTTGTGAGAGGGGTACGCATAGCTTTGCTGCGGCTTCGAGGAAGATTGAGACAGATTTACTATTGAGCAGGATGCCAAAGAATGCTAATGTTTATGATATAGGCGGTAATTGGGCCACTCATTTAAAAAGGAAGGATACGCGGAAAGTTCATTGTTGCTGTCCGGTGCTCGATTTTCGTGATGCGCAGCGTAAGACGACTAGATGGCTTTCAGTGGAAAAGTTCTTATCTGAAAAAGATAGCATTTCTGAGCAATGTGGGCAGAAAGTGTTAGAGATACAGGAAGATGAAGATCGGATCTCTAATAACTTGAGAAAAGGTGCTGTGGCACCGGAAGATTTAACGGGTCGTTGGTACTGTGAGAACAGATTCGAGGATTGTGTGTACAGGGCGGAAAAGGCTTATGGCATGGCTATTCATAGTATCTATGATATATCCTTGGACGATTTGGTAAATGCAATGGAAGAGAAAAGGATTAAGTACCTGGTAGGTACTTTTCTTTTTTCGGTCGAGCTTTTTCTTGGGAAGAAGCGAGGAGAATTGACAACTGTGGATGGTTTCTTTGAAATTGATGGTGGTAACGTCAAGTATGGGTTTTACGATGACACGAATTGCGGCTATAGGCACGACTTGCAGCAACTTATGGAGTATTTGACCAAAACCTTCGTTAAGGCGAAAGGTGGTTCAGTTTTCTATCTTGAGTTAACTGAGCAGCGAGGTGACGTGATGTTCTTCTCGCTGACTGATGCTACAGAAGCTAGGATGCATGGTGTTGTCGAGGACGAATCGTTCAAGTGTATACCCATAGAGAGCAAAGACAGTGTTGTTTTTCCTTTGTTTGAACTGAACAAGAAATCTGACGAATTGGAATTTACTGAGGTCTTATTGCCGAAATCTTTTGTGAGGCGCACTATAGAATACACAGCTCGTTTGAAAACCAATCAGTTAAATCCCGAGACGGTGAACAGCTACTTGACTTCGACCAATAATACAGTTATTATTGGTGGATCTGCTAAGAAGACATTGGAGAAAGTTGATGCGATGCTTATACCTCAAATCACTACGACTCTGATAGTGTGGACAGAACTGATGAACGCTAGGCAGAAGAAGGTGCTTGACAGGTTGAGAATTCAAATGAAAGATGATGTGGGTTTCATGGCCTTGGCTCATACTTCTTTCGCGAAAATGTTCGGGAAAGTGAGTTTCTACCAGAGAGCTCTGAGATCTTATGCTAATTGGATATCTTATTGTCATGGGACAGATGCTATTGAATTCAAGAATGTGCCTTTGTATGTTGAGGTTAAAGATCGTGTGAAGATGTGGAAACAGTATGCTCCGAATCATGGGTTTTCTTTCGATCTGGAGGACTTGGATGAAAAAATACAATTATACAAGGAAACTGAGAGAGAAAGGAAAGCGATAAGCGAGTTTGTCGTTTCTGATAAACTGGGTGATTTGTCGGCTGCTGTATGCGAGAACGAAGAACAGAAGGTGTTAGCAGAATATCGTGATCGTCGTCGGAAAGTTGTGGCTGCCGATCTGCGAAATGGTGAGGTGTTCACTAATTTTGTGGACGACTGGTGCAACAAGGAAGATCATTTTAACCATGTGCATGTGGAAGTTGAAAATAAGTATTCTCTTTTGGTCAAAATTGCCATAGCGGTTTGGGAAGCTATAGTACCACCTATGCAATTTGCACCAGTTTATATTGATGATGAAGAAAGAGACACTACAGATGCCGAGAGCATGGTTGAATTGGAGCTTTCTGAGAACGGGGATGAGCAAGATAAGGTTGATGATGTCGAAGACGTTGCTAAACCGTTCAGCGGCGAGCAGACAGAGAAGATGACAGCTCGTGTGGAGAAGGGAAAAGAGAAAGTGGAGGAAGAAGTAGTTGAGGTCAGACGTGGCAGCGGATCTTCGTCCTCTAATTCGGATGCATCCAGGGAGGATGAGCCGGTTCGCGGAGAGGTCCTTTTCGGTAAGCCGTATAAGGCTTGGTATTCTTCTTCAACTTCGTGTGATAGTCAGCAGTCCGAAGAGTCATCTGCAGGACAGATTGATTTTAGGGTTCTGAAGGCTGTTAGCGATGCTATTGAAGAATTAGAGAAGAGGATAGAAGAGGACGTCGCGGCTAATACTGAAGCTGCTAGGAAGGAATGTAACACTCTTGAAGTCTCGAACGTTGGAGAACTGTCATTGAAACCTACTGCGGAGGTCTGTGACCAGACGGTTGTGGTTGATACGACAGACTCAGAAAGTGATGAGGAGCGAGCGAAATCTTGGGGGTCGATGGCTGAGGAAGAGAGTGACGATACTTTTTACATGAATAACATGCTTATTTCAAATAAGGTGCAGAGAAGTTCGTTGCCTAAGCAGCCTGATTTCAACAAGTATAAAACAGTTCAGCAGAAGGCTAAGCAGGAGTATTTGTGGTATCTGCGCTGTAAAATGATTTCTGACAGGACTACTCTTAGGAGTGTCATCGACGATCATTTAAACGGGCTGTATCATAATGGGAACTGCGATCTACCGAAGAATTCTTGTTTTTTAGACTACACTAAAAGTGTCGGAGGAGAGTGGCTGTATGGTGAGCCACTAAAAACTGGGCATTGTTATGGTGTGGGTTTTTCTCTGAATTCTAAAGGTAAGGTCAGTAAGTGTGAATTGTTGAAACTAATGTGGGACGTTGACGAGAGAGGACGCCCTTCGCAGAAGCCATTCAACACAAAGATGTTTCAGTTTGTGTTACTGAACGATCTTACGTTTCTGATGAACGAAATGATTATATTCAGGAATCTTCAAGATACTCTGCAACGTAAGGAGCGTTCTAAACAAGCGAGTATTGTGCTTAAAGACGGTGTGCCTGGATGTGGTAAATCAACGTGGATTCTGAACAACGCCAACTACATAAAAGATGTAGTTATTTCTGTTGGCAAAGAGGCGAAAGAAGACTTGAAAGAAAAATTCATGAAGAAATACAAATGCACTGAAAGTGAGCTAAATAGGATAAGGACAGTGGATTCGTATTTGATGCACGATTGCGGTAAGAAATTGAGGGCATCTACAGTGCACTTTGATGAGGCCCTTATGACTCACGCCGGCGCAGTGTATTTTTGTGCCGATCTACTAGGGGCGAGGAAGGTTATTTGTCAAGGAGATTCGCAGCAAATACCTTTCGTGAATAGGGTAGAATCTATCAAGTTGCAATTCGCAAAACTGGTTATTGATAAAACAGATCTCATACGTATGACTTACCGATCACCCATTGATGTGGTACACTACCTTAACTATAAGTCATTTTATAATGGTGGAAGAATAACGACTAAGAATGAGGTGGTGCGTTCAATGAGTGTTGTTGGGCCTAGGAATGCGAGACCGATGACTTCGGTCTATTCTGTGCCGTATGTGCCTGGTGCACAGTACTTGACATTCACACAAACGGAAAAGGATGATTTGTTTAAGGCTTTGAGGTCGAAAGGACAAGTTAATGTTAACACTGTGCACGAAACTCAAGGGAAGACTTTTGACGACGTTATACTTGTCCGTTTGAAGACTACGGAAAATGAGATATATCCAGGTGGACGCAATTCCAAACCTTACACTATTGTAGGTTTGACGAGGCACAGGAGATCTTTGGTTTACTACACTGCTATTGAGGACAGGCTTTTCTTTGATTTGGTAGAAATGCGCAGTGTAATGGAGGATAAGCTACTGAAGAATCTTAATGTGGAGAATGGGAAATGACGGTTTGGGTCGAAGTATGAATCAATTCTGACCTGTGATCGTGAAGTGAAAGTTCCAGATACAGGTGATTTAGTTACCATTCAGGACTTTTACGATCGAGTTTTTCCAGGTAATTCAACAGTAGATTCTTACTTCGACGGGTACGAAGTGGCAACGACTGATATCAGTATTGAAGTTGAAAACTGTACCGTCAATCCGCATAAGAACATTAAGGTGTGGCAAGAGAAACAAGGTCTGGTTCCTGTCATGCGTACTGCTATGCCTGAGAAGAGGCAGAATGGTATGGTTGAATCACTACTCGCTCTGAAGAAGAGGAATATGGCTGCACCTAAACTGCAAGAGGCAGTTAATGAGTTTGAAGTGATTGAACGCACGATTGACCGTGCGAAGAACATTTTTCTCAATGAGGATCTGATTGATACTACAGCGGCATCTACTGTGGAGTCCAATATGAGATGGTGGGACAAGCAATCGCACACTGCAAAGAAACAGCTGTTGAGTGAGACAAAGATGCTGCATGAGATAGATGTTTGCACTTACAATTTCATGATCAAAAACGACGTGAAGCCGAAAATGGATTTGACACCTCAGTCCGAATACGCAGCTTTGCAGACCGTTGTGTACCCAGATAAGATTGTAAATGCGCTGTTTGGCCCTGTGATGAAGGAGATCAACGAGAGGATTCGTTATGCTTTGAAGCCACATGTCGTTTACAATTCCAGGATGAATGCGGAGGAATTAAATCGGACTGTCGAATTTCTCGATCCAGAAGAAGAGTATCAGTCTTTCGAAATTGATTTTTCAAAATTTGATAAATCAAAAACTTCGTTACACATTCGAACAGTGATTGAATTCTATAAGCTGTTCGGTCTGGAGGAGATGTTGGCTTTCTTGTGGGAGAAATCGCAATGTCAGACCACTGTAAAAGATCGTCTTAATGGTATAACGGCTTATTTGTTATATCAACAGAAGTCTGGTAATTGTGATACGTATGGGTCGAACACTTGGTCCGCTGCGCTAGCTCTGCTTGAGACTATGCCTTTGGAGAAGGCAAAGTTTATGATTTTTGGAGGAGATGATTCACTCATCTTATTCTCAAAACAGCTTTGCATCGAGGATCCATGTAGAAGATTGGCTTCTTTATGGAATTTTGACTGCAAACTGTTCGACTTTCAGCATAATATGTTCTGCGGGAAATTTTTGCTGAAGATTGGGGATAAGTTCAAATTTGCGCCTGATCCTTTGAAATTAATGACTAAGTTGGGACGCAAAGATATAAAAGACGGGGCATTGCTTTCTGAAATTTTCGTATCTATTGGAGACAATTACAGAGCATACCGCGATTATAGGGTTATTGAGCAACTGGCTCCCGCTGTGCGGGAACGATACAGGACAGGTGAGGATCCAACCGCTGCTTTGATTGCATTGAAGAAATATATTTTCAATTTTGAGATGTTCGCTAAAGCGTTTAATTATAATGGAGGGTTTGTGGTGAGTAAGGTGACTAGAGACTTTGAATGGTAATCGAGGTCTTGGTTGTTTTTTCTTACTTCTTCTCTCCATCGTATTAAACATGACATCTAAAGATGTTTCGAGCGAATCTTACGAGACTGCCTACAATGCAATGTTGGACGCCCAGGACGCAATGGGTCTGACTGCTAATGATGTTAAGAATCATAGACAGAGGAGTTTTAATGTTGTGAACAAGTATGTAGAGAAGGCATTACTCCAAAAAGATGCGGCGACGAAGATGAGAGATGTGTGGACCCGGTTTACTAAGAGTAACAAAGAAGAGGGTACACCGTACAATATTTCTTACAGTTGTGTGATGCTCAACATCATACCGACGGTACCAAGTACTTATGGTGGTACTGTAGAAGTTTCACTGATGGATTCGGGTTTAGAATGGGGTAGTCATATACTGCCAGATCAAACTCAGATGATGGAGTTGGGAAAGGGACCGCAGTTGATGTGCTTCTTTATGCACTATAGCATACCAATTAATGATGAGGACAGAGTGATGAAACTAGCGTTCACGATTGATTGTCAAATGGCTTCGCCGAAAATGAGCGTTATGAATGTTTACACGTACTGGACGCAACGGCAAGGCTATCTTTCTCTGTATTCTGAACCGCAGCGTTCCACTATTTCGAAGTTGGTCGTAGGTTACGATGATAACACGAAATTGAAAACGCGTGACGATGTTCGGCGTTTTGCCTCTAGGTCTTTGGTTCTGAACAACATGAAACAACACGTGCCAAAAATGCTTCCTGAGCAAATTAACCTGCTGAAAGAAAATGTTCCGGTGAACCGTAAGGAGTCTACTATAGATCTCACTAAAGTGGAGCGTGAGAAACACGAAAAGATGGAACAGTTACGGAAACATAAGGCTGCGGCGACCGAGCGTAGTAAAAAGGAAATGCAGCAAAGGGAGAATCAGCTGCAGGTTGAACAGAAGCGTAGAGAGGCGGCTGATCGCTTAGATGAAAAAGCGAAATTGCACAATCACCGTGAGAATCAGAAGGACCCTGAGGCTACAGTGTGATTATTGCTTATTTATCATTGTATATTCTGTATACATATATGGTGTGAGCGTGTATATTTTCGTATGTCGGTTTGTATACATATTGTATTATTACTCTGCATATGTTCTACTTTTGAGTGGTACTTTTTCGCACGTAAAATGCAAAATTTAGTCTAGTGTACTGGGCAGAAGTACACCGTTGTTGATACGATAATCAACACTGATGGTAAATCAGATTCTATTTACCAGAAGATATTCATTCTTCTCAAAAAGAACGTGGTCTTCACGATAGAAGATGGTGCTTGTTAGTTCACCTAAATCGAACTAACGACGGGGCGTTCCGCAGTACGTTTAAACTGCGGGCTTTCCTCTAGTTTCTACTGATGTAATTTTAAAAAGATCAGTATTTGACTATGATGAGGAGCGACTAGGGGTCGCGGTTGCTAAGacacagcaatcaagaggggtgcaaatccccccctgaaccggagggatatccggccc

>JSBWMV_RNA1_HYT38_MN123253

CTCGGGATAATAAATGAAGAAGAAGTAGTCAGGGCGGCGATAGCTACTAGCGCCACTCGGACTAATTCTGAGCTTCATAGAACTGTTTGTGAGCAGATTCGTGAGCAGTTCTTGGATACAGTAGAAAATCAGAAACAAAAAAAAAAAATAGATGTGAGGAGGGATTTAACTCAGGAACAGCTGCAAACGTTGAACGAGTTATATCCAGAGAGACATATTGTTACGAGTGGTTGTGAGAGgggTACGCATAGCTTTGCTGCGGCTTCGAGGAAGATTGAGACAGATTTACTATTGAGCAGGATGCCAAAGAATGCTAATGTTTATGATATAGGCGGTAATTGGGCCACTCATTTAAAAAGGAAGGATACGCGGAAAGTTCATTGTTGCTGTCCGGTGCTCGATTTTCGTGATGCGCAGCGTAAGACGACTAGATGGCTTTCAGTGGAAAAGTTCTTATCTGAAAAAGATAGCATTTCTGAGCAATGTGGGCAGAAAGTGTTGGAGATACAGGAAGATGAAGATCGGATCTCTAATAACTTGAGAAAAGGTGCTGTGGCACCGGAAGATTTAACAGGTCGTTGGTACTGTGAGAACAGATTCGAGGATTGTGTGTACAGGGCGGAAAAGGCTTATGGCATGGCTATTCATAGTATCTATGATATATCCTTGGACGATTTGGTAAATGCAATGGAAGAGAAAAGGATTAAGTACCTGGTAGGTACTTTTCTTTTTTCGGTCGAGCTTTTTCTTGGGAAGAAGCGAGGAGAATTGACAACTGTGGATGGTTTCTTTGAAATTGATGGTGGTAACGTCAAGTATGGGTTTTACGATGACACGAATTGCGGCTATAGGCACGACTTGCAGCAACTTATGGAGTATTTGACCAAAACCTTCGTTAAGGCGAAAGGTGGTTCAGTTTTCTATCTTGAGTTAACTGAGCAGCGAGGTGACGTGATGTTCTTCTCGCTGACTGATGCTACAGAAGCTAGGATGCATGGTGTTGTCGAGGACGAATCGTTCAAGTGTATACCCATAGAGAGCAAAGACAGTGTTGTTTTTCCTTTGTTTGAACTGAACAAGAAATCTGACGAATTGGAATTTACTGAGGTCTTATTGCCGAAATCTTTTGTGAGGCGCACTATAGAATACACAGCTCGTTTAAAAACCAATCAGTTAAATCCCGAGACGGTGAACAGCTACTTGACTTCGACCAATAATACAGTTATTATTGGTGGATCTGCTAAGAAGACATTGGAGAAAGTTGATGCGATGCTTATACCTCAAATCACTACGACTCTGATAGTGTGGACAGAACTGATGAACGCTAGGCAGAAGAAGGTGCTTGACAGGTTGAGAATTCAAATGAAAGATGATGTAGGTTTCATGGCCTTGGCTCATACTTCTTTTGCGAAAATGTTCGGGAAAGTGAGTTTCTACCAGAGAGCTCTGAGATCTTATGCTAATTGGATATCTTATTGTCATGGGACAGATGCTATTGAATTCAAGAATGTGCCTTTGTATGTTGAGGTTAAAGATCGCGTGAAGATGTGGAAACAGTATGCTCCGAATCATGGGTTTTCTTTCGATCTGGAGGACTTGGATGAAAAAATACAATTATACAAGGAAACTGAGAGAGAAAGGAAGGCGATAAGCGAGTTTGTCGTTTCTGATAAACTGGGTGATTTGTCGGCTGCTGTATGCGAGAACGAAGAACAGAAGGTGTTAGCAGAATATCGTGATCGTCGTCGGAAAGTTGTGGCTGCCGATCTGCGAAATGGTGAGGTGTTCACTAATTTTGTGGACGACTGGTGCAACAAGGAAGATCATTTTAACCATGTGCATGTGGAAGTTGAAAATAAGTATTCTCTTTTGGTCAAAATTGCCATAGCGGTTTGGGAAGCTATAGTACCACCTATGCAATTTGCACCAGTTTATATTGATGATGAAGAAAGAGACACTACAGATGCCGAGAGCATGGTTGAATTGGAGCTTTCTGAGAACGGGGATGAGCATGATAAGGTTGATGATGTCGAAGACGTTGCTAAACCGTTCAGCGGCGAGCAGACAGAGAAGATGACAGCTCGTGTGGAGAAGGGAAAAGAGAAAGTGGAGGAAGAAGTAGTTGAGGTCAGACGTGGCAGCGGATCTTCGTCCTCTAATTCGGATGCATCCAGGGAGGATGAGCCGGTTCGCGGAGAGGTCCTTTTCGGTAAGCCGTATAAGGCTTGGTATTCTTCTTCAACTTCGTGTGATAGTCAGCAGTCCGAAGAGTCATCTGCAGGACAGATTGATTTTAGGGTTCTGAAGGCTGTTAGCGATGCTATTGAAGAATTAGAGAAGAGGATAGAAGAGGACGTCGCGGCTACTACTGAAGCTGCTAGGAAGGAATGTAACACTCTTGAAGTCTCGAACGTTGGAGAACTGTCATTGAAACCTACTGCGGAGGTCTGTGACCAGACGGTTGTGGTTGATACGACAGACTCAGAAAGTGATGAGGAGCGAGCGAAATCTTGGGGGTCGATGGCTGAGGAAGAGAGTGACGATACTTTTTACATGAATAACATGCTTATTTCAAATAAGGTGCAGAGAAGTTCGTTGCCTAAGCAGCCTGATTTCAACAAGTATAAAACAGTTCAGCAGAAGGCTAAGCAGGAGTATTTGTGGTATCTGCGCTGTAAAATGATTTCTGACAGGACTACTCTTAGGAGTGTCATCGACGATCATTTAAACGGGCTATATCATAATGGGAACTGCGATCTACCGAAGAATTCTTGTTTTTTAGACTACACTAAGAGTGTCGGAGGAGAGTGGCTGTATGGTGAGCCACTAAAAACTGGGCATTGTTATGGTGTGGGTTTTTCTCTGAATTCTAAAGGTAAGGTCAGTAAGTGTGAATTGTTGAAACTAATGTGGGACGTTGACGAGAGAGGACGCCCTTCGCAGAAGCCATTCAACACAAAGATGTTTCAGTTTGTGTTACTGAACGATCTTACGTTTCTGATGAACGAAATGATTATATTCAGGAATCTTCAAGATACTCTGCAACGTAAGGAGCGGTCTAAACAAGCGAGTATTGTGCTTAAAGACGGTGTGCCTGGATGTGGTAAATCAACGTGGATTCTGAACAACGCCAACTACATAAAAGATGTAGTTATTTCTGTTGGCAAAGAGGCGAAAGAAGACTTGAAAGAAAAATTCATGAAGAAATACAAATGCACTGAAAGTGAGCTAAATAGGATAAGGACAGTGGATTCGTATTTGATGCACGATTGCGGTAAGAAATTGAGGGCATCTACAGTGCACTTTGATGAGGCCCTTATGACTCACGCCGGCGCGGTGTATTTTTGTGCCGATCTACTAGGGGCGAGGAAGGTTATTTGTCAAGGAGATTCGCAGCAAATACCTTTCGTGAATAGGGTAGAATCTATCAAGTTGCAATTCGCAAAACTGGTTATTGATAAAACAGATCTCATACGTATGACTTACCGATCACCCATTGATGTGGTACACTACCTTAACTATAAGTCATTTTATAATGGTGGAAGAATAACGACTAAGAATGAGGTGGTGCGTTCAATGAGTGTTGTTGGGCCTAGGAATGCGAGACCGATGACTTCGGTCTATTCTGTGCCGTATGTGCCTGGTGCACAGTACTTGACATTCACACAAACGGAAAAGGATGATTTGTTTAAGGCTTTGAGGTCGAAAGGACAAGTTAATGTTAACACCGTGCACGAAACTCAAGGGAAGACTTTTGACGACGTTATACTTGTCCGTTTGAAGACTACGGAAAATGAGATATATCCAGGTGGACGCAATTCCAAACCTTACACTATTGTAGGTTTGACGAGGCACAGGAGATCTTTGGTTTACTACACTGCTATTGAGGACAGGCTTTTCTTTGATTTGGTAGAAATGCGCAGTGTAATGGAGGATAAGCTACTGAAGAATCTTAATGTGGAGAATGGGAAATGACGGTTTGGGTCGAAGTATGAATCAATTCTGACCTGTGATCGTGAAGTGAAAGTTCCAGATACAGGTGATTTAGTTACCATTCAGGACTTTTACGATCGAGTTTTTCCAGGTAATTCAACAGTAGATTCTTACTTCGACGGGTACGAAGTGGCAACGACTGATATCAGTATTGAAGTTGAAAACTGTACCGTCAATCCGCATAAGAACATTAAGGTGTGGCAAGAGAAACAAGGTCTGGTTCCTGTCATGCGTACTGCTATGCCTGAGAAGAGGCAGAATGGTATGGTTGAATCACTACTCGCTCTGAAGAAGAGGAATATGGCTGCACCTAAACTGCAAGAGGCAGTTAATGAGTTTGAAGTGATTGAACGCACGATTGACCGTGCGAAGAACATCTTTCTCAATGAGGATCTGATTGATACTACAGCGGCATCTACTGTGGAGTCCAATATGAGATGGTGGGACAAGCAATCGCACACTGCAAAGAAACAGCTGTTGAGTGAGACAAAGATGCTGCATGAGATAGATGTTTGCACTTACAATTTCATGATCAAAAACGACGTGAAGCCGAAAATGGATTTGACACCTCAGTCTGAATATGCAGCTTTGCAGACCGTTGTGTACCCAGATAAGATTGTAAATGCGCTGTTTGGCCCTGTGATGAAGGAGATCAACGAGAGGATTCGTTATGCTTTGAAGCCACATGTCGTTTACAATTCCAGGATGAATGCGGAGGAATTAAATCGGACTGTCGAATTTCTTGATCCAGAAGAAGAGTATCAGTCTTTCGAAATTGATTTTTcaaaatttgataaATCAAAAACTTCGTTACACATTCGAACAGTGATTGAATTCTATAAGCTGTTCGGTCTGGAGGAGATGTTGGCTTTCTTGTGGGAGAAATCGCAATGTCAGACCACTGTAAAAGATCGTCTTAATGGTATAACGGCTTATTTGTTATATCAACAGAAGTCTGGTAATTGTGATACGTATGGGTCGAACACTTGGTCCGCTGCGCTAGCTCTGCTTGAGACTATGCCTTTGGAGAAGGCAAAGTTTATGATTTTTGGAGGAGATGATTCACTCATCTTATTCTCAAAACAGCTTTGCATCGAGGATCCATGTAGAAGATTGGCTTCTTTATGGAATTTTGACTGCAAACTGTTCGACTTTCAGCATAATATGTTCTGCGGGAAATTTTTGCTGAAGATTGGGGATAAGTTCAAATTTGCGCCTGATCCTTTGAAATTAATGACTAAGTTGGGACGCAAAGATATAAAAGACGGGGCATTGCTTTCTGAAATTTTCGTATCTATTGGAGACAATTACAGAGCATACCGCGATTATAGGGTTATTGAGCAACTGGCTCCCGCTGTGCGGGAACGATACAGGACAGGTGAGGATCCAACCGCTGCTTTGATTGCATTGAAGAAATATATTTTCAATTTTGAGATGTTCGCTAAAGCGTTTAATTATAATGGAGgGTTTGTGGTGAGTAAGGTGACTAGAGACTTTGAATGGTAATCGAGGTCTTGGTTGTTTTTTCTTACTTCTTCTCTCCATCGTATTAAACATGACATCTAAAGATGTTTCGAGCGAATCTTACGAGACTGCCTACAATGCAATGTTGGACGCCCAGGACGCAATGGGCCTGACTGCTAATGATGTTAAGAATCATAGACAGAGGAGTTTTAATGTTGTGAACAAGTATGTAGAGAAGGCATTACTCCAAAAAGATGCGGCGACGAAGATGAGAGATGTGTGGACCCGGTTTACTAAGAGTAACAAAGAAGAGGGTACACCGTACAATATTTCTTACAGTTGTGTGATGCTCAACATCATACCGACGGTACCAAGTACTTATGGTGGTACTGTAGAAGTTTCACTGATGGATTCGGGTTTAGAATGGGGTAGTCATATACTGCCAGATCAAACTCAGATGATGGAGTTGGGAAAGGGACCGCAGTTGATGTGCTTCTTTATGCACTATAGCATACCAATTAATGATGAGGACAGAGTGATGAAACTAGCGTTCACGATTGATTGTCAAATGGCTTCGCCGAAAATGAGCGTTATGAATGTTTACACGTACTGGACGCAACGGCAAGGCTATCTTTCTCTGTATTCTGAACCGCAGCGTTCCACTATTTCGAAGTTGGTCGTAGGTTACGATGATAACACGAAATTGAAAACGCGTGACGATGTTCGGCGTTTTGCCTCTAGGTCTTTGGTTCTGAACAACATGAAACAACACGTGCCAAAAATGCTTCCTGAGCAAATTAACCTGCTGAAAGAAAATGTTCCGGTGAACCGTAAGGAGTCTACTATAGATCTCACTAAAGTGGAGCGTGAGAAACACGAAAAGATGGAACAGTTACGGAAACATAAGGCTGCGGCGACCGAGCGTAGTAAAAGGGAAATGCAGCAAAGGGAGAATCAGCTGCAGGTTGAACAGAAGCGTAGAGAGGCGGCTGATCGCTTAGATGAAAAAGCGAAATTGCACAATCACCGTGTGAATCAGCAAGGACCTGAGGCTACAGTGTGATTATTGCTTATTTATCATTGTATATTCTGTATACATATATGgtgtgaGCGTGTATATTTTCGTATGTCGGTTTGTATACATATTGTATTATTACTCTGCATATGTTCTACTTTTGAGTGGTACTTTTTCGCACGTAAAATGCAAAATTTAGTCTAGTGTACTGGGCAGAAGTACACCGTTGTTGATACGATAATCAACGCTGATGGTAAATCAGATTCTATTTACCAGAAGATATTCattcttctcaaaaagAACGTGGTCTTCGCGATAGAAGATGGTGCTTGTTATTTCACCTAAATCGAACTAACGACAGGGCGTTCCACAGTACGTTTAAACTGTGGGCTTTCCTCTAGTTTCTACTGATGTAATTTTAAAAAGATCAATATTCGACTATGATGAGGCGCGACTAGGGGTCGCGGTTGCTAAGACACAGCAATCAAGAGGGGTGcAATTCCCCCCCCGAACCGGAGGGATATCCGgccc

>JSBWMV_RNA2_KU236377

GTATTATTATCACATTACGTTGAGTGTTAACTCTTCTTGTGAAATTACCTTTCTTACCAGGATAAGAAATAAAATATTTTCTGGGTTGTCGTTGTTTGGATACTGAGCAGAGATTGACATCTCGCTATCCTAGGCCCGCATTCTTGCGATAAATCCTAGGTAGTCAGCTGTTAGCGTGCGACGTTGAGAAGCATCTGCATTTCGACTCGCTGGATGAGTTTACTAAACTTAAAGGTGAGGTCGATAGGTCACTACAGAAGGCGGCGGATAAGGTAACTGCGGAAAGCAGTGATTTGTTGCGACCAAAGGTGGTTGTACAGTTACCTTCAATGGCTGTAAAGAATGGTTACACAGGTTATAACAAGGAGTTGAATGTGATGGCTTCTATTCATCCTTTCATCAGGTTGAGTACTTTAATAAGTCAGATTGAAGGATGGCAGGCAACACGAGCGAGTATACTTACCCATTTAGGTGTAGTACTCAATGGAGTTAGTAAGTTGGGTGAGCGAAATTTTCTCTCTAGACAAAAGAGGTTTGGAACGCATACTCAAGACGGTGATGAGATATTCTGTGATCTTGGTGGTGAAGCAGTTATGCAGACTATCGCCAGACTCACGGTAGCACTCCAATCCGCAAGAGGAGAGGGTTCGCAGACCAGGAATGCAAAGAGAGGAACAGCACCAACTAATAACCAGGTTGAGGGTGAAGAGCAAGGTCAGACAGATCAGACGTTGGCCATTTCAAATGCGCTGGCAGAACTTATGGCCTTTATCCACACTAAAGATTTCACGATGAATGAGTGTTATACACAGAGCTCTTTTGAGGCTAAATTTAATCTGAAGTGGGAAGGTACCAGTTGACGGGACGGCGTTTCGGGGAAACTGAGAACACAGATTGTCGAAGATTTCCAGAACAGGCTGGTGTTGGCTGACGATTTGGGTGTTTTTCCGAGACGCAATCTCGACGGAGAGGTTATACGTGATGAGCCCGAAATTATATATGCGGAACTTGAGGATAAGCTGTTAAGTTTTGAGAGTTCCGTGTATCATCCTAAGGCTGTGACGGTGGAAACTGAAGTGGCTGCTTTACCGAAAATGCAGATGGATCTTGAGTTAGGTGTCGTTGCAGATGATGTTGAAATGACTGTGGTAACTGAGGTGCTCATTGATGAGGAGAGCAGTTACTTTTGGTTATATCTTGTGTTCCTTCTGCTGTCGACTTTCTTATCGTGCTTTATTGTATTATCTCGATCCTTTATTCATTTTCTTGGAAATAGAAGCGGTCGGGGTGGTCCCTTCTGCCGGAAGGTTGCTATGTTACTATGGGGAGCGCGGTTGTGGTTGTTGCGCAGGGCAACTAGGCGTTACGGCTCTCTTGGAATCGTCAGGAAAAGGTATACGCGCGCTGGTATGCGCAAGTATTTGCGTGATTACAATTTTCAACTTGACGAGTGGGAGAGTGCGGTTGATTGCTCTCCAGGTGAAGCTTTGATCGTTGCAGATCAAAGAATGTTCGTTAGAGCGGTGAACAAAATTGTGGATCTGGAAAGTGATTTTGATACTGTCGAAAGTGTCAAGAGAGTATTTCGAAACTACAATGTGCGCAATTATAAGGTTGAGAAGTGGTTAGACAAACATACGATACGTTCGGCGCTTGTTGTGCTGAATAGTGTCGTAAAAGATGACAATCTTACCTTGGGCGAAGGCATGCTGTCATTGGAGAGAGCGAGTTTTGACGACGTGTTAGGTTGTGACGTCGAGGCTCAGCGGAACGGCATGTACTTCAAGGACATTATTGCTCTTCAGTTTGCTTTTCGGCTTGTAGGCACACCGGAATTCTCCGATTTCGTGATAGCTTACAAAGGTAATCTGTATCCATGCTATGTTGATGCAATCAAGAGACATAGTTTGATTCTGGAAAAGCATCATGTTGATGAAGATCAGAAAAAGAGAAACGAAAAAGCGCTGGAGTGGCTGCTCATGGCAGGACAGGGTGCTTTTGTGTTGTCTGCTGTTGTATCGACAGGCTTCGCGGCTGTGAAGATTCATAAGTGGATTAAAAGTCGCATGCTCCTGAGGGCGCTGCAGATGTTGCCATCTGTAGGAGGCGGAGGTAACGATGGTGGTGGTGGTTTCTCAGAAGAAGCTCTTCAAAGGTTTGCAGTTGGGCGAAACTTTGAAGAGCGTCTGGCGGCGCTGCACACTGGTTTAGATATGGCAGCTGAGAATCTAGAGAGTTTTTCGCCGGAAGATATGCAAGTTGAGATCCAGCACGTTATTCAGGCTCACGTTGAGGGTTCTTACCATGCTGCGGATCTTCCGTTTTTAGGCAATGTGCCTAGTGTGGCTTCTGTTGTGGCGGACTCCGTTATTGATTCAAGTAGTATTGGCAGTGATGTGTGTAGTGCAGACACGTCTATTTCGTTAGGCGTGGCTAGTGGCGCTAGGCGTCGTGCTGTTGCTAGTAACACGAGGATGTATCCAAGTGCTGGTGTTGTGAGTTACGGTAATTTTAAATCACCGCGAGTTAGTGGTAAAAGTGGAATTGGTAGTATGAAGATGAAAATCGCTAGTAGGCCTATGGCGTCCTAGCGTGCTGTTTTTCATTCTACTCATTTTCATTTTTGTTACGTGTTCGCGTTTCGATTTAAAAGTACTATGTCTGCCTGTGCTTTTCATTCGTGTGACAAGTGTGTCGATGGGCCCAAGAGTGTTGTGTGCGTTAGTAAGTATCGTCACAGTGTTTATAAGGTTTTGGGTCTCTCTGTTGTTAAGTGTCGTTTGCCTGCTGACTGCGGTGTCAATTGCGGTATGCCTGCGGCGTTCGTTCTTGAAAATGGACATCCTAGGTTGACTCTCGATGGATACTGCGGTGAGAAGCACAAAGGTTATGTGATTTCTGGTGCGTGGCGCCATGCGCAACTCCGGACTTTGAACGAAGAACTCGACAAATTGGAAAAGAGGGAGGAGTTTCTAAAAACTCAGATTAAGTTGCTCAGTGATAGCGCTAAGGCCAACACTGCTCCTGTTTACATTCCTAAGAAGATTAATCGTATGAAGGCGGAGGTTTGGGATGTTAATGATAAAATTCTGGATCGTAGCGCTGCTCTTGCAGACGTTATGGATGCTGTTGCGCTGGATTTATCGCCCGATAGTTCACCAAGGAAGTCTAAACCTCTGTGATGTGTGTTTTACTTTACTACTGTATGTTTGTACACTGGAGTAGGTAACCAGGATAAAGAACCCTAGCGAAAATGTTTTGAAAAAATATTCTGTATGTATATAAATAAATGGGCAAGTCTGTTGCTTTGGCTATAGAGTAATCATACTCTTAAAACAATGACGTGGTCTTCGCGATAGAAGATGGTGCTTGTTATTTCACCTAAATCGAAATAACGAAAGGGCGTTCCACAGTACGTTTAAACTGTGGGTTTCAGCCTCTAGTTGCTATTGATGTAATATAAAAAAGATCAATATTCGACTATGATGAGGCGCGACTGGGTGTCGCGGCTGCTTTTACACAGCAGTTAGAAGGGGTTCAATTCCCCCCCCGAACCGGAGGGTTATCCGGCCCA

>JSBWMV_RNA2_HYT38_MN123254

gtattatTATCACATTACGTTGAGTGTTAACTCTTCTTGTGAAATTACCTTTCTTACCAGGATAAGAAATAAAATATTTTCTGGGTTGTCGTTGTTTGGAAACTGAGTAGAGATTGACATCTCGCTATCCTAGGCCCGCATTCTTGCGATAAATCCTAGGTAGTCAGCTGTTAGCGTACGACGTTGAGAAGCATTGGCATTTCGACTCGCTGGATAAGTTTACTAAACTTAAAGGTGAGGTCGATAGGTCACTACAGAAAGCGGCGGATAAGGTAACTGCGGAAAGCAGTGATTTGTTGCGACCAAAGGTGGTTGTACAGTTACCTTCAATGGCTGTAAAGAATGGTTACACAGGTTATAACAAGGAGTTGAATGTGATGGCTTCTATTCATCCTTTCATCAGGTTGAGTACTTTAATAAGTCAGATTGAAGGATGGCAGGCAACACGAGCGAGTATACTTACCCATTTAGGTGTAGTGCTCAATGGAGTTAGTAAGTTGGGTGAGCGAAATTTTTTCTCTAGACAAAAGAGGTTTGGAACGCATACTCAAGACGGTGATGAGATATTCTGTGATCTTGGTGGTGAAGCAGTTATGCAGACTATCGCCAGACTCACGGTAGCACTCCAATCCGCAAGAGGAGAGGGTTCGCAGACCAGGAATGCAAAGAGAGGAACAGCACCAACTAATAACCAGGTTGAGGGTGAAGAGCAAGGTCAGACAGATCAGACGTTGGCCATTTCAAATGCGCTGGCAGAACTTATGGCCTTTATCCACACTAAAGATTTCACGATGAATGAGTGTTATACACAGAGCTCTTTTGAGGCTAAATTTAATCTGAAGTGGGAAGGTACCAGTTGACGGGACGGCGTTTCGGGGAAACTGAGAACCCAGATTGTCGAAGACTTCCAGAACAGGCTGGTGTTGGCTGACGATTTAGGTGTTTTTCCGAGACGCAATCTCGACGGAGAGGTTATACGTGATGAGCCCGAAATTATATATGCGGAACTTGAGGATAAGCTGTTAAGTTTTGAGAGTTCCGTGTATCATCCTAAGGCTGTGACGGTGGAAACTGAAGTGGCTACTATACCGAAAATGCAGATGGATCTTGAATTAGGTGTCGTTGCAGATAATGTTGAAATGACTGTGGTAACTGAGGTGCTCATTGATGAGGAGAGCAGTTACTTTTGGTTATATCTCGTGCTCCTTCTGCTGTCGACTTTCTTATCGTGCTTTATTGTATTATCTCGATCCTTTATTCATTTTCTTGGGAATAGAAGCGGTCGGGGTGGTCCCTTCTGCCGGAAGGTTGCTATGTTACTATGGGGAGCGCGGTTGTGGTTGTTGCGCAGGGCAACTAGGCGTTACGGCTCTCTTGGAATCGTCAGGAAAAGGTATACGCGCGCTGGCATGCGCAAGTATTTGCGTGATTACAATTTTCAACTTGACGAGTGGGAGAGTGCGGTTGATTGCTCACCAGGCGAAGCCTTGATCGTTGCAGATCAAAGAATGTTCGTTAGAGCGGTGAACAAAATTGTGGACCTGGAAAGCGATTTTGACACTGTTGAAGGTGTCAAGAGAGTATTTCGAAACTACAATGTGCGCAATTATAAGGTTGAGAAGTGGTTAGACAAACATACGATACGTTCAGCGCTTGTTGTGCTGAATAGTGTCGTAAAAGATGACAATCTTACCTTGGGTGAAGGCATGCTGTCATTGGAGAGAGCGAGTTTTGACGACGTGTTAGGTTGCGACGTCGAGGCTCAGCGGAACGGCATGTACTTCAAGGACATTATTGCTCTTCAGTTTGCTTTTCGGCTTATAGGCACACCGGAATTCTCCGATTTCGTGATAGCTTACAAAGGTAATCTGTATCCATGCTATGTTGATGCAATCAAGAGACATAGTTTGATTCTGGAAAAGCATCATGTTGATGAAGATCAGAAAAAGAGAAACGAAAAGGCGCTGGAGTGGCTGCTCATGGCAGGACAGGGTGCTTTTGTGCTGTCTGCTGTTGTATCGACAGGCTTCGCGGCTGTGAAGATTCATAAGTGGATTAAAAGTCGCATGCTCCTGAGGGCGCTGCAGATGTTGCCATCTGTAGGAGGCGGAGGTAACGATGGTGGTGGTGGTTTCTCAGAAGAAGCTCTTCAAAGGTTTGCAGTTGGGCGAAACTTTGAAGAGCGTCTGGCGGCGCTGCACACTGGTTTAGATATGGCAGCTGAGAATCTAGAGAGTTTTTCACCGGAAGATATGCAAGTTGAGATCCAGCACGTTATTCAGGCTCACGTTGAGGGTTCTTACCATGCTGCGGATCTTCCGTTTTTAGGCAGTGTACCTAGTGTGGCTTCTGTTGTGGCGGACTCCGTTATTGATTCAAGTAGTGTTGGCAGTGATGTGTGTAGTGCAGACACGTCTATTTCGTTAGGCGTGGCTAGTGGCGCTAGGCGTCGTGCTGTTGCTAGCAACACGAGGATGTATCCAAGTGCTGGTGTTGTGAGTTACGGTAATTTTAAGTCACCGCGAGTTAGTGGTAAAAGTGGAATTGGTAGTATGAAGATGAAAATCGCTAGTAGGCCTATGGCGTCCTAGCGTGCTGTTTTTCATTCTACTCATTTTCATTTTTGTTACGTGTTCGCGTTTCGATTTAAAAGTACTATGTCTGCCTGTGCTTTTCATTCGTGTGACAAGTGTGTCGATGGGCCCAAGAGTGTTGTGTGCGTTAGTAAGTATCGTCACAGTGTTTATAAGGTTTTGGGTCTCTCTGTTGTTAAGTGTCGTTTGCCTGCTGACTGCGGTGTCAATTGCGGCATGCCTGCGGCGTTCGTTCTTGAAAATGGACATCCTAGGTTGACTCTCGATGGATACTGCGGTGAGAAGCACAAAGGTTATGTGATTTCTGGTGCATGGCGCCATGCGCAACTCCGGACTTTGAACGAAGAACTCGACAAACTGGAAAAGAGGGAGGAGTTTTTAAAAACTCAGATTAAGTTGCTCAGTGATAGCGCTAAGGCCAACACTGCTCCTGTTTACATTCCTAAGAAGATTAATCGTATGAAGGCGGAGGTTTGGGATGTTAATGATAAAATTCTGGATCGTAGCGCTGCTCTTGCAGACGTTATGGATGCTGTTGCGCTGGATTTATCGCCTGACAGTTCACCAAGGAAGTCTAAGCCTCTGTGATGTGTGTTTTACTTTACTACTGTATGTTTGTACACTGGAGTAGGTAACCAGGATAAAGAACCCTAGCGAAAATGTTTTGAAAAAATATTCTGTATGTATATAAATAAATGGGCAAGTCTGTTGCTTTGGCTATAGAGTAATCATACTCTTAAAACAATGACGTGGTCTTCGCGATAGAAGATGGTGCTTGTTATTTCACCTAAATCGAAATAACGAAAGGGCGTTCCACAGTACGTTTAAACTGTGGGTTTCAGCCTCTAGTTGCTATTGATGTAATATAAAAAAGATCAATATTCGACTATGATGAGGCGCGACTGGGTGTCGCGGCTGCTTTTACACAGCAGTTAGAAGGGGTTCAATTCCCCCCCCGAACCGGAGGGATATCTGGCCCA
